# Predicting spatial patterns of soil bacteria under current and future environmental conditions

**DOI:** 10.1038/s41396-021-00947-5

**Published:** 2021-03-12

**Authors:** Heidi K. Mod, Aline Buri, Erika Yashiro, Nicolas Guex, Lucie Malard, Eric Pinto-Figueroa, Marco Pagni, Hélène Niculita-Hirzel, Jan Roelof van der Meer, Antoine Guisan

**Affiliations:** 1 Department of Ecology and Evolution, University of Lausanne, Lausanne, Switzerland; 2 Department of Geosciences and Geography, University of Helsinki, Helsinki, Finland; 3 Institute of Earth Surface Dynamics, University of Lausanne, Lausanne, Switzerland; 4 Department of Fundamental Microbiology, University of Lausanne, Lausanne, Switzerland; 5 Bioinformatics Competence Center, University of Lausanne, Lausanne, Switzerland; 6 Vital-IT, SIB Swiss Institute of Bioinformatics, Lausanne, Switzerland; 7 Terrabiom, Essert-sous-Champvent, Switzerland; 8 Department of Occupational Health and Environment, Center for Primary Care and Public Health (Unisanté), University of Lausanne, Lausanne, Switzerland

**Keywords:** Microbial ecology, Metagenomics, Climate-change ecology

## Abstract

Soil bacteria are largely missing from future biodiversity assessments hindering comprehensive forecasts of ecosystem changes. Soil bacterial communities are expected to be more strongly driven by pH and less by other edaphic and climatic factors. Thus, alkalinisation or acidification along with climate change may influence soil bacteria, with subsequent influences for example on nutrient cycling and vegetation. Future forecasts of soil bacteria are therefore needed. We applied species distribution modelling (SDM) to quantify the roles of environmental factors in governing spatial abundance distribution of soil bacterial OTUs and to predict how future changes in these factors may change bacterial communities in a temperate mountain area. Models indicated that factors related to soil (especially pH), climate and/or topography explain and predict part of the abundance distribution of most OTUs. This supports the expectations that microorganisms have specific environmental requirements (i.e., niches/envelopes) and that they should accordingly respond to environmental changes. Our predictions indicate a stronger role of pH over other predictors (e.g. climate) in governing distributions of bacteria, yet the predicted future changes in bacteria communities are smaller than their current variation across space. The extent of bacterial community change predictions varies as a function of elevation, but in general, deviations from neutral soil pH are expected to decrease abundances and diversity of bacteria. Our findings highlight the need to account for edaphic changes, along with climate changes, in future forecasts of soil bacteria.

## Introduction

Soil bacteria form a large part of Earth’s biota and biodiversity [1, 2] and they have an integral part in ecosystem functioning [3, 4]. Perturbations in soil bacterial communities can influence whole ecosystems, for example via affecting nutrient cycles [5]. For that reason, it would be important to have forecasts of the future of soil bacteria upon changing environmental conditions [6–9]. However, they are still largely missing from future biodiversity assessments both at global (e.g., [10]) and regional levels (e.g., mountain ecosystems [11]).

Previous studies of top soil microbial biogeography have identified soil pH as the primary driver of bacterial communities, along with other edaphic (especially organic carbon, C) and climatic factors [12–15]. The effects of environmental changes on local edaphic conditions are, however, uncertain, and might equally result in increases or decreases of soil pH and organic C [16, 17]. Altogether, analogous to climate change [18], soil change scenarios would have to be developed that can build the foundation of future forecasts of soil bacterial communities [5, 19].

Regarding soil pH, a general acidification due to atmospheric sulphur (S) and nitrogen (N) depositions has been recorded worldwide [20]. In Switzerland, atmospheric deposition, mainly of S, increased from the 1960s [21] to the 1970s, before largely diminishing in the 1980s [22]. This deposition caused widespread but varying soil acidification, depending on the buffering capacity of soils [17]. In alkaline soils, the onset of measurable acidification from atmospheric deposition was delayed [20, 22]. As a result, they still continue to acidify whereas acidic soils are already recovering [23, 24]. The future trends of soil pH are thus determined by the interplay of soil type (e.g., alkaline vs. acidic) and current and future rates of atmospheric deposition, especially of N that still exceeds critical loads in Switzerland [25].

Atmospheric N deposition does not only influence soil pH, but also soil organic C content. Tipping et al. [26] presented evidence of a long-term increase in soil organic C due to N deposition. This trend contrasts with the empirical and experimental evidence of soils losing organic C in a warming world due to the intrinsic temperature dependency of the soil organic matter decomposition [27, 28]. Increasing temperature and decreasing soil moisture affect the rate of soil organic matter decomposition, enhancing C losses from soil to the atmosphere [29, 30]. On the other hand, rising CO_2_ levels and a warmer climate may increase mountain plant biomass production leading to increased littering, which would enhance the flux of organic C into the soil [31]. The latter process could offset projected soil carbon losses [32, 33], and the balance between the two processes may thus determine changes in organic C content and turnover in the soil [34]. As this matter is not settled, uncertainties are large on soil organic carbon change predictions [35].

It is to be expected that alterations in edaphic conditions are going to affect soil bacterial community structures, by altering the general growth conditions [36]. For example, total bacterial diversity is highest at neutral soil pH [12, 15] and bacterial abundance positively correlates with soil carbon availability [37]. Thus, soil acidification below neutral pH and amplified decomposition could decrease bacterial community diversity, whereas (slightly) higher soil alkalinity and C content could favour more bacterial species to flourish. Also changes in climate have been shown to lead to changes in bacterial abundances, diversity and community composition [9, 38–41]. All in all, the future of soil bacteria is uncertain and depends on interplay of multiple factors.

Here, we pursue forecasting effort for soil bacterial communities, and present initial findings based on the predictions of individual bacterial taxa driven by different future scenarios of both soil and climate. We use data from a well-studied temperate mountain region, first, to assess the variation in soil bacteria as a function of climatic, topographic and edaphic conditions covering large elevational and environmental gradients. Next, based on the literature and observed changes in edaphic conditions since the 1970s, we developed simple hypothetical sensitivity scenarios of future changes in soil pH and total organic carbon (TOC) content. Finally, we used combinations of edaphic and climatic change scenarios, together with the models obtained in the first step, to forecast potential future changes in bacterial communities. We benefitted from an analytical framework for species distribution and community modelling (SDM) frequently applied to assess and predict spatio-temporal occurrence of plant and animal species [42–44], adapted here to bacteria.

## Material and methods

### Study area

The data were collected from an intensively studied mountain area [12, 45–47], which is a priority area for transdisciplinary research at the University of Lausanne (http://rechalp.unil.ch and www.unil.ch/centre-montagne) located in the western Swiss Alps (46°10′–46°30′ N; 6°60′–7°10′ E), covering an area of ~700 km^2^ and spanning an elevation range of 425–3120 m a.s.l. Climatic conditions are heterogeneous, with annual mean temperatures and precipitation sums varying from 8 °C and 1200 mm at 600 m a.s.l. to −5 °C and 2600 mm at 3000 m a.s.l. [48], respectively, and solar radiation, debris accumulation and erosion vary according to topographic position, slope and aspect. The bedrock in this area is mainly calcareous with few occurrences of sandstone, schist, marlstone and phyllite. Soils range from slightly to moderately developed, with rarer occurrences of well-differentiated acid and poorly drained soils [49, 50]. Land cover is dominated by alpine grasslands, forests, glaciers and agricultural lands.

### Sample collection and 16S rRNA gene amplicon sequencing

To assess the bacterial communities across the study area, soil was sampled for 16S rRNA gene amplicon sequencing between July and September of 2012 and 2013 from non-forested quadrats of 2 × 2 m at 265 sites selected according to a random-stratified sampling design [51] considering elevation, slope and aspect strata. Detailed descriptions of the sampling and sequencing are published elsewhere [12], but briefly, each sample (500 g) consisted of five pooled and homogenized subsamples of top 5-cm layer soil collected with sterilized (ethanol and butane based lighter) tools. Triplicate DNA extractions (PowerSoil DNA isolation kit; Mo Bio Laboratories, Carlsbad, CA, USA) were conducted for each sample from 0.25 g of freshly collected ice-stored and sieved (2 mm) soil.

The V5 hypervariable region of the 16S rRNA gene was amplified in quadruplicate by polymerase chain reaction (PCR), using the primer set 784DEG and 880RDEG [52] appended at the 5′ ends with one of 40 pairs of 3- to 6-base forward and reverse barcodes. The PCR products were purified, pooled, annealed with adapters and a third barcode for library preparation, and finally paired-end sequenced (2 × 100 nt) on HiSeq 2500 platform (Illumina) at the Genomic Technology Facility of the University of Lausanne. We previously showed that the selected primers, initially published in an oral microbiomes study, have an excellent taxonomic coverage for soil biogeography studies ([12, 53] and unpublished preliminary analysis). Further, although the hypervariable regions selected as a proxy for the full 16S rRNA gene have shifted over the years, the V5 region used in the present study has been demonstrated to illustrate plausible trends of soil bacteria communities [12, 53].

### Bioinformatic processing

For the de novo (DN) approach, sequenced reads were demultiplexed and barcodes were removed using a custom-made perl script. The 5′ ends of each paired-end sequence were matched against the IUPAC sequence composed of the adaptor, spacer and forward and reverse primer barcodes associated with each sample, allowing for at most one mismatch for each end. Sequence pairs were attributed to a given sample only when both ends had a match, and when the attribution was unambiguous (i.e., no other candidate sample obtained an equivalent or lower number of mismatches). To recover the sequence of each 16S rRNA gene fragment, the 3′ overlap of each sequence pair was assessed. Only the 16S rRNA gene fragments with overlapping sequence stretch >98% were retained, and the nucleotide with the best quality score was kept. Finally, we trimmed the 5′ and 3′ ends of the fragments to remove the sections originating from the region of the primers designed with degenerate nucleotides, which artificially inflate sequence diversity and affect the subsequent clustering steps. Thus, all fragments start with TTAGATACCC and end with C. The 16S rRNA gene fragments were then dereplicated by combining all strictly identical fragments into unique ones to obtain zero-radius operational taxonomic units (zOTUs [54]) with a corresponding abundance equal to the number of fragments with this unique sequence. Finally, we removed the zOTUs with total count among all samples <100.

Since the resulting number of zOTUs exceeded the computational power available for the spatial analyses, we clustered them by an all-against-all sequence alignment using Align0 [55]. The resulting similarity scores were then converted to distances using the following formula:

$${\mathrm{dist}}\left[ {i,j} \right] = {\mathrm{sim}}\left[ {i,i} \right] + {\mathrm{sim}}\left[ {j,j} \right] - 2{\mathrm{sim}}\left[ {i,j} \right]$$

Operational taxonomic units (OTUs) were then obtained by agglomerating all zOTUs, which could be regrouped using a given distance cut-off via single linkage. Here, the clustering distances of 20, 40 and 60 were chosen as the shortest distances with notable decreases in number of resulting OTUs (see Appendix 1). Multiple distances were chosen to test the effect of clustering.

Phyla affiliation of OTUs was obtained by first annotating the zOTUs by comparison against the full SILVA taxonomy database version 132 (SILVA_132_SSURef_tax_silva.fasta.gz [56]; for details see Appendix 1). The phylum of each OTU was defined as the mode of phyla of zOTUs clustered into the OTU. The phyla that did not represent the globally ten most abundant phyla [57] were grouped together.

To account for varying sample library sizes, we incorporated an offset term in the models (see ‘Spatial analyses’ below and McCarthy et al. [58]). For comparison, we also prepared a normalized dataset by first rarefying the samples to the lowest total count of zOTUs (131566) across all samples prior to OTU clustering with the ‘rarefy_even_depth’ function without replacement from the phyloseq R-package [59].

Since we cannot explicitly ensure that our clustered sequences represent individual bacterial species (because of microvariation among multiple 16S rRNA gene copies within a single bacterial strain), we repeated clustering with a closed-reference (CR) approach to assign sequences to known bacterial genera, as in Yashiro et al. [12]. In brief, the demultiplexing process included a quality-filtering step that retained only the sequences that had 100% matching adaptor, spacer and forward and reverse primer barcodes. Sequences were then clustered into OTUs in QIIME v.1.7.0 [60] at the 97% similarity threshold using the gg_13_8 database from Greengenes as a reference [61, 62]. The total number of reads per sample was normalized to 99,618 (i.e., the lowest total count of sequences across all samples) by rarefaction using random selection without replacement. Since species level information was only available for <4% of the OTUs, we used genus level taxonomic annotation that was available for 27% of the OTUs. We acknowledge the existence of more up-to-date CR approaches and reference databases. However, we opted for the aforementioned workflow because part of such dataset had already been successfully used to assess soil microbial biogeography in the same study area [12, 53]. Therefore, demonstrating consistency in our observations between the DN and CR approaches allows us to use the previous ecological findings as a tool to validate our SDM-based findings.

In summary, we used seven OTU-per-site datasets for the spatial analyses: (1) three datasets based on DN approach where zOTUs were clustered at distances of 20, 40 and 60 (hereafter coded as DN: cl20, cl40 and cl60); (2) three datasets based on DN approach and normalized read counts clustered at distances of 20, 40 and 60 (DNn: cl20, cl40 and cl60); and (3) one dataset based on CR OTU picking and clustered to genera (yet in the text, genera are also referred as OTUs). In all cases, prior to analyses, we removed the OTUs occurring in <21 sites to ensure more confident model parameter estimation (see Table 1).

**Table 1:** Count of OTUs within different datasets.

Dataset	Clustering	*n* of zOTUs/OTUs	*n* of OTUs^a^	*n* of modelled OTUs (occurring in >20 sites^a^)	*n* of OTUs included in predictions^b^
DN	Original	60567^c^/59344^a^ zOTUs			
DN	cl20		19,267	16,167	15,162
DN	cl40		9649	7836	7322
DN	cl60		6522	5258	4905
DNn	cl20		19,267	8961	8757
DNn	cl40		9649	4227	4127
DNn	cl60		6522	2836	2762
CR	Original	15193^c^/15103^a^ OTUs			
CR	Genus		736	376	362

**Fig. 1:**
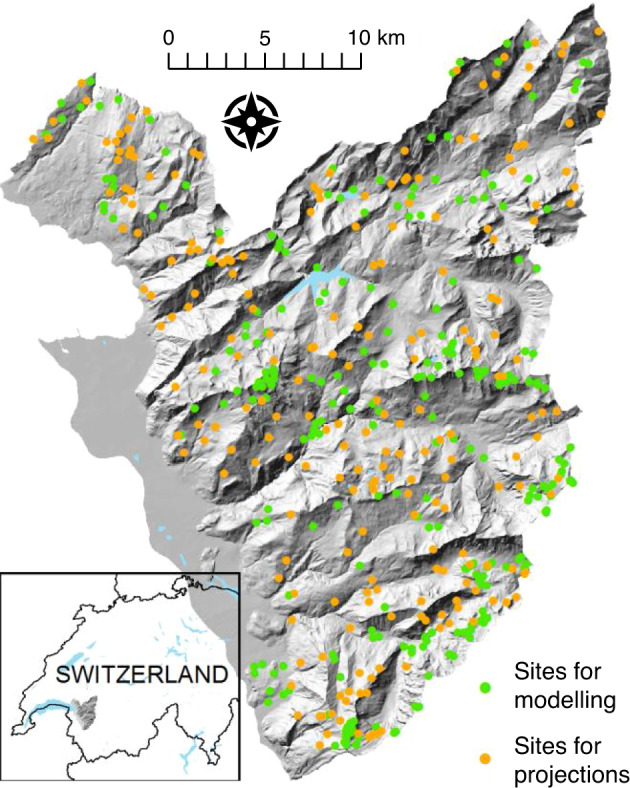
Locations of study sites and area in western Swiss Alps. 255 sites of 16S rRNA gene amplicon sequencing for which environmental variables could be derived were used for modelling. For the projections, an independent set of 229 sites was used.

From the originally sampled 268 sites, sequencing was successful for 265 sites. Out of these sites, environmental variables could be obtained for 255 sites which were used for modelling. For the predictions, an independent set of 229 sites was used (see Fig. 1).

*DN* de novo clustering of OTUs, *DNn* de novo clustering of OTUs based on normalized counts of zOTUs, *CR* close-referenced OTU picking and clustering.

^a^Within 255 sites.

^b^Based on GAMnb.

^c^Within 265 sites.

### Environmental data

Nine environmental predictors, representing the three main groups of abiotic habitat factors in the study area: climate, topography and soil were included as predictors in the models. From an initial set of 79 predictors, we chose three variables per group based on their explanatory power while controlling for multicollinearity (see details in Appendix 2): temperature of the coldest quarter (°C; *T*_coldQ_), precipitation of the driest month (mm; *P*_dryM_), annual temperature range (K, *T*_range_), potential annual solar radiation (KJ; sRad, governed by topography in the study area), topographic position index (unit-less, indicating a gradient from valley bottoms to ridge tops; TPI), slope angle (°), soil pH, logarithm of TOC content (%; TOC_log_) and clay content (%). These environmental variables could be obtained for 255 of the sampling sites used for 16S rRNA gene amplicon sequencing (see Fig. 1).

To independently apply our predictions of bacterial communities under current conditions and future scenarios in the study area, we used a separate dataset of 229 sites (see Fig. 1) for which we could obtain the same environmental parameters. Environmental variables representing current conditions for these sites were derived as described in Appendix 2 with the exception of deriving edaphic data from soil samples collected 2012–2013 ± 3 years (e.g., from Dubuis et al. [63]). Three climatic variables were used for future predictions and were derived under the IPCC A2 scenario for the time period 2047–74, based on the official scenarios available for the study area with 25 × 25-m resolution [meteoswiss.ch and ref. [64]]. For topography and clay content, no future change between now and the coming decades is expected.

Future estimates of pH and TOC were derived through extrapolation from historical changes in the same study area [65]. As, unlike for climate, no established soil change scenario is available for the Alps, we applied the same sensitivity approach used in early climate change impact modelling studies [66], by changing the value of soil parameters according to representative past measurements. In brief, the slopes of temporal pH and TOC changes were calculated from soil resurvey analysis incorporating 112 paired samples from 1970 and 2016 (Appendix 2). For both pH and TOC, the mean of slopes indicated increases, with no relation to elevation. Then, to derive ‘increase’ scenarios for pH and TOC, we used the derived mean slopes to extrapolate to year 2060 from the measured (2012–2013 ± 3 years) values of the 229 sites. Alternative ‘decrease’ scenarios were developed similarly but with the inverted mean slopes. The pH_inc_-scenario assumes a future increase of 0.3 pH units (i.e., representing a scenario where soil acid neutralization phase continues). In contrast, the pH_dec_-scenario assumes a decrease of 0.3 pH units (i.e., a scenario representing acidification). For TOC, 3.2% points were added and subtracted from the current values for the increase and decrease scenarios, respectively. TOC_inc_ represents a scenario with increased C stocks in soil due to enhanced plant productivity and litter, whereas TOC_dec_ points to a scenario with decreased C stocks as a result of amplified decomposition. Finally, pH_now_ and TOC_now_ scenarios assume no change in soil pH and TOC between now and the year 2060.

### Spatial analyses

To assess bacterial communities now and in the future, we implemented an analytical framework based on species distribution models (SDMs [42–44]), but adapted to relative OTU abundances instead of species occurrences (see Appendix 3). Abundance of each OTU was first modelled as a function of the nine environmental predictors using a generalized additive model (GAMp) with spline smoothers from R-package mgcv [67] and a gradient boosting model (GBM) with 2000 trees, interaction depth of 3 and shrinkage of 0.01 from R-package gbm [68], both with Poisson distribution (suitable for sequence counts). Because preliminary analyses indicated overdispersion for several OTUs [69, 70], we additionally fitted GAM with a negative binomial (nb) distribution (GAMnb). nb distribution is not available for GBM, but the benefit of GBM over GAM is that it automatically incorporates statistical interactions among the predictors. For non-normalized OTU datasets, we added the logarithm of the total sequence count per site (prior to removing sequences with <100 counts) as an offset term to control for the varying library sizes [58]. With an offset term, a rate (here, sequence count of an OTU proportional to the total count of sequences in a site) is modelled instead of counts of sequences.

The model fit (i.e., how well the nine environmental variables together explain the variation in abundances) per OTU was assessed by the correlation between observed and fitted abundance values (cor_expl_). The relative importance of the predictors per OTU was determined using predictor shuffling for GAMp and GAMnb (Appendix 3) and following Friedman [71] for GBM. To evaluate the prediction performance of the models, we trained and evaluated them ten times by randomly assigning 80% and 20% of the sites for model calibration and evaluation, respectively, assuring that each site was used on average eight times for calibration and twice for evaluation [72]. For each OTU, we then calculated the correlation between its observed abundance and the mean of the two predicted abundance values in the evaluation sites (cor_pred_).

From subsequent projections, we excluded the OTUs for which both cor_expl_ and cor_pred_ were weak (<0.2; sensu Evans [73]). We used both cor_expl_ and cor_pred_ as the random splitting of data may result in distribution of abundance values strongly varying between training and evaluation datasets potentially resulting in low cor_pred_ even with sound cor_expl_. For the projections of the OTUs of the DN datasets, we used the median of library sizes as the offset term.

Response curves (sensu Elith et al. [74]) were produced for each OTU and environmental variable. Response curves are projections of OTUs’ abundances in an environmental space where the variable of interest varies from low to high and all other predictors are fixed (here, to the observed median values across the 255 sites). This way, the effects of variables other than the one being investigated can be controlled when assessing the relationship between an OTU and an environmental variable (however, note that the OTUs specialized to extreme environmental conditions might not show any variance in median conditions). The variation of bacterial communities along the environmental gradients was then summarized by stacking the response curves of individual OTUs, and calculating (i) the proportion of OTUs with higher than median abundance, (ii) the Shannon index and (iii) relative abundance of phyla.

Changes in bacterial communities were assessed from the projections of abundances of individual OTUs in the 229 independent sites under current environmental conditions and the nine possible combinations of the climatic (IPCC A2) and edaphic scenarios (pH_inc_, pH_now_, pH_dec_, TOC_inc_, TOC_now_ and TOC_dec_). Based on the current and future projections of individual OTUs, we calculated for each site: (i) the proportions of OTUs with increase and decrease in predicted abundances, (ii) the change in Shannon index and (iii) the relative abundance of phyla. Some sites used for projections, especially under future scenarios, contain environmental values falling outside the environmental conditions covered by the training data. In these sites with non-analogous environmental conditions, the models need to extrapolate, potentially decreasing the reliability of predictions. Thus, we identified all sites with environmental values above the maximum or below the minimum of each variable in the training data (Appendix 2).

## Results

The DN approach recovered 60,567 zOTUs (occurring >100 times across all sites; Table 1). After clustering, 16,167, 7836 and 5258 DN OTUs (at distances 20, 40 and 60, respectively) and 376 CR genera were available for the modelling (i.e., occur in at least 21 of the 255 sites). DNn-based datasets (i.e., based on normalization) have ~50% less OTUs available for modelling at all clustering distances than DN-based datasets (Table 1). DN and DNn-based datasets harbour rarer OTUs and higher median elevational optima than the CR dataset (see Fig. S1 in Appendix 4).

**Fig. 2:**
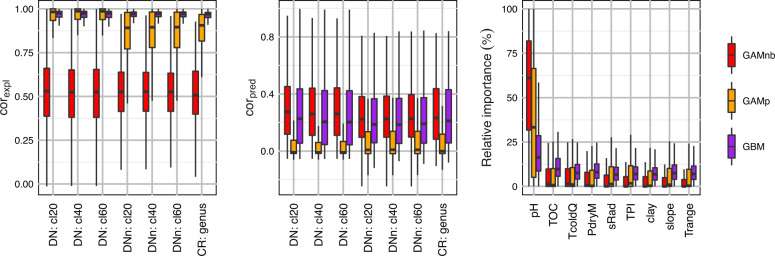
Model performance of GAMnb, GAMp and GBM as measured with correlation of observed and fitted abundances (cor_expl_) and the correlation of observed and predicted abundances (cor_pred_) for modelled OTUs of different datasets, and relative variable importance (%) based on DN: cl40. DN de novo, CR closed-reference, cl20 clustering distance of 20, pH soil pH, TOC logarithm of total organic carbon content (%), TcoldQ temperature of the coldest quarter (°C), PdryM precipitation of the driest month (mm), sRAD potential annual solar radiation (KJ), TPI topographic position index, clay clay content (%), slope slope angle (°) and *T* range annual temperature range (K).

According to cor_expl_ and cor_pred_, model performance is mostly similar among the different datasets, but varies among the OTUs and models (Fig. 2 and S2–S4 in Appendix 4). In general, model performance is better for frequently occurring than for rare OTUs (Figs. S5–10 in Appendix 4). GAMnb demonstrates the best overall performance (median cor_expl_ ~0.53 and cor_pred_ ~0.26). The large differences in median cor_expl_ and cor_pred_ of the GBM (~0.97 and ~0.21, respectively) and GAMp (~0.97 and ~−0.01) indicate overfitting, with GAMp additionally failing to predict the abundances of most OTUs (negative median cor_pred_). The following results are thus based on the GAMnb, whereas results for GBM and GAMp are shown in Appendix 4.

**Fig. 3:**
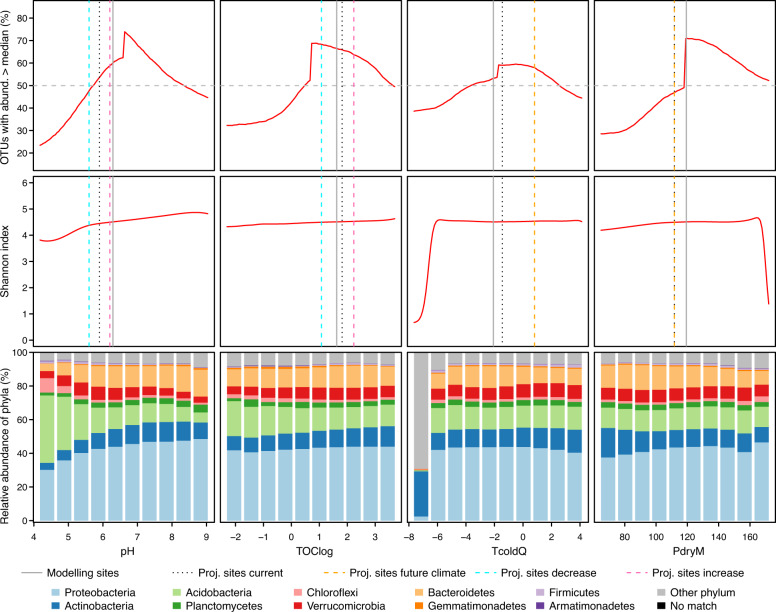
Variation of bacterial assemblages (based on DN: cl40) along the four most important environmental gradients (see Fig. 2) calculated from OTUs’ response curves created based on GAMnb (for other datasets and GBM, see Figs. S12–15 in Appendix 4). Top row: proportions of OTUs with abundance >median (%). Middle row: variation of Shannon index. Bottom row: relative abundance of phyla (%). Vertical lines indicate median environmental conditions of different environmental data and scenarios: modelling sites = current conditions in the sites used to train the models, proj. sites current = current conditions in the sites used for projections, proj. sites future climate = future climatic conditions in the sites used for projections, proj. sites decrease = future edaphic conditions under the ‘decrease’ scenario in the sites used for projections, proj. sites increase = future edaphic conditions under the ‘increase’ scenario in the sites used for projections.

Irrespective of the dataset, pH has the highest median relative importance across all OTUs, with other variables playing a part in the models of some OTUs (Fig. 2 and S11 in Appendix 4). Along the environmental gradients, the abundances of OTUs were higher than their medians, on average, where pH is 6–8, TOC and clay contents are 2–40% 0–25%, respectively, temperature conditions are not extreme and precipitation is 120–170 mm (Fig. 3 and S12 in Appendix 4). Shannon index increases together with pH, very slightly with TOC, is lowest under the coldest and wettest conditions, and decreases as a function of clay and slope steepness (Fig. 3 and S13 in Appendix 4). The relative abundance of phyla varies especially along pH gradient (Fig. 3 and S14–15 in Appendix 4). With increasing pH, the relative abundance of Acidobacteria and Chloroflexi decreases, whereas the relative abundance of Proteobacteria, Actinobacteria and Bacteroidetes increases.

Projections to nine different combinations of future climatic and edaphic change scenarios indicate mainly consistent trends among the different datasets, and some variation between GAMnb and GBM (Figs. S16–50 in Appendix 4). The trends in predicted changes are mostly similar when considering only sites with analogous environmental conditions instead of all sites (Figs. S23–43 in Appendix 4). Climate change alone is predicted to decrease the abundances of most OTUs at lower elevations and to mainly increase the abundances at higher elevations (Fig. 4). Simultaneous decrease in pH would amplify the proportions of OTUs with decreasing abundance, whereas simultaneous increase in pH would amplify the proportions of OTUs with increasing abundance especially at mid elevations. Changes in TOC would have minor mediating effect on changes in abundance resulting from changes in climate and/or pH.

**Fig. 4:**
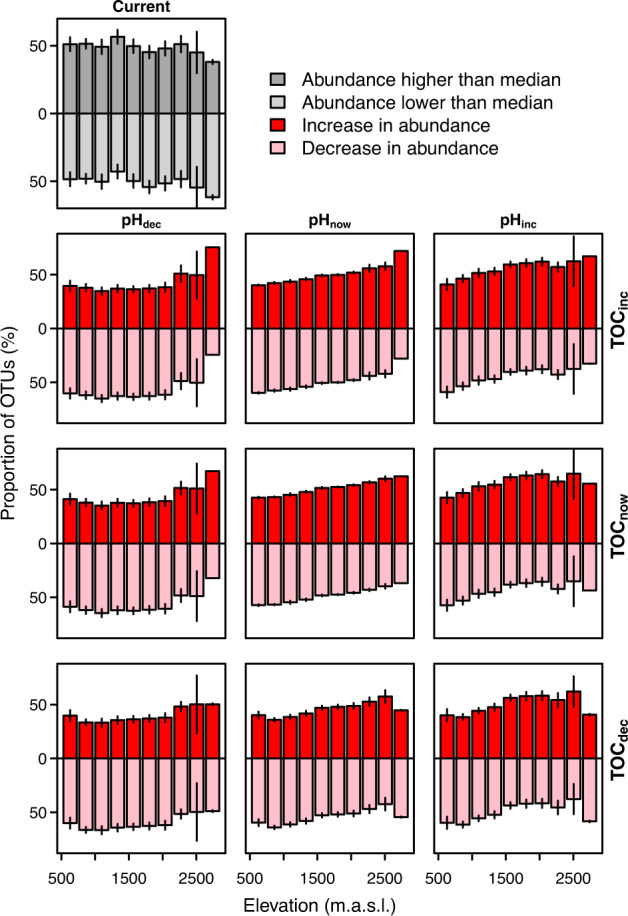
Based on the projections to 229 sites and shown against the elevation, proportions of OTUs (%) having higher than median abundance under current environmental conditions (single plot at the top row) and change in abundance between current and future environmental conditions (3 × 3 panels; the plot in the middle shows the sole effect of changing climate, while the surrounding plots show the effects of climate change plus the different combinations of decrease, increase or stability in pH and TOC). Bars indicate mean of sites with 95% confidence intervals shown with error bars. The figure is based on DN: cl40 and GAMnb. For other datasets, GBM and details per site (including all sites vs. the sites with analogous environmental conditions), see Figs. S16–36 in Appendix 4.

**Fig. 5:**
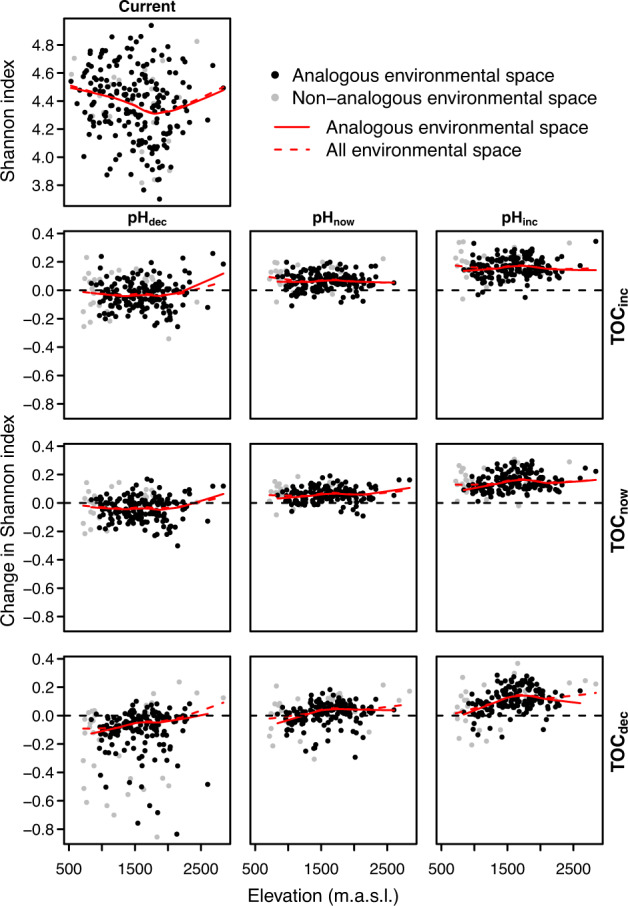
Based on the projections to 229 sites and shown against the elevation, Shannon index under current environmental conditions (single plot at the top row) and changes in Shannon index between current and future environmental conditions (3 × 3 panels; the plot in the middle shows the sole effect of changing climate, while the surrounding plots show the effects of climate change plus the different combinations of decrease, increase or stability in pH and TOC). Dots indicate the sites (‘outliers’ [+/−1.58 × IQR/$\sqrt {299}$] are removed), with lines showing the trends along elevation as LOWESS (locally weighted scatterplot smoothing). Sites with analogous environmental space (in black) indicate the sites for which all environmental variables are within the range included in the model training (i.e., the 255 sites used in modelling), whereas the non-analogous sites (in grey), contain environmental values exceeding the environmental conditions covered by the training data meaning that the model needs to extrapolate in these sites. The figure is based on DN: cl40 and GAMnb. For the other datasets and GBM, see Figs. S37–43 in Appendix 4.

The Shannon index is predicted to slightly increase by climatic changes alone, with more pronounced increases at higher elevations (Fig. 5). The predicted increases would be further amplified by simultaneous increase of pH and TOC, whereas decreasing pH, especially together with decreasing TOC, is predicted to decrease diversity, especially at low to mid elevations (Fig. 5).

**Fig. 6:**
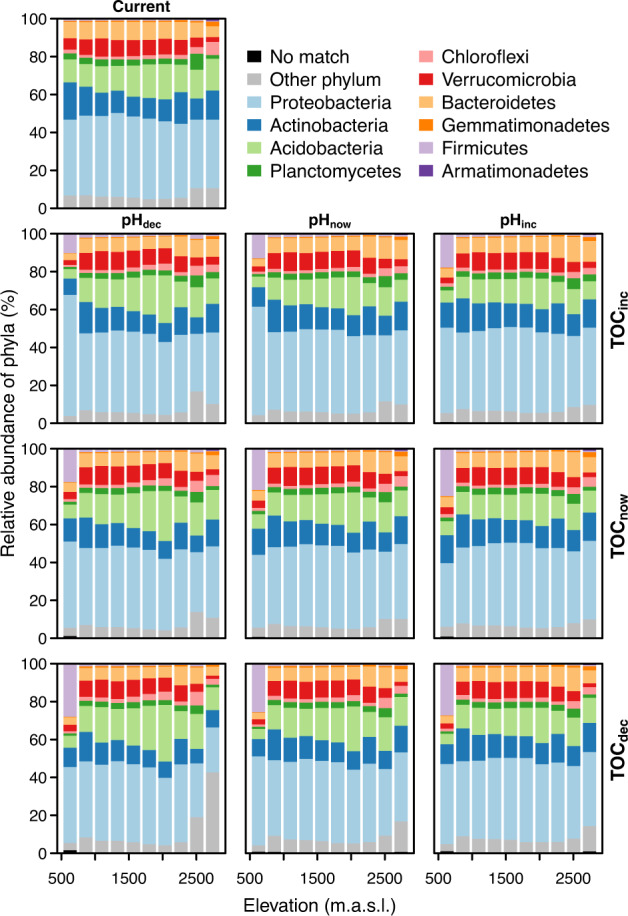
Based on the projections to 229 sites and shown against the elevation, relative abundances of phyla (%) under current (single plot at the top row) and future environmental conditions (3 × 3 panels; the plot in the middle shows the sole effect of changing climate, while the surrounding plots show the effects of climate change plus the different combinations of decrease, increase or stability in pH and TOC). Bars indicate averages of sites. The figure is based on DN: cl40 and GAMnb; for the other datasets and GBM, see Figs. S44–50 in Appendix 4.

The relative abundance of phyla is predicted to only slightly change under different future scenarios (Fig. 6). Changes in pH would govern the relative abundance of especially *Acidobacteria* around 2000 m a.s.l. Climatic changes with simultaneous decrease in pH and TOC would increase the relative abundance of phyla that do not represent the ten most globally abundant phyla. The relative abundance of *Firmicutes* at the lowest elevations increases under all combinations of future scenarios, the most with changing climate and decreasing TOC, yet part of this increase could be an artefact due to low number of sites and non-analogous temperature increase at the lowest elevations.

## Discussion

Assessments of the influence of environmental changes on species distributions have largely focused on macroscopic species [75, 76]. Fewer studies have investigated soil bacteria and their future changes, addressing mainly the diversity, community structure or certain dominant phyla of bacteria [39–41, 77]. Especially, studies at OTU level are scarce [9] and should also incorporate edaphic changes along with climatic ones, since soil pH and thus acidification and alkalinisation, are important drivers of soil bacteria [12, 15, 78, 79]. To fill this gap, we developed simple hypothetical sensitivity soil change scenarios based on observed historical changes in pH and organic carbon content, and combined them with climate change scenarios into SDM-based forecasts of potential changes in OTU distributions. Model performances indicated that the nine environmental factors used, especially soil pH, explain and predict at least part of the abundance distribution of most OTUs in our study area. Our models thus provide support for the previous results of Ladau et al. [9], Delgado-Baquerizo et al. [57] and Fierer et al. [80], that most bacterial taxa have clear environmental requirements (i.e., ecological niches/envelopes [81]). Assuming that these environmental niches would be conserved in the future [82, 83], we further show that different combinations of changes in climate and soil would affect the spatial distributions of bacteria distinctly [9].

Previous studies have reported the central role of pH in defining distribution of some higher clades e.g., [13, 14], or community properties such as richness e.g., [12, 15], biomass e.g., [78, 84], or structure/composition e.g., [79]. Here, we showed more specifically that pH also strongly governs the distribution of abundances of individual OTUs in space over a large region with wide environmental gradients. The strong role of pH is also visible in our future forecasts, where decreasing or increasing pH affects the changes in abundance distribution of OTUs predicted under climatic changes alone. In general, warming climate is expected to be beneficial for the majority of OTUs, leading to higher total bacterial diversity at mid to high elevations. Simultaneous acidification would, however, mostly cancel out the effects of warming, except at the highest sites where the soil is relatively alkaline, whereas simultaneous alkalinisation would further benefit most OTUs, apart from *Acidobacteria*, and total diversity. Note, however, that the strong increase of relative abundance of *Firmicutes* at the lowest elevations could be an artefact resulting from the low number of sites and non-analogously warm future conditions at the lowest elevations, but a formal study of this group’s distribution beyond our study area borders (i.e., assessing whether its abundance increase further toward warmer conditions) would give the final answer to this question.

These forecasts are also anticipated by the response curves. For example, both coldest and warmest temperatures appear as disadvantageous for most OTUs. Further warming at low elevation with milder climate results in decreasing abundances being predicted for most OTUs, whereas warming at cold high-elevation sites benefit many OTUs (see also Nottingham et al. [85] predicting that warming amplifies soil bacterial activity the most at the high elevations). Similarly, for instance, the lowest soil pH values are currently recorded at mid elevation sites, where further acidification could lead to sub-optimal conditions for growth or maintenance of many bacterial OTUs (except *Acidobacteria*; [15]). Congruently, increasing soil pH would be beneficial for bacteria (apart from *Acidobacteria*) in acidic sites at mid elevations. Under warming climate and increasing pH, bacteria could thus show similar patterns as plant communities migrating toward higher elevations [19]. Finally, the forecasted changes in bacteria may also partly result from pH change scenarios mimicking a move-away from (or a move-toward) acidic and anoxic wetland-type habitats, which tend to contain a more specialized and restricted bacterial community composition [53].

Interestingly, both decreasing and increasing TOC levels are predicted to slightly decrease the abundance of most OTUs and Shannon index. The negative effect of decreasing TOC is likely related to the reduction of available resources for bacteria [30], whereas excessive TOC might mimic wetland conditions where the water-logging-related anoxic conditions lowers the abundances of OTUs despite the accumulated organic matter [12]. In general, however, the effect of changing TOC on bacterial communities was much less than the effect of changing pH. This suggests that the TOC changes anticipated in our study area and scenarios are not enough to drastically affect the stability of the mountain grasslands soil bacterial communities. Indeed, the response curves show that most OTUs can thrive as long as the TOC content is >2%, and not many sites would appear with TOC contents <2%, even under the TOC_dec_ scenario. However, further work is needed to assess the critical level of changes in TOC affecting the rates of microbial organic matter mineralization and other metabolic processes that are commonly associated with fresh organic matter in in situ experiments [86]. For example, soil depth profiling studies of stable microbial communities have shown that decreasing TOC can result in a decrease in bacterial diversity [87].

Altogether, our study emphasised the role of pH in defining not only the current but also the future abundance distribution of soil bacterial communities. Thus, the future of soil bacteria should be strongly determined by changes in human-related activities such as nutrient deposition [88, 89] and land use [90] that drive concurring changes in soil pH. Here, in the absence of established soil change scenarios developed by soil scientists, we created simple but realistic scenarios of future edaphic conditions by mimicking the magnitude of changes observed in the study area over the last five decades [65] while acknowledging both potential increase and decrease of soil pH and organic content [24, 29]. This is similar to the sensitivity approach used in early regional climate change impact studies [66], but obviously, future forecasts of soil bacteria communities would benefit of more advanced soil scenarios. These should take into consideration that the changes in edaphic conditions are likely not as constant across space as assumed here, due to the variance in parent material and the interplay of drivers [89, 91]. To build such multifaceted scenarios, in addition to soil science expertise, improved edaphic maps would be needed [92]. Also, we acknowledge that other drivers, for example aboveground vegetation communities [53, 93], microclimate [94], snow [14] and biotic interactions among bacteria taxa [95], contribute to define the current and future distributions of bacteria [96]. Assessing the influence of these factors on soil bacteria and incorporating their future changes along with climate and soil scenarios would be an important next step for more realistic understanding and forecasts of future soil bacteria [97]. Finally, studies that include the functional identity of bacteria are needed to anticipate the influence of bacterial community changes on ecosystem functioning, especially nutrient cycling and carbon emission [5, 98].

SDMs have so far been mainly applied to occurrence data of plant and animal species under the assumption of capturing their realized niches (see e.g., Araújo et al. [99] and references therein). The ability of our models to explain and predict the abundance distributions of bacterial OTUs in space indicates, as also explored in Ladau et al. [9] and Delgado-Baquerizo et al. [57], that individual belowground microorganisms too have an envelope/niche of environmental tolerances that can be captured, at least partly, by climatic, topographic and edaphic factors (ref. [100] and see Smith et al. [101] for a discussion of the niche concept above and below the species level). However, applying SDM on DNA-based data required methodological adaptations, which would benefit from further developments. Here, we tested some adaptations (i.e., varying bioinformatics pipelines and modelling approaches) to assess different procedures and provide insights for future niche modelling of microorganisms.

First, the similar model performance and projections between DN and CR datasets demonstrated that both approaches recovered sets of OTUs with similar environmental responses. However, DN recovered remarkably more OTUs and the elevation optima of these OTUs were generally higher than the optima of CR-based genera. This indicates that high-elevation bacteria tend to be under-represented in reference databases. Second, the clustering distance of DN data (here 20, 40, 60) or use of CR genera did not affect model performance nor predictions. This stability gives support for niche conservatism across taxonomic levels [102], implying that biogeographical patterns of soil bacterial communities would persist across taxonomic resolutions [13, 103]. Third, the models incorporating offset terms to accommodate for varying library sizes (DN) showed slightly better performance than the models based on normalized data (DNn). This is likely due to the higher number of non-zero counts of DN-based OTUs (see Fig. S2 in Appendix 4) that results in better model fits (see Figs. S5–10 in Appendix 4). McCarthy et al. [58] also suggested the use of offset terms with some alternative approaches. The downside of offset terms, on the other hand, is that their approximation for projections is not straightforward and they set a somewhat arbitrary constraint. Thus, while the trends in the future forecasts should remain correct, assessment of absolute changes is conditional to the choice of the offset. All in all, establishing a standard strategy, based on ecological and statistical foundations, on how to handle relative abundance in SDMs would be fundamental for their wider use in microbial ecology. This should also include an assessment of the effect of likely presence of relic DNA (i.e. remains of dead bacteria) within the soil samples and other biases innate to microbiomes, that may bias the interpretations of spatial patterns and their ecological drivers [104]. Fourth, GAMnb outperformed GAMp and GBM, indicating that accounting for overdispersion improves model performance [105]. Further improvement might be achieved from incorporating interactions among the environmental predictors [106] since projections of GAMnb and GBM show some difference that might be due to interaction terms that are considered in GBM but not in GAM. Interaction term allows the magnitude and direction of the effect of one variable to vary as a function of another variable. The differences in projections among the datasets and models might also be related to algorithms and, for example, their capability to handle zero-inflated data [107]. Finally, while a ‘species’ is a natural modelling unit to assess persistence of plant and animal populations in a region [108], defining a ‘species’ or ‘population’ is more challenging for bacteria [109, 110]. Here, we used OTUs (and genera) under the assumption that the clusters of similar sequences show distinct responses to environmental factors [111], but modelling functional groups or some other grouping of bacteria could be ecologically more relevant [112, 113]. Further, it remains to be assessed whether their ecological niches are conserved in space or time [83, 103], namely, whether the environmental tolerances of bacterial entities persist under changing conditions resulting in range changes [114, 115], or whether the adaptive capacity of bacteria could allow them to shift their environmental tolerance (and thus their niches), resulting in static distributions [116].

## Supplementary information

Appendix 1

Appendix 2

Appendix 3

Appendix 4
