# Supplementary material for: Predicting spatial patterns of soil bacteria under current and future environmental conditions
Source: ISME J. 2021 Mar 12;15(9):2547–60. doi: 10.1038/s41396-021-00947-5 (PMC8397778; doi:10.1038/s41396-021-00947-5)
Supplement: Supplementary file 1 — Appendix 1 [file 41396_2021_947_MOESM1_ESM.pdf]

Supplementary material to Mod et al. *Predicting spatial patterns of soil bacteria under current and future environmental conditions.* ISME j.

Appendix 1: Approximating taxonomic rank of De Novo clustered OTUs at different clustering distances

To approximate which taxonomic ranks the different clustering distances (20, 40 and 60, i.e. OTUs) represents (Figure 1), we assigned each ASV with taxonomic annotation. Annotations were obtained by the Ribosomal Database Project (RDP) Classifier (Wang et al. 2007) using the 16srrna option and by comparison against the full (e.g. not the 99% non-redundant version) SILVA taxonomy database version 132 (SILVA_132_SSURef_tax_silva.fasta.gz; (Yilmaz et al. 2014)). RDP provides taxonomic ranks from domain to genus (domain->phylum->class->order->family->genus; in the case of a missing rank, lower rank is reported), with confidence estimates for each annotation. SILVA provides taxonomic ranks from domain to species (domain->phylum->class->order->family->genus->species; in the case of a missing rank, NA is given), with a respective number of hits in the database. From the resulting assignments we removed from each rank the entries containing terms “uncultured", "metagenome", “enrichment”, "incertae”, “candidat", "unknown", “uncultivated", “unidentified" or “possible". From the RDP based assignments, we further removed entries with <97% confidence estimate. For SILVA assignments, out of the multiple matches in the database, we kept the taxonomic assignment with most hits. Then from each rank we removed the assignments that had hit counts lower than 0.97*hit count of the sequence in the database. No other cleaning, filtering or quality controlling was done for the assignments, as this part of the analyses was only used to approximate the level of taxonomic ranks that the OTUs represent. For the approximation, we used two approaches, separately done for RDP and SILVA based annotations.

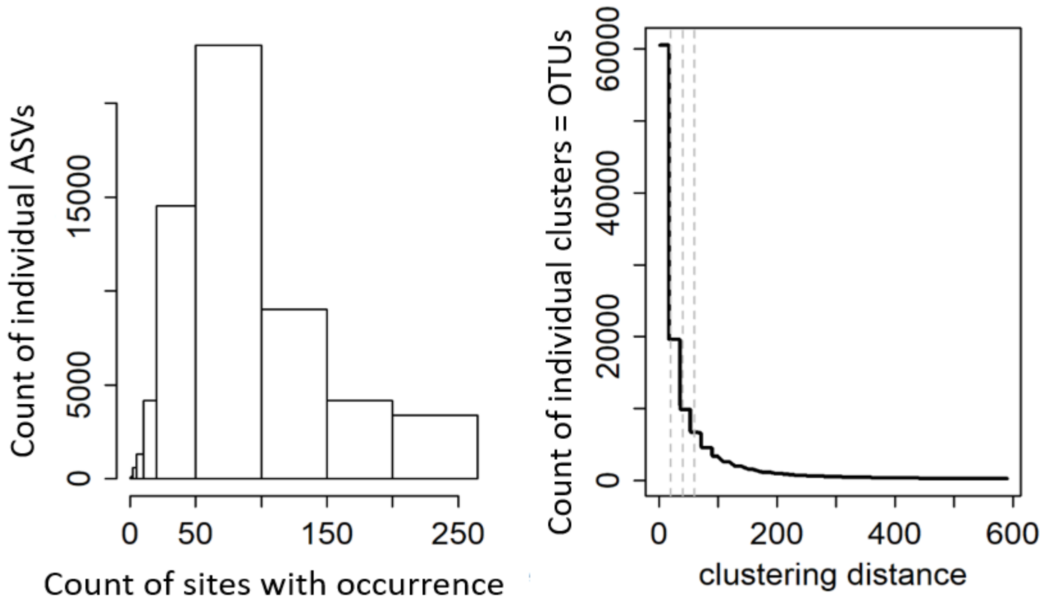

Figure 1. Counts of occurrences of 60567 individual ASVs among the 265 sites (left) and counts of clusters of sequences (=OTUs) with different clustering distances (right). For the spatial analyses, we clustered the ASVs, as performing spatial analyses for 60567 individual sequences would have exceeded the computational power available. The chosen clustering distances are 20, 40 and 60 (shown with grey dashed lines) as with these distances the number of clusters decreases notably.

For the first approach, at each clustering level (distances 20, 40 and 60) we collected the OTUs that at least agglomerated 10 ASVs, meaning 488, 466 and 401 OTUs, respectively. For each taxonomic rank, we then calculated proportions of OTUs that had:

1) >50% of ASVs have taxonomic annotation, and majority of these sequences have same annotation (**coded as *50***; indicating *unambiguous* annotation of the OTU),
2) >50% of ASVs have taxonomic annotation, with second most common annotation comprising >30% of ASVs (**coded as *30***; indicating *ambiguous* annotation of the OTU by combining two clades),
3) >50% of ASVs have taxonomic annotation, but the most common annotation is assigned for <50% of ASVs and the second most common annotation is assigned for <30% of ASVs (**coded as *00***; indicating *ambiguous* annotation of the OTU by not representing clearly any clade), and
4) <50% of ASVs have taxonomic annotation (**coded as *NA***; indicating missing annotation for the OTU).

For the second approach, at each taxonomic rank (from domain to genus for RDP, and from domain to species for SILVA) we collected the taxonomic annotations that were annotated to at least 10 ASVs (Table 1). For each clustering level, we then calculated proportions of the clades, that had:

1) >50% of annotations are assigned for same OTU (i.e. for ASVs of same cluster; **coded as *50***; indicating a clade that is unambiguously presented by an OTU)
2) >30% of annotations are assigned for one OTU (i.e. for ASVs of same cluster), while >30 % of annotations are assigned to another OTU (**coded as *30***; indicating a clade that is divided into two OTUs)
3) >50% of annotations are assigned for different OTUs (i.e. ASVs with same taxonomic annotation do not clearly belong to any OTU; **coded as *00***; indicating a clade that is not ambiguously presented by any OTU).

Table 1. Number of clades at each taxonomic level that are with sufficient confidence assigned to at least 10 ASVs.

	domain	phylum	class	order	family	genus	species
**RDP**	2	14	31	34	42	21	0
**SILVA**	2	53	113	209	304	319	28

Based on the first approach, OTUs, if having an annotation for >50% of ASVs based on RDP, have always the same annotation for majority of ASVs at all clustering distances (***50*** or ***NA***; Figure 2). Based on the first approach and SILVA annotations, majority of ASVs within OTUs at all distances had non-NA annotation until family level (***50*** vs. ***NA***; Figure 3). Proportion of OTUs indicating to contain two clades (i.e. ***30***) decrease from phylum- to genus-level (species-level annotations do not have enough data to estimate this) at all clustering distances, yet at the genus level, this proportion is lowest at clustering distance of 20. Proportion of ambiguously ('00') annotated OTUs increase from domain- to family-level, and then decrease to genus-level at all clustering distances.

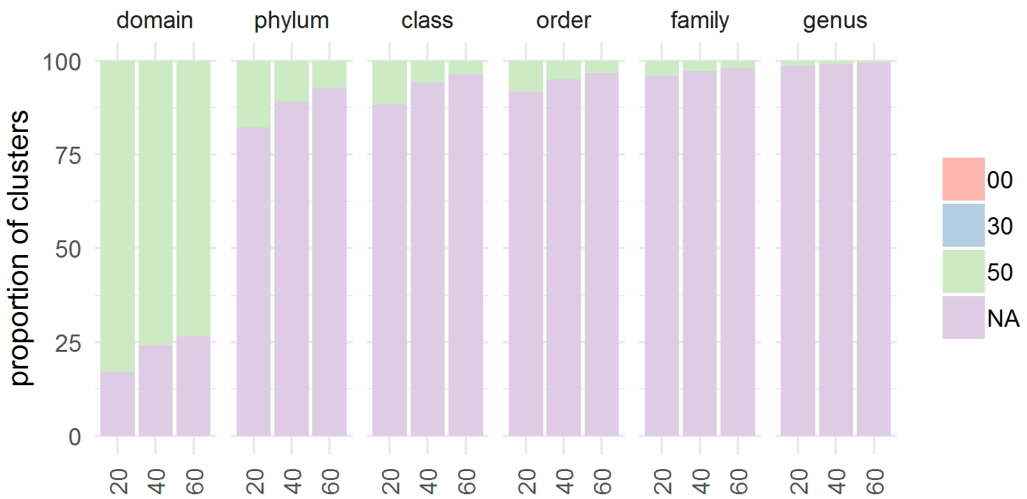

*Figure 2. Proportion of OTUs (i.e. clusters) having unambiguous taxonomic annotation (**50**), ambiguous annotation (**30**=two clades and **00**=no clear annotation) or missing annotation (**NA**) based on RDP assignments.*

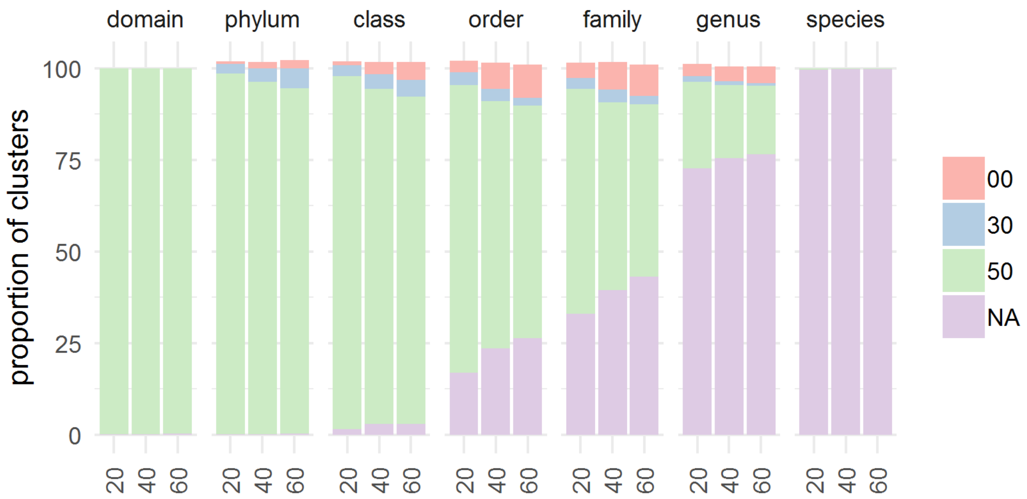

*Figure 3. Proportion of OTUs (i.e. clusters) having unambiguous taxonomic annotation (**50**), ambiguous annotation (**30**=two clades and **00**=no clear annotation) or missing annotation (**NA**) based on SILVA assignments. The part of the bar exceeding 100% indicates the proportion of OTUs that had one annotation for >50% of sequences and another annotation for >30% of sequences.*

Based on the second approach, and RDP and SILVA assignments, from domain- to genus-/species-level, annotations of clades are more-and-more unambiguously assigned to one OTU (Figures 4 and 5). Based on RDP, especially at clustering distance 40, most genus-level annotations are assigned to single OTUs, whereas clustering at distance 60 indicates that some OTUs should be further split and clustering at distance 20 indicate that some genera are split to different clusters. Based on SILVA, most ASVs with same species annotation belong to one OTU at all clustering distances, with highest proportion at distance 60.

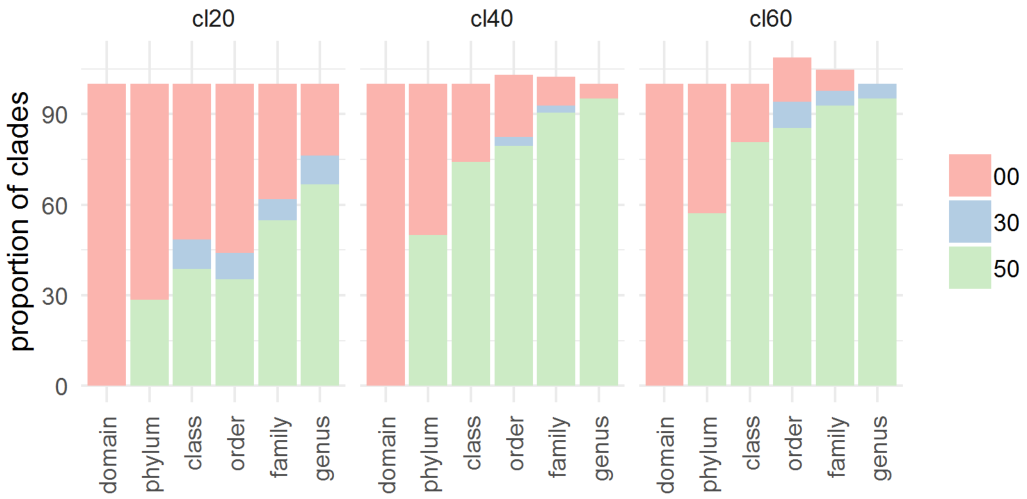

*Figure 4. Proportion of clades, at different clustering distances, that are unambiguously assigned for only one OTU (**50**) or ambiguously assigned (**30**= for two OTUs and **00**= for many OTUs) based on RDP based annotations. The part of the bar exceeding 100% indicates the proportion of clades that had >50% of annotations in one cluster and >30% of annotations in another cluster.*

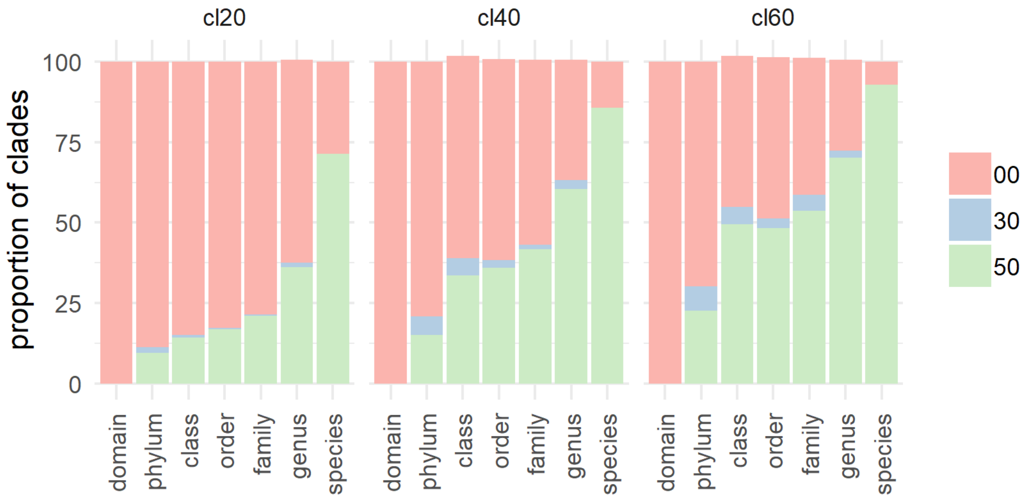

*Figure 5. Proportion of clades, at different clustering distances, that are unambiguously assigned for only one OTU (**50**) or ambiguously assigned (**30**= for two OTUs and **00**= for many OTUs) based on SILVA based annotations. The part of the bar exceeding 100% indicates the proportion of clades that had >50% of annotations in one cluster and >30% of annotations in another cluster.*

The two approaches and the two assignments (RDP and SILVA) do not completely agree, likely due to the varying numbers of OTUs/clades with enough confidence between RDP and SILVA. Nevertheless, compromising among the two approaches and RDP and SILVA, we conclude the clustering distances represented species/genus. Based on the Figure 5, clustering at distance 20 might even go beyond species level, while based on the Figure 4 some OTUs clustered at distance 60 combine two genera. Thus, in the main manuscript, we base the results in clustering distance 40 as a compromise among the different clustering distances, and likely representing species. Finally, it seems, that different clustering distances cannot be unambiguously said to represent certain taxonomic ranks. Reasons for this are multitude, in addition to clustering approach, quality and length of sequences and unambiguousness of the DNA reference database.

References:

Wang, Q., G. M. Garrity, J. M. Tiedje, and J. R. Cole. 2007. Naïve Bayesian Classifier for Rapid Assignment of rRNA Sequences into the New Bacterial Taxonomy. Applied and Environmental Microbiology **73**:5261-5267.

Yilmaz, P., L. W. Parfrey, P. Yarza, J. Gerken, E. Pruesse, C. Quast, T. Schweer, J. Peplies, W. Ludwig, and F. O. Glöckner. 2014. The SILVA and "All-species Living Tree Project (LTP)" taxonomic frameworks. Nucleic Acids Research **42**:D643-D648.
