# Supplementary material for: Predicting spatial patterns of soil bacteria under current and future environmental conditions
Source: ISME J. 2021 Mar 12;15(9):2547–60. doi: 10.1038/s41396-021-00947-5 (PMC8397778; doi:10.1038/s41396-021-00947-5)
Supplement: Supplementary file 2 — Appendix 2 [file 41396_2021_947_MOESM2_ESM.pdf]

Supplementary material to Mod et al. *Predicting spatial patterns of soil bacteria under current and future environmental conditions.* ISME j.

Appendix 2: Environmental data

For the study area, there were 79 environmental variables available (unil.ch/centre-montagne) comprising

- 26 climatic factors (average and extreme conditions and seasonality based on normal period 1981-2010; meteoswiss.ch; original 1 km resolutions data downscaled to 25 m using local linear regressions with elevation in a moving window of 5 km radius; CHclim25; https://www.unil.ch/ecospat/en/home/menuguid/ecospat-resources/data.html),
- five topographic factors (elevation, and debris, light, moisture accumulation / deficiency; swisstopo.ch; derived from 25 m digital elevation model), and
- 39 edaphic factors (physical and chemical conditions including e.g. soil pH and nutrient availability). The edaphic variables were measured in the field during metagenomics soil sampling or analysed in laboratory from the soil samples using both routine and advanced techniques (for more details, see Yashiro et al. 2016 and , Buri 2019).

For the variables with strongly right-skewed distribution, we added also a version with logarithmic transformation. To choose the final set of predictors for the models (see Appendix 3), we first built generalized additive models (GAM, with negative binomial family and spline smoothers from mgcv R-package) for each OTU with each of the 79 variables (+logarithms) as a predictor at time. We then chose three climatic, topographic and edaphic predictors compromising among biological significance, mean R^2 of the GAMs across OTUs and multicollinearity of the variables.

The non-correlating variables with the highest mean R^2 among all bacteria datasets were soil pH, freezing degree days (FDD; sum of temperatures from days with mean T < 0°C), logarithm of total organic carbon (TOC_{log}) and annual temperature range (T_{range}). For the three climatic variables we chose variables that represent seasonality, temperature and precipitation. T_{range} was chosen for seasonality, but instead of FDD as the temperature variable, we chose temperature of the coldest quarter (T_{coldQ}), as under the future climate scenario, FDD reaches zero, and does not reflect climatic warming beyond this maximum value. For precipitation, we chose the variable with the highest mean R^2: precipitation of the driest month (P_{dryM}). Climatic conditions strongly follow elevation in our study area, and thus correlation among variables representing temperature and precipitation is unavoidable. Thus, despite the correlation of -0.87 between T_{coldQ} and P_{dryM}, we chose to keep both, as in the future scenarios of temperature and precipitation their magnitudes of changes vary (Figures 1-2). The three chosen topographic variables were potential annual solar radiation (sRad), topographic position index (TPI) and slope angle. Finally, the edaphic variables include, in addition to soil pH and TOC_{log}, percentage of clay particles, representing soil grain size. Among the variables, apart from T_{coldQ} and P_{sumM}, the absolute correlations varied between 0.02 and 0.66.

For the future projections we used an independent dataset of 229 sites. Future climatic variables for these sites were derived from climate change scenario data and scenarios for future soil pH and TOC were estimated based on soil resurvey data (Figures 3-4).

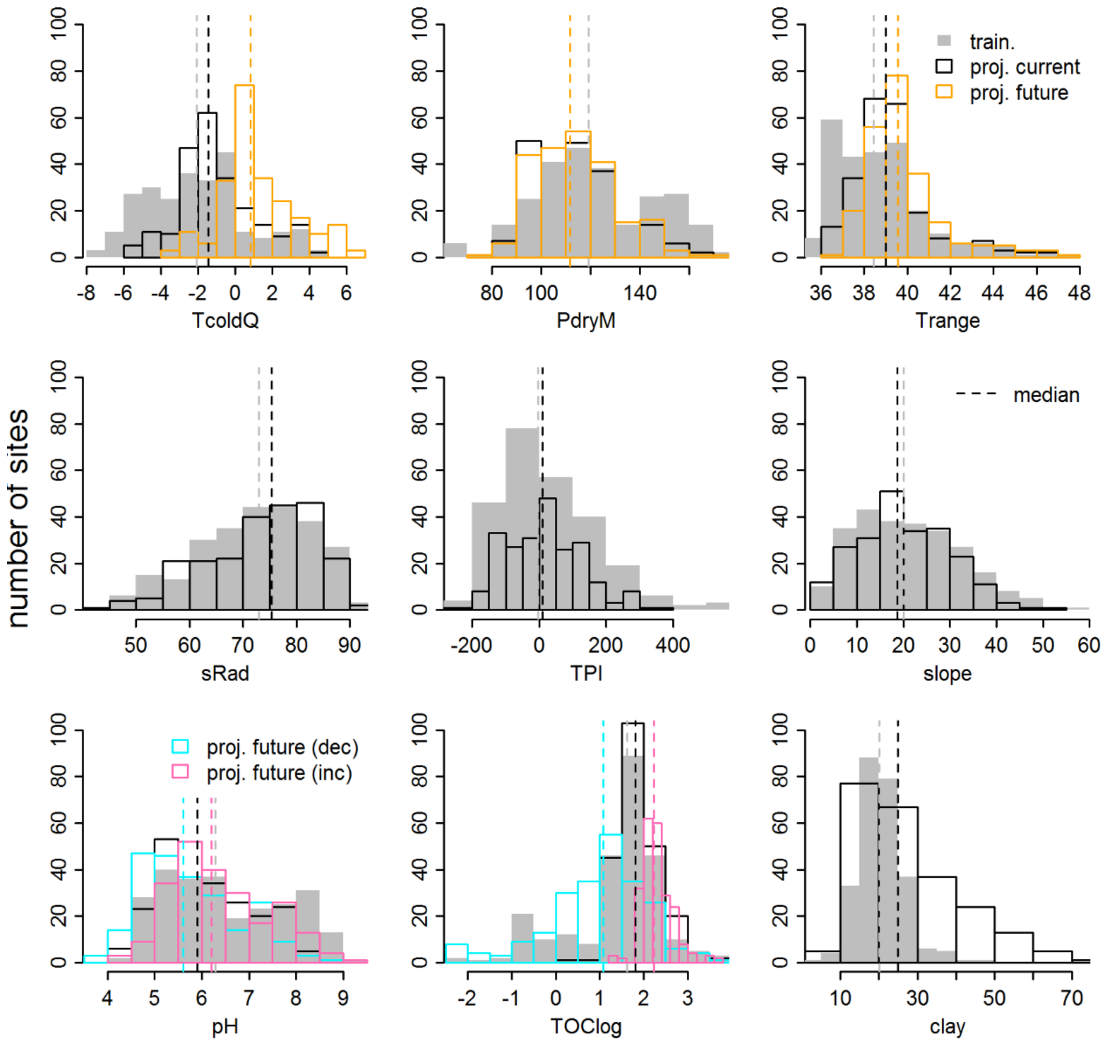

Figure 1. Distributions of environmental variables among the dataset with 255 sites used to train the models and among the datasets with 229 sites, under current conditions and future scenarios, used for projections.

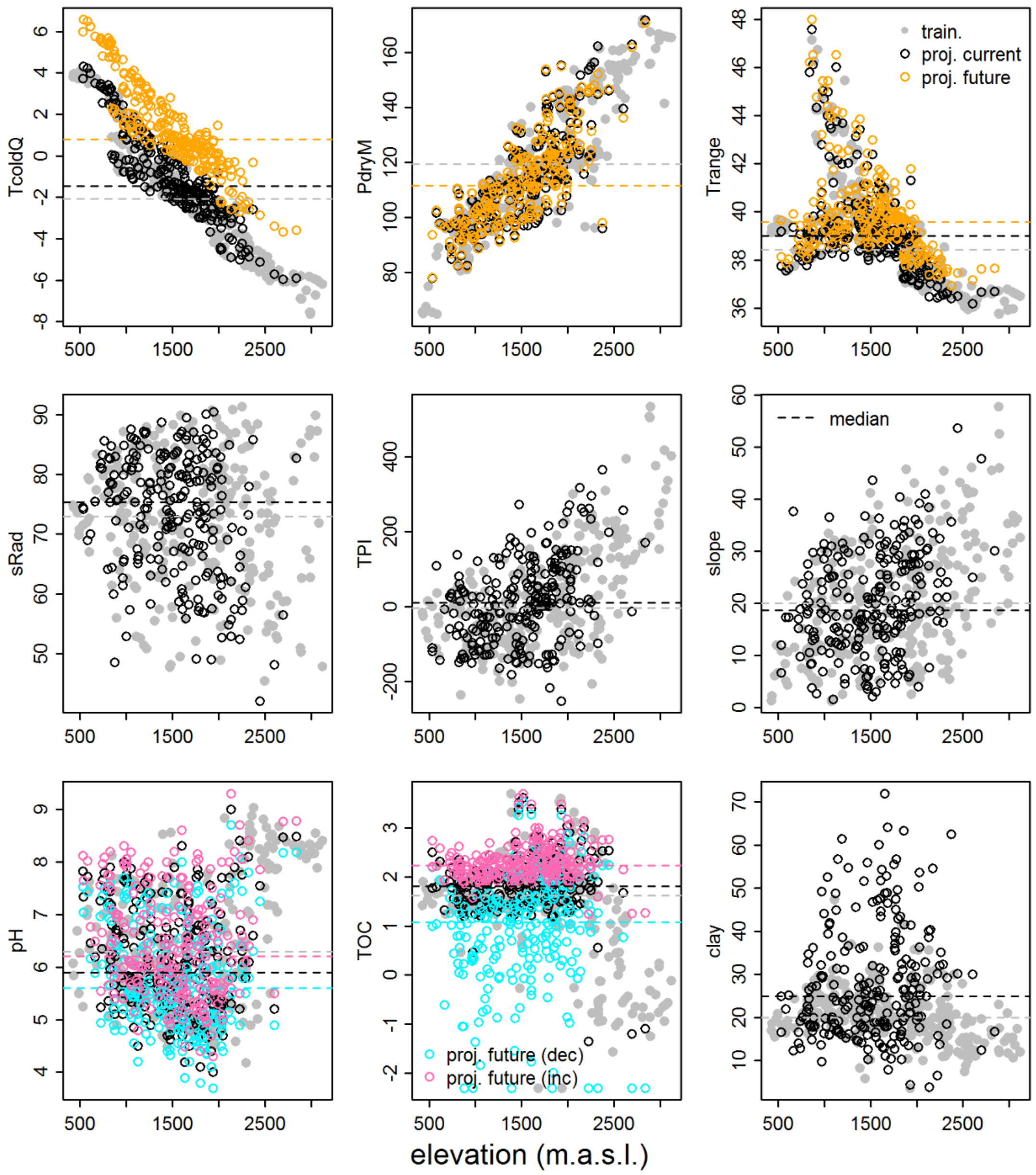

Figure 2. Relationships of environmental variables to elevation in different dataset (train = 255 sites used to train the models, proj = 229 sites, under current conditions and future scenarios, used for projections).

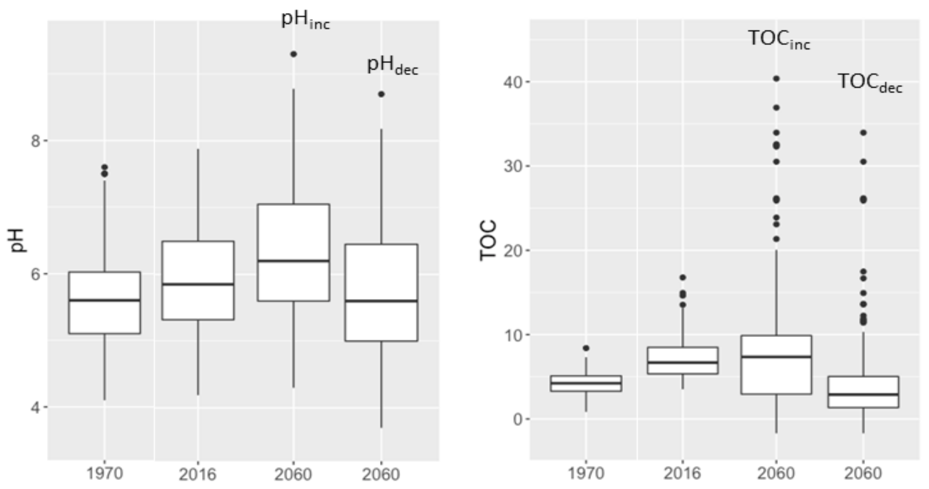

Figure 3. Observed pH values and total organic contents (%, TOC) in the 112 paired soil samples from 1970 and 2016, and scenarios of pH and TOC for year 2060 based on the observed changes between 1970 and 2016. pH_{inc} and TOC_{inc} assume that observed increases in pH and TOC continue, whereas pH_{dec} and TOC_{dec} assume the same amount of decrease in pH and TOC.

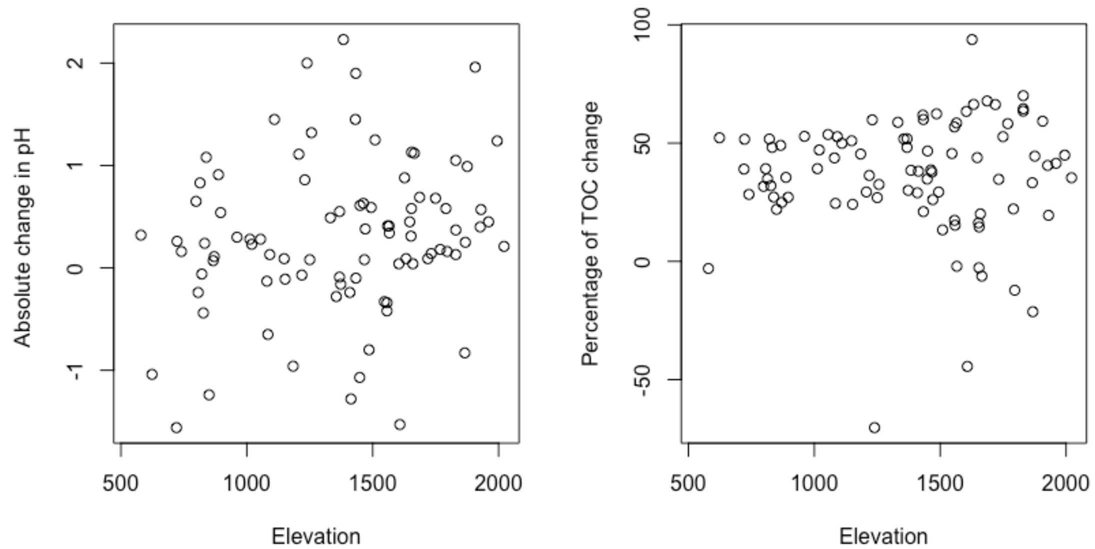

Figure 4. Observed changes in soil pH and total organic content (%, TOC) in the 112 paired soil samples from 1970 and 2016 in relation to elevation. The correlation between absolute change in pH and elevation is 0.2156217; p = 0.044, and between percentage change in TOC and elevation 0.03425381; p = 0.751. Elevation explains 2.8 % and 0 % of change of pH and TOC, respectively.

References:

Buri, A. 2019. Above- and belowground biogeography : Spatial modelling of a hidden system. . University of Lausanne, Lausanne.

Yashiro, E., E. Pinto-Figueroa, A. Buri, J. E. Spangenberg, T. Adatte, H. Niculita-Hirzel, A. Guisan, and J. R. van der Meer. 2016. Local Environmental Factors Drive Divergent Grassland Soil Bacterial Communities in the Western Swiss Alps. Applied and Environmental Microbiology **82**:6303-6316.
