# Supplementary material for: Predicting spatial patterns of soil bacteria under current and future environmental conditions
Source: ISME J. 2021 Mar 12;15(9):2547–60. doi: 10.1038/s41396-021-00947-5 (PMC8397778; doi:10.1038/s41396-021-00947-5)
Supplement: Supplementary file 3 — Appendix 3 [file 41396_2021_947_MOESM3_ESM.pdf]

Supplementary material to Mod et al. *Predicting spatial patterns of soil bacteria under current and future environmental conditions.* ISME j.

Appendix 3: Analytical framework adapted from a framework designed to quantify niche of species by modelling occurrence (based on presences (and absences)). Here, the framework is adjusted to model and predict sequence counts of bacterial OTUs**.**

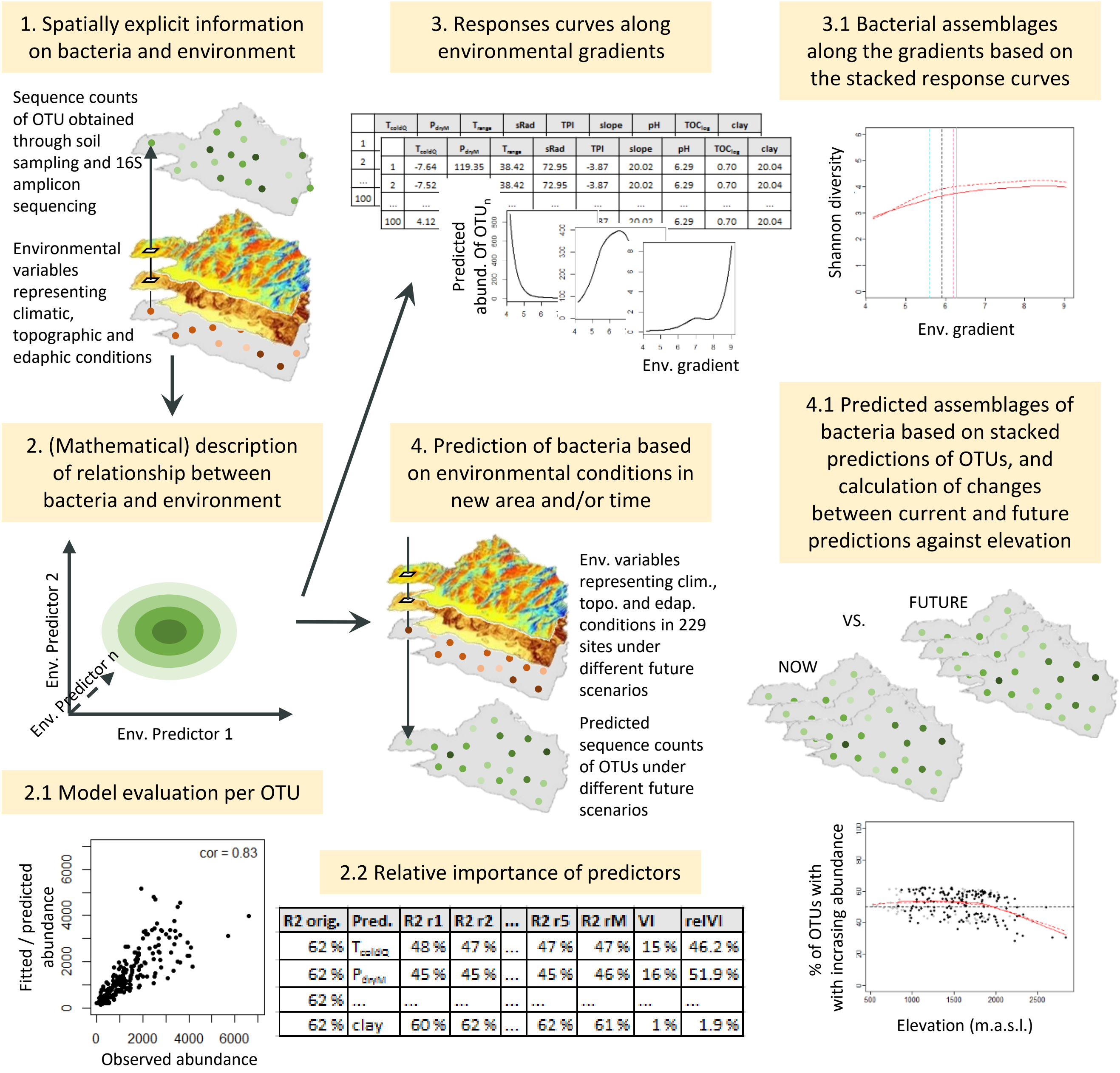

R2 orig.	Pred.	R2 r1	R2 r2	...	R2 r5	R2 rM	VI	relVI
62 %	T_{coldQ}	48 %	47 %	...	47 %	47 %	15 %	46.2 %
62 %	P_{dryM}	45 %	45 %	...	45 %	46 %	16 %	51.9 %
62 %	...	...	...	...	...	...	...	...
62 %	clay	60 %	62 %	...	62 %	61 %	1 %	1.9 %

1. Abundance (sequence counts; see in the main text) of OTU from 255 sites are combined with nine environmental predictors derived from climate data, digital elevation model and soils samples (see Appendix 2).
2. Using mathematical algorithms (here GAMnb, GAMp and GBM), the relationship between abundance of OTU and environmental variables are quantified (for details see main text). 2.1 Model performance was assessed by comparing observed abundance to fitted and predicted (see main text) abundance. 2.2 With GAM, the importance of predictors was calculated as the decrease of R^2 (mean of five repetition) between the original model and the model with the predictor of interest being shuffled. Final variable importance values of all nine predictors per OTU are proportional to the total sum of R^2-decreases. For GBM, see the default inbuilt function in the package *gbm*.
3. Abundance of OTU is predicted against "environmental space" where one environmental predictor varies from min to max while other predictors are fixed to median conditions. 3.1 Based on the response curves of all OTUs (with sufficient model performance), i) % of OTUs with abundance > median, ii) Shannon index, and iii) relative abundance of phyla are calculated along the environmental gradients.
4. Abundances of OTUs are predicted in 229 projections sites under the current environmental conditions and nine combinations of future scenarios. 4.1 Based on the predicted abundances of all OTUs (with sufficient model performance), i) % of OTUs with increase/decrease in abundance, ii) change in Shannon index, and iii) relative abundance of phyla are calculated, and shown against elevation.
