# Supplementary material for: Predicting spatial patterns of soil bacteria under current and future environmental conditions
Source: ISME J. 2021 Mar 12;15(9):2547–60. doi: 10.1038/s41396-021-00947-5 (PMC8397778; doi:10.1038/s41396-021-00947-5)
Supplement: Supplementary file 4 — Appendix 4 [file 41396_2021_947_MOESM4_ESM.pdf]

# Appendix 4: Additional results

*Table S1. Library sizes of 255 sites following de novo approach.*

| Min | 1st quartile | Median | Mean | 3rd quartile | Max |
|---|---|---|---|---|---|
| 143 438 | 859 814 | 1 491 953 | 1 576 920 | 1 998 237 | 6 366 402 |

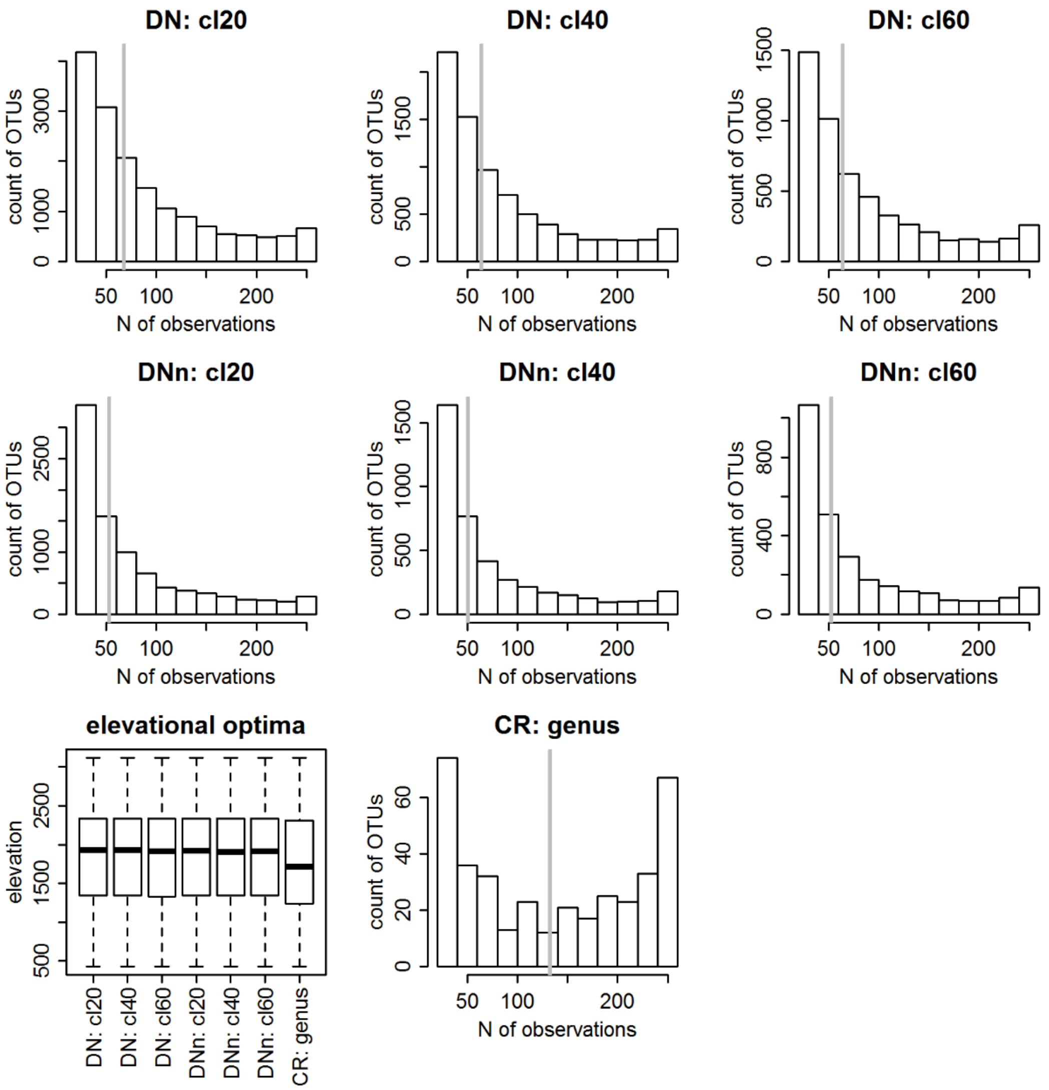


*Figure S1. Number of occurrence observations and elevational optima of modelled OTUs and genera in different datasets. Grey vertical lines indicate median number of occurrences.*

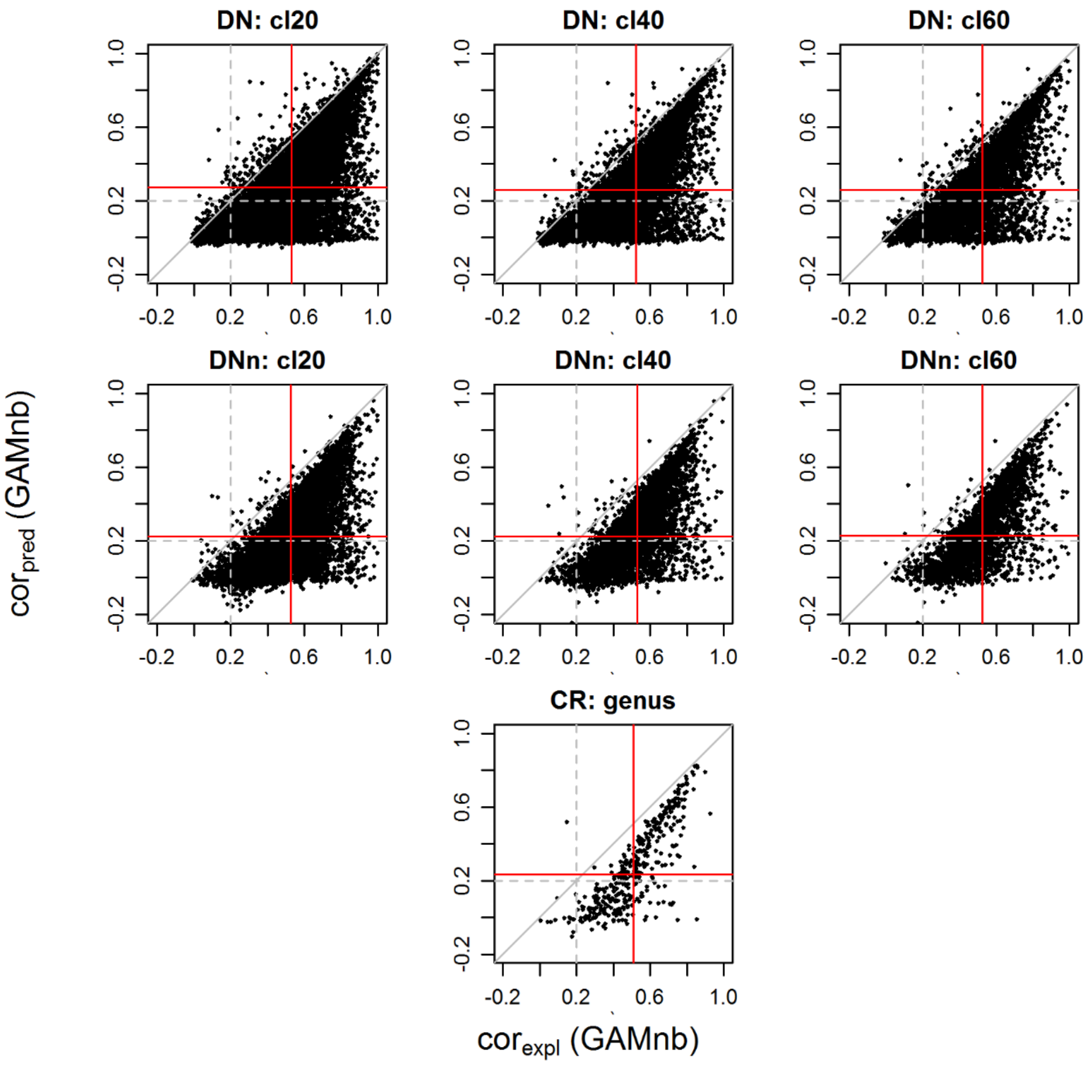


*Figure S2. Relationship of $cor_{expl}$ (correlation of observed and fitted abundances per OTU) and $cor_{pred}$ (correlations of observed and predicted abundances with cross-validation) based on* ***GAMnb****. Red lines indicate median correlations and dashed grey lines indicate thresholds for excluding OTUs with low model performance from projections.*

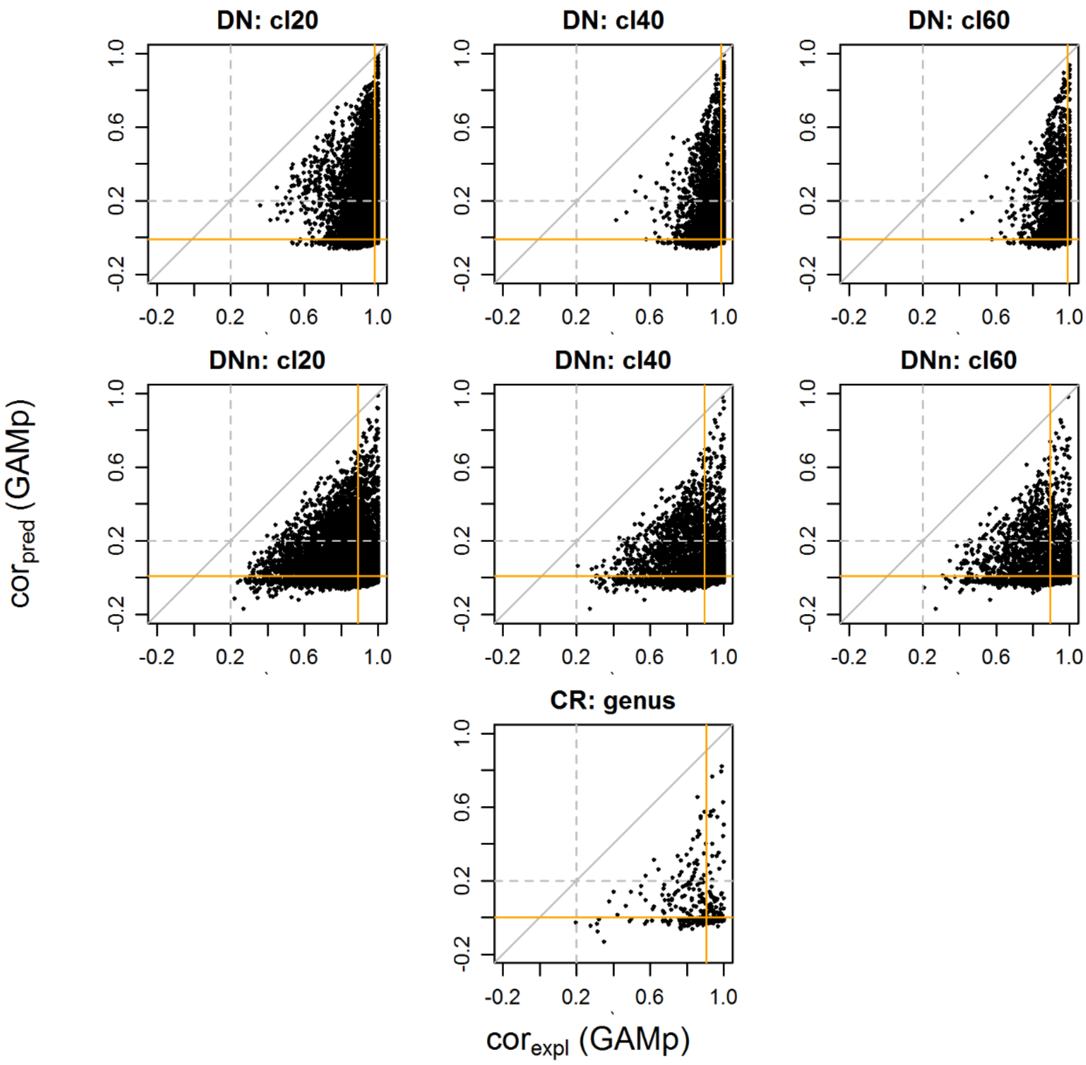


*Figure S3. Relationship of cor$_{expl}$ (correlation of observed and fitted abundances per OTU) and cor$_{pred}$ (correlations of observed and predicted abundances with cross-validation) based on* ***GAMp****. Orange lines indicate median correlations and dashed grey lines indicate thresholds for excluding OTUs with low model performance from projections.*

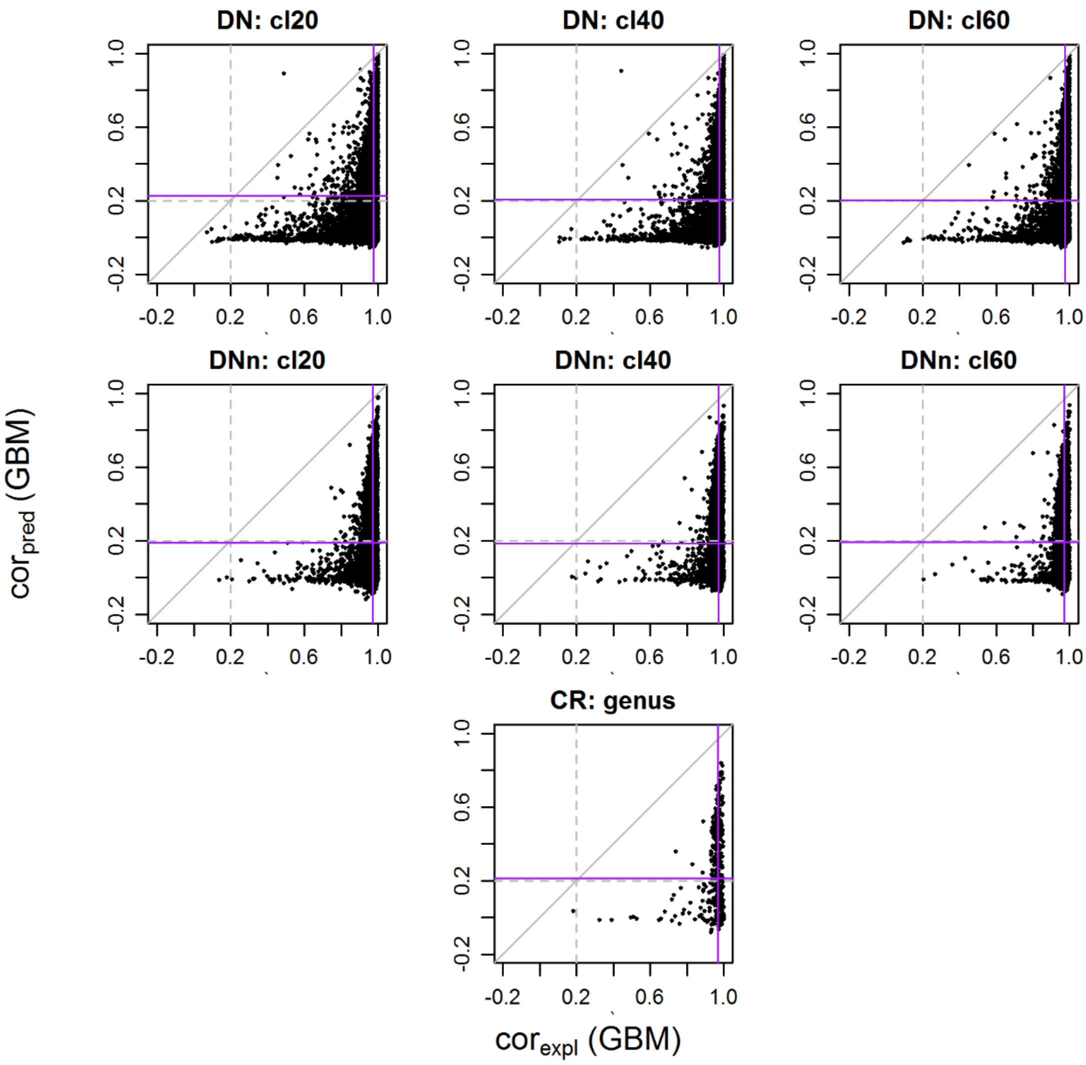


*Figure S4. Relationship of $cor_{expl}$ (correlation of observed and fitted abundances per OTU) and $cor_{pred}$ (correlations of observed and predicted abundances with cross-validation) based on* ***GBM****. Purple lines indicate median correlations and dashed grey lines indicate thresholds for excluding OTUs with low model performance from projections.*

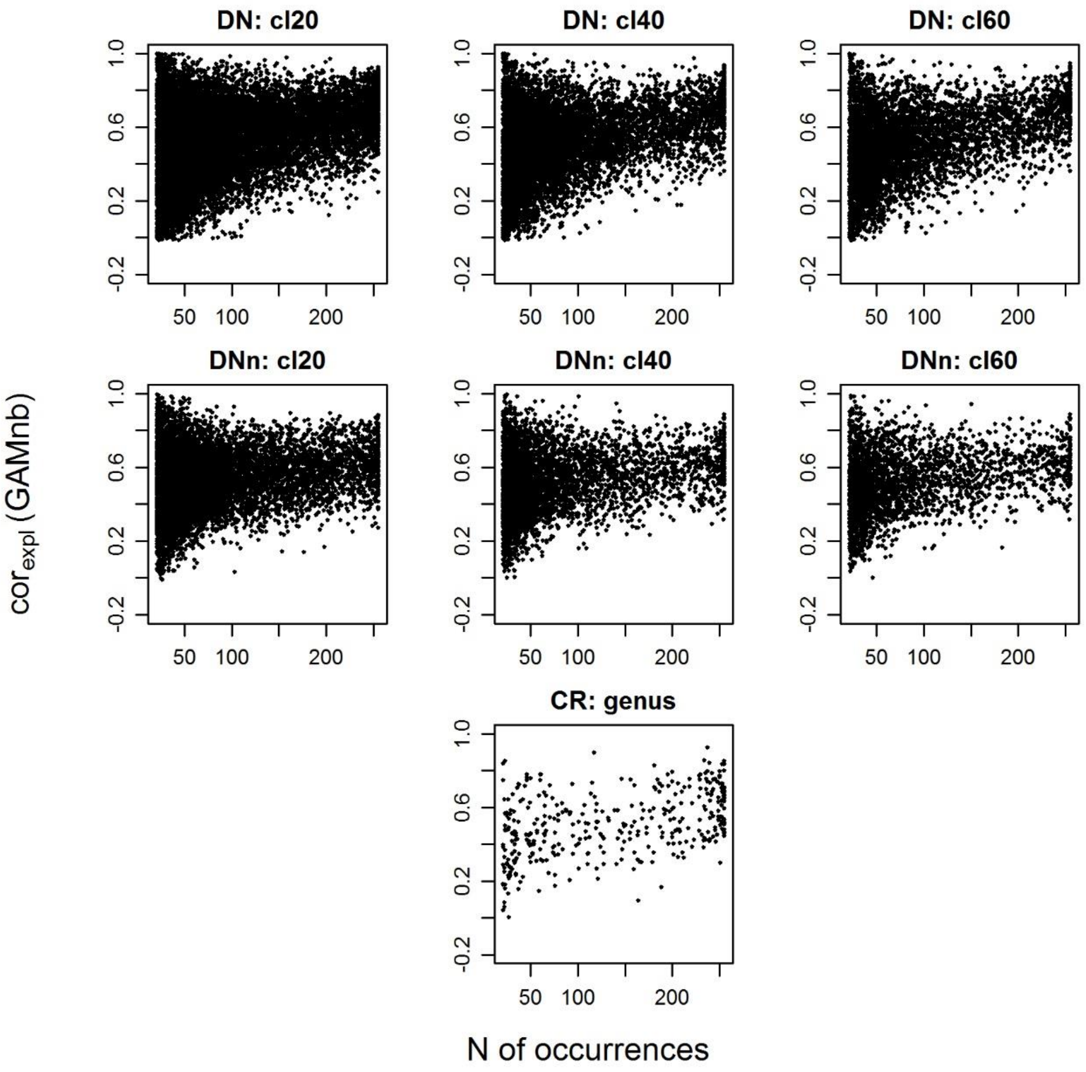


*Figure S5. Relationship of $cor_{expl}$ (correlation of observed and fitted abundances per OTU) and number of occurrences (i.e. non-zero abundance) per OTU based on **GAMnb**.*

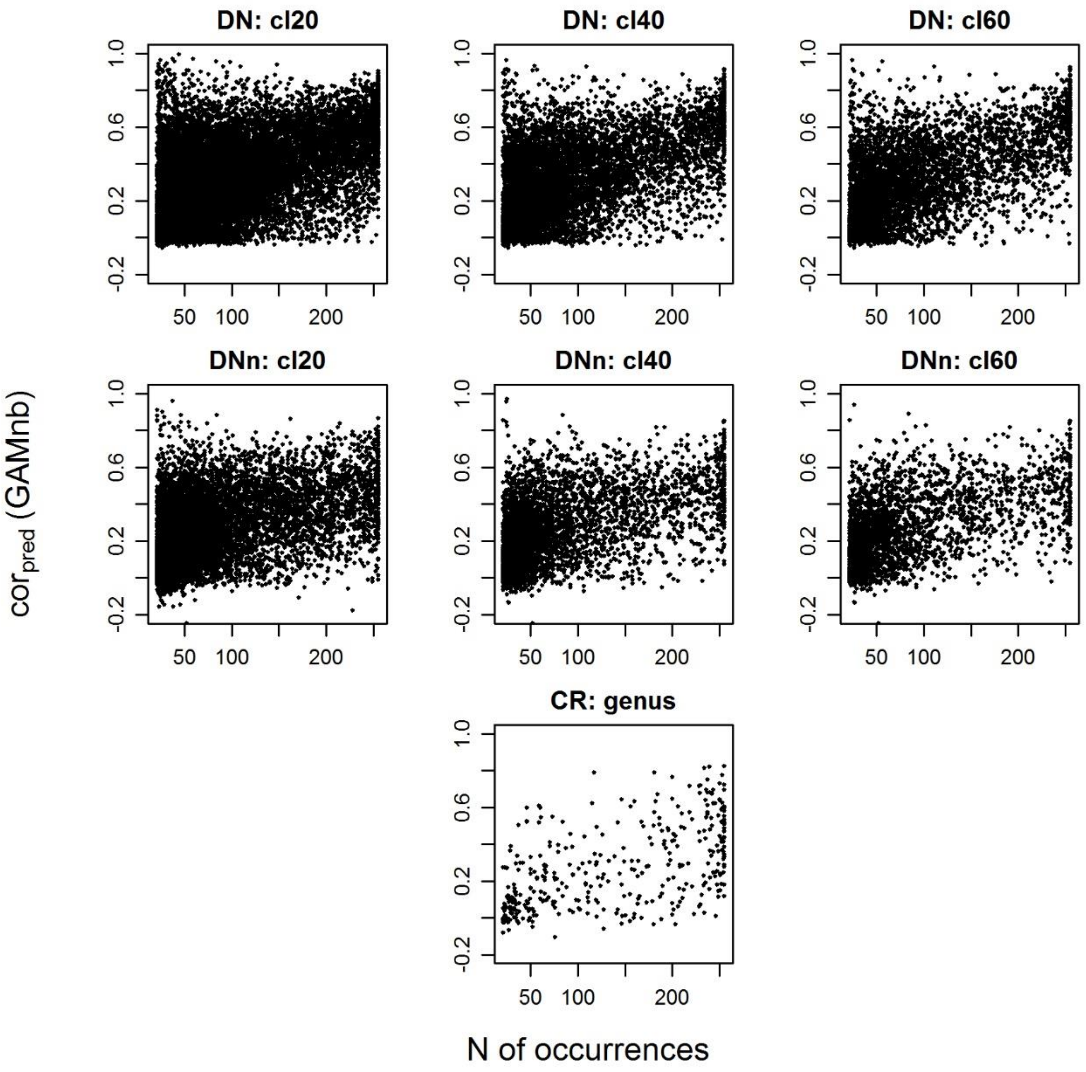


*Figure S6. Relationship of $cor_{pred}$ (correlations of observed and predicted abundances with cross-validation) and number of occurrences (i.e. non-zero abundance) per OTU based on **GAMnb**.*

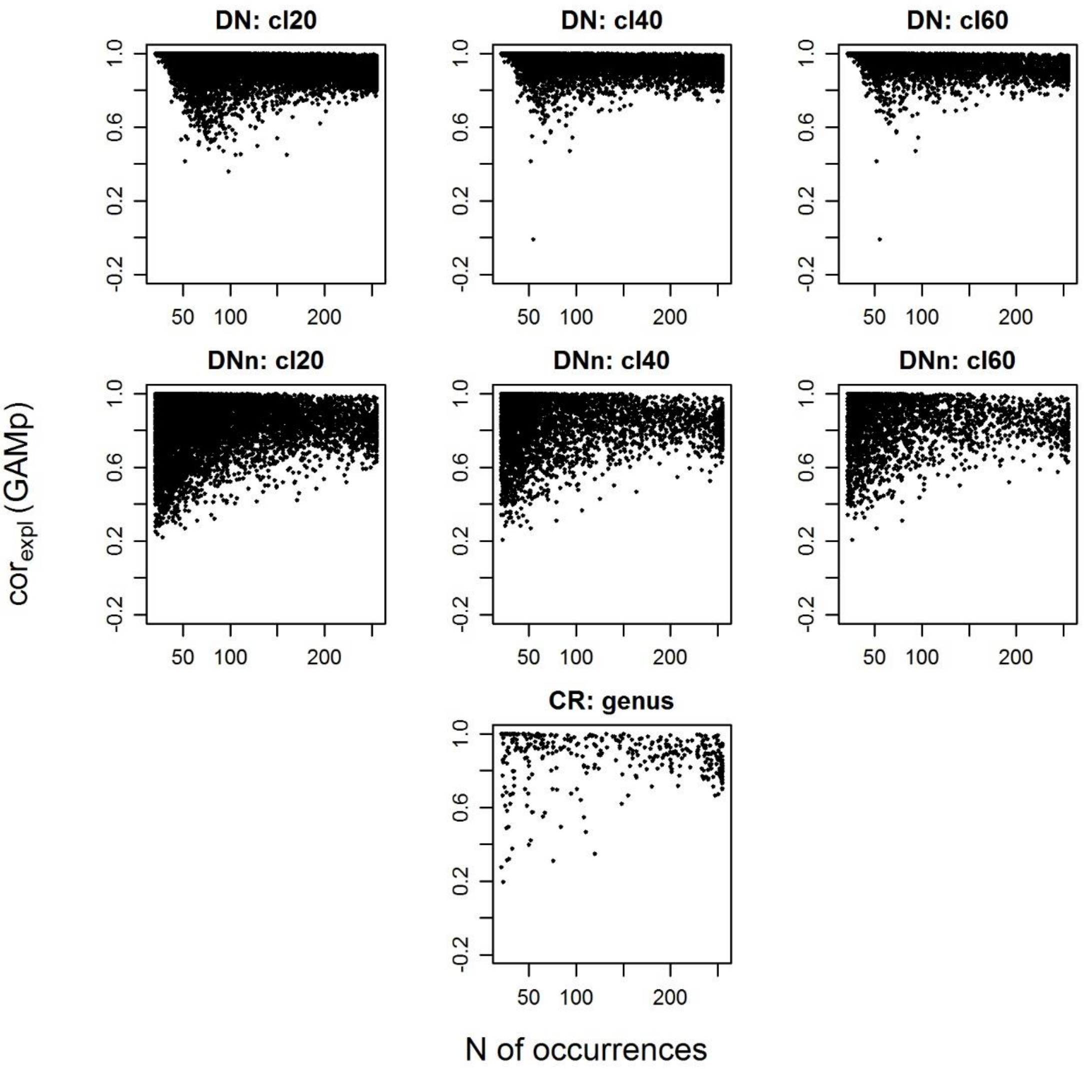


*Figure S7. Relationship of $cor_{expl}$ (correlation of observed and fitted abundances per OTU) and number of occurrences (i.e. non-zero abundance) per OTU based on **GAMp**.*

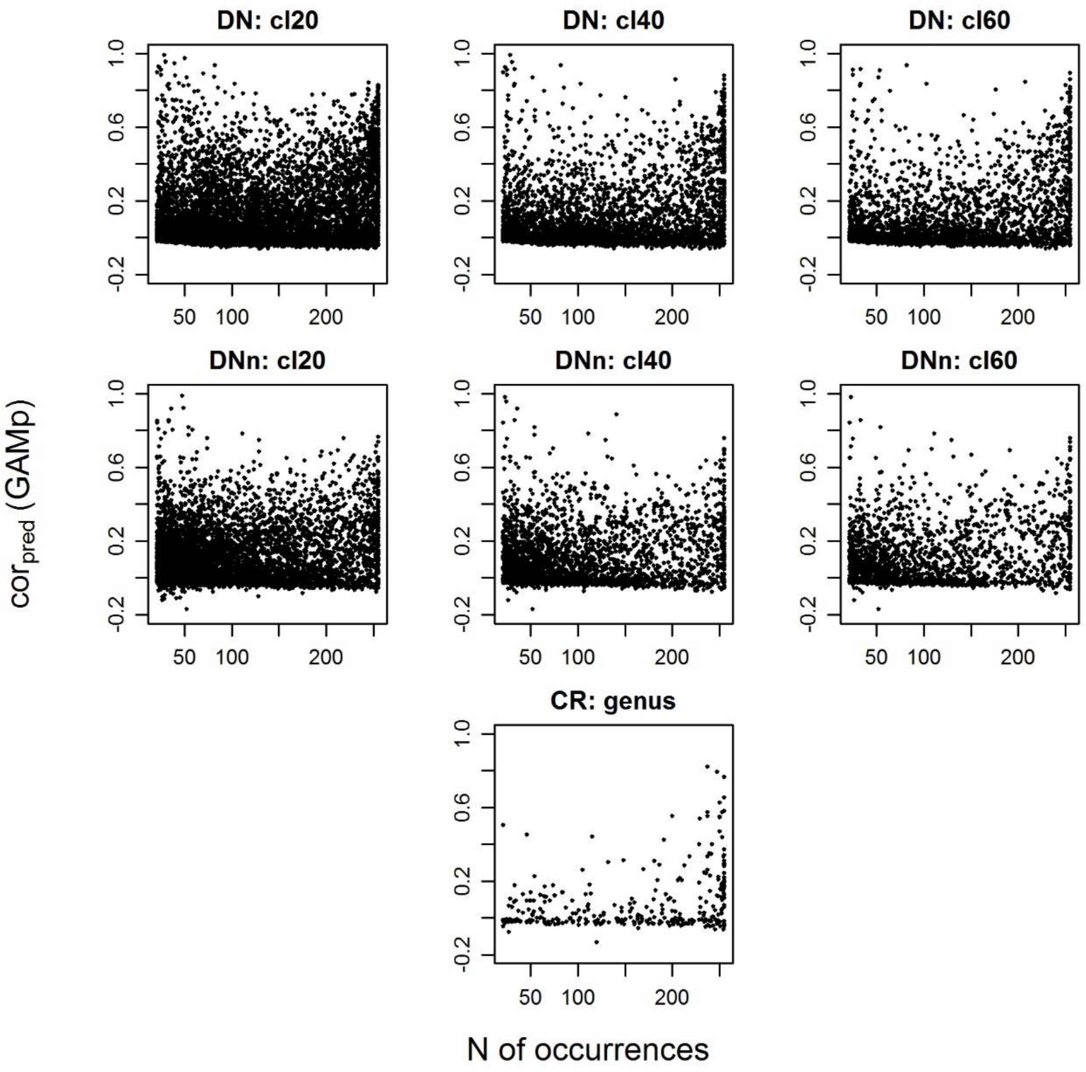


*Figure S8. Relationship of cor$_{pred}$ (correlations of observed and predicted abundances with cross-validation) and number of occurrences(i.e. non-zero abundance) per OTU based on* ***GAMp****.*

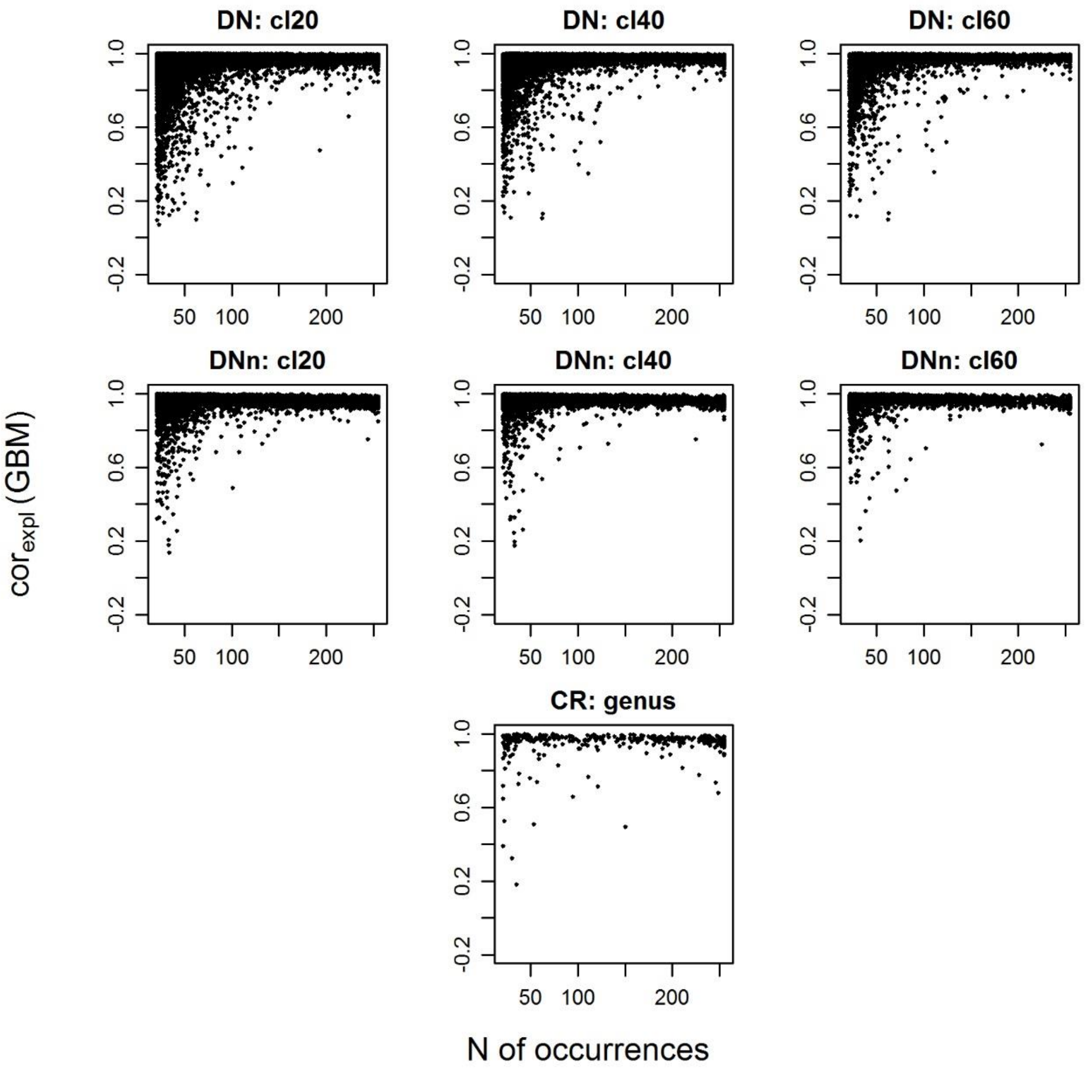


*Figure S9. Relationship of $cor_{expl}$ (correlation of observed and fitted abundances per OTU) and number of occurrences (i.e. non-zero abundance) per OTU based on* ***GBM****.*

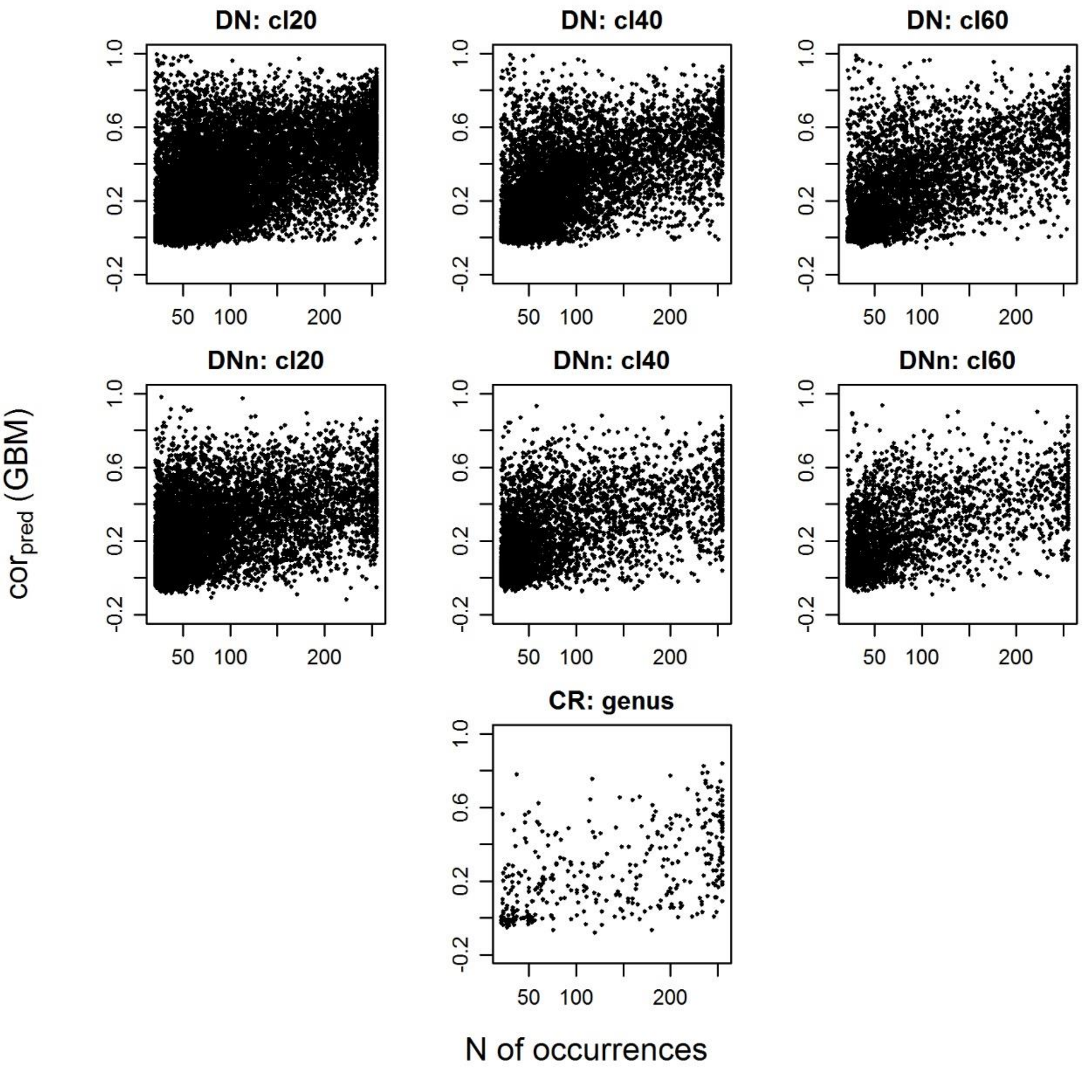


*Figure S10. Relationship of $cor_{pred}$ (correlations of observed and predicted abundances with cross-validation) and number of occurrences (i.e. non-zero abundance) per OTU based on **GBM**.*

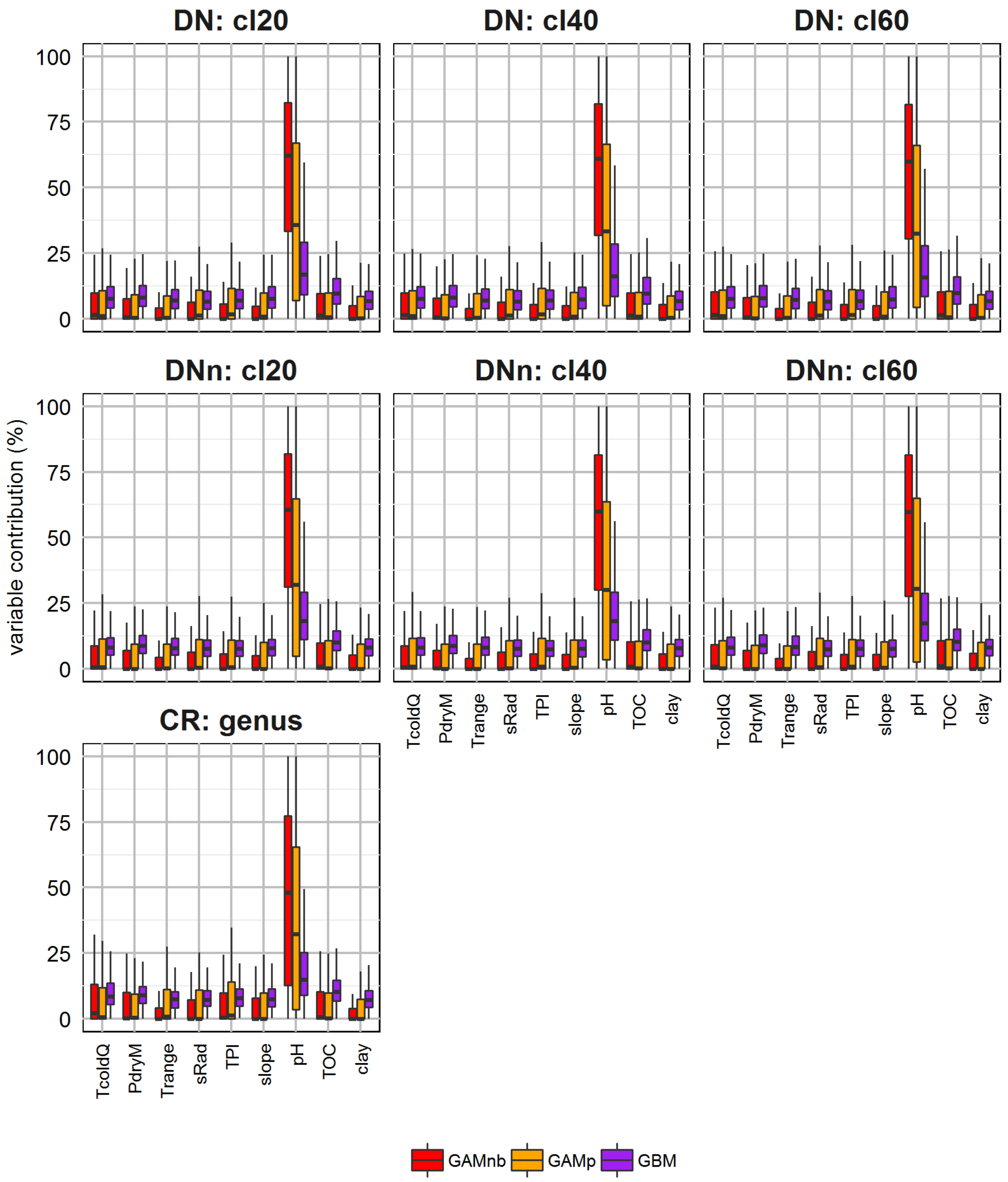


*Figure S11. Variable contributions for each OTU within the different models and datasets.*

***Figures S12-15 (next four pages)***

*Figure S12: Variation of bacterial assemblages along the environmental gradients calculated as percentage of OTUs with abundance > median abundance along the gradient based on OTUs' response curves from **GAMnb** and **GBM**. Vertical lines indicate median environmental conditions among the different datasets: training = the 255 sites used to train models, proj. cur = current conditions in the 229 projection sites, proj. clim = future climatic conditions in the 229 projection sites, proj. dec. = future edaphic conditions in the 229 projection sites under decreasing scenario and proj. inc. = future edaphic conditions in the 229 projection sites under increasing scenario.*

*Figure S13: Variation of bacterial assemblages along the environmental gradients calculated as Shannon diversity based on OTUs' response curves from **GAMnb** and **GBM**. Vertical lines indicate median environmental conditions among the different datasets: training = the 255 sites used to train models, proj. cur = current conditions in the 229 projection sites, proj. clim = future climatic conditions in the 229 projection sites, proj. dec. = future edaphic conditions in the 229 projection sites under decreasing scenario and proj. inc. = future edaphic conditions in the 229 projection sites under increasing scenario.*

*Figure S14: Variation of bacterial assemblages along the environmental gradients expressed as relative abundances of phyla based on OTUs' response curves from **GAMnb**.*

*Figure S15: Variation of bacterial assemblages along the environmental gradients expressed as relative abundances of phyla based on OTUs' response curves from **GBM**.*

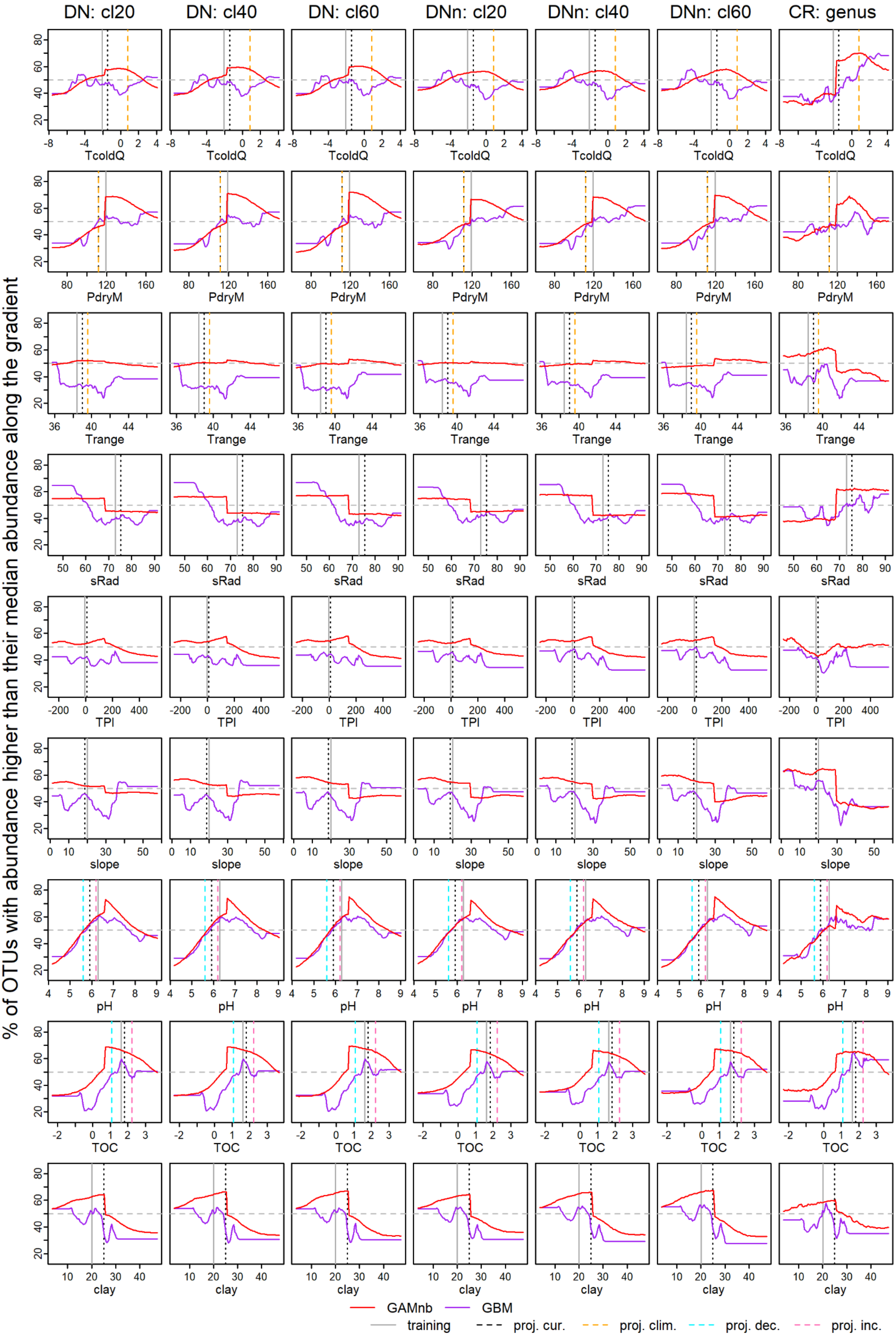

DN: cl20
DN: cl40
DN: cl60
DNn: cl20
DNn: cl40
DNn: cl60
CR: genus
% of OTUs with abundance higher than their median abundance along the gradient
TcoldQ
PdryM
Trange
sRad
TPI
slope
pH
TOC
clay
GAMnb
GBM
training
proj. cur.
proj. clim.
proj. dec.
proj. inc.

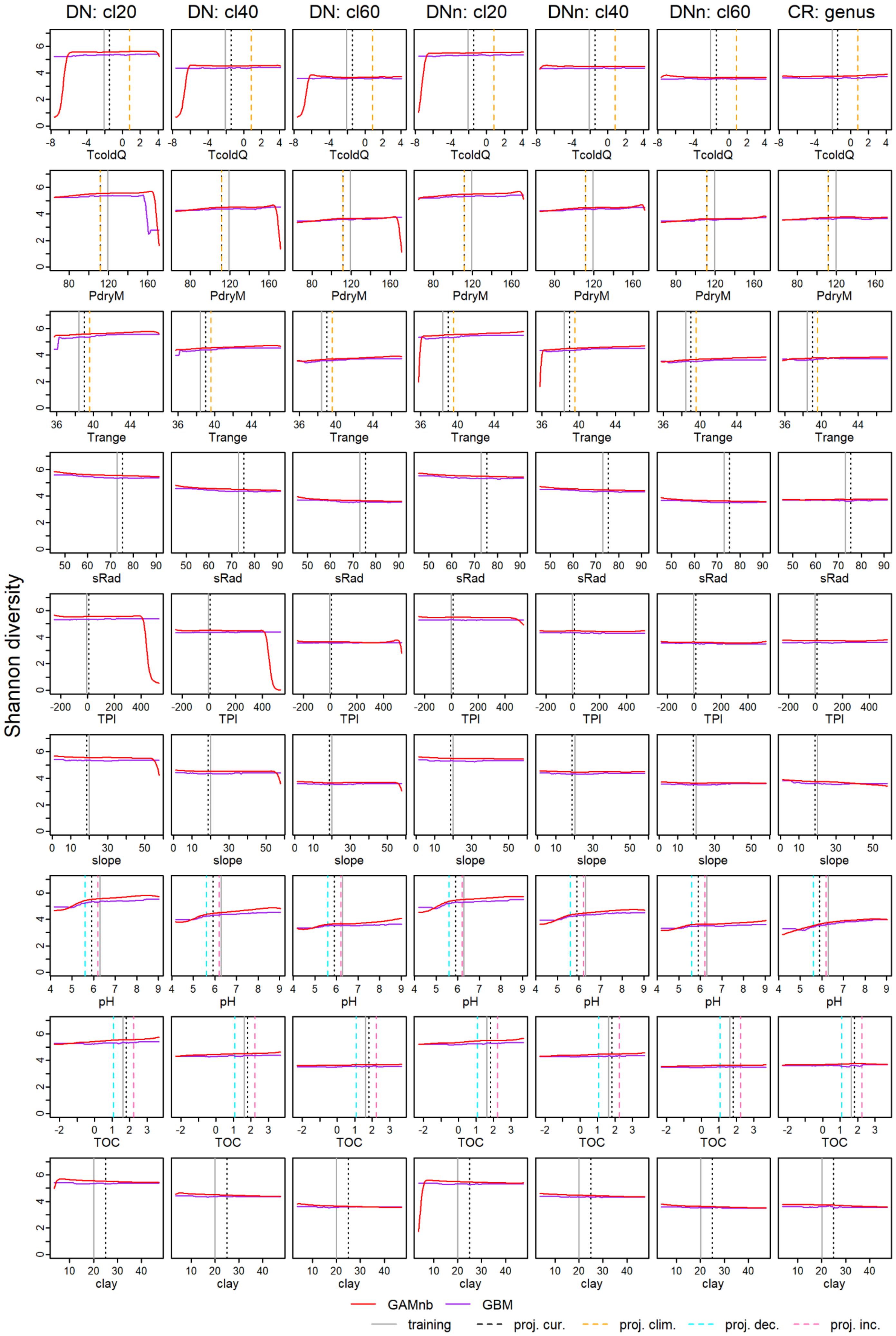
DN: cl20
DN: cl40
DN: cl60
DNn: cl20
DNn: cl40
DNn: cl60
CR: genus
Shannon diversity
TcoldQ
PdryM
Trange
sRad
TPI
slope
pH
TOC
clay
GAMnb
GBM
training
proj. cur.
proj. clim.
proj. dec.
proj. inc.

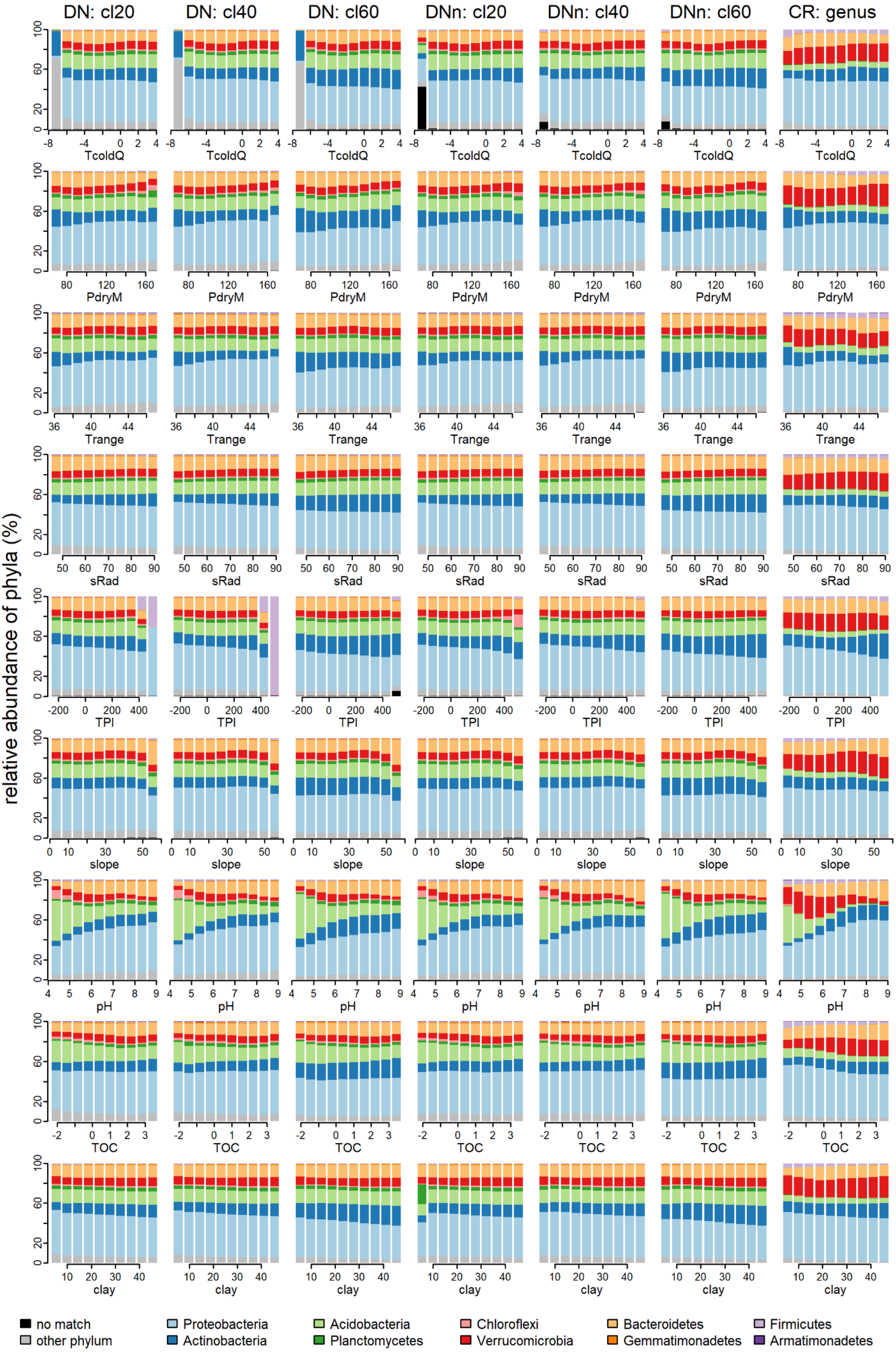

DN: cl20
DN: cl40
DN: cl60
DNn: cl20
DNn: cl40
DNn: cl60
CR: genus
relative abundance of phyla (%)
TcoldQ
PdryM
Trange
sRad
TPI
slope
pH
TOC
clay
no match
other phylum
Proteobacteria
Actinobacteria
Acidobacteria
Planctomycetes
Chloroflexi
Verrucomicrobia
Bacteroidetes
Gemmatimonadetes
Firmicutes
Armatimonadetes

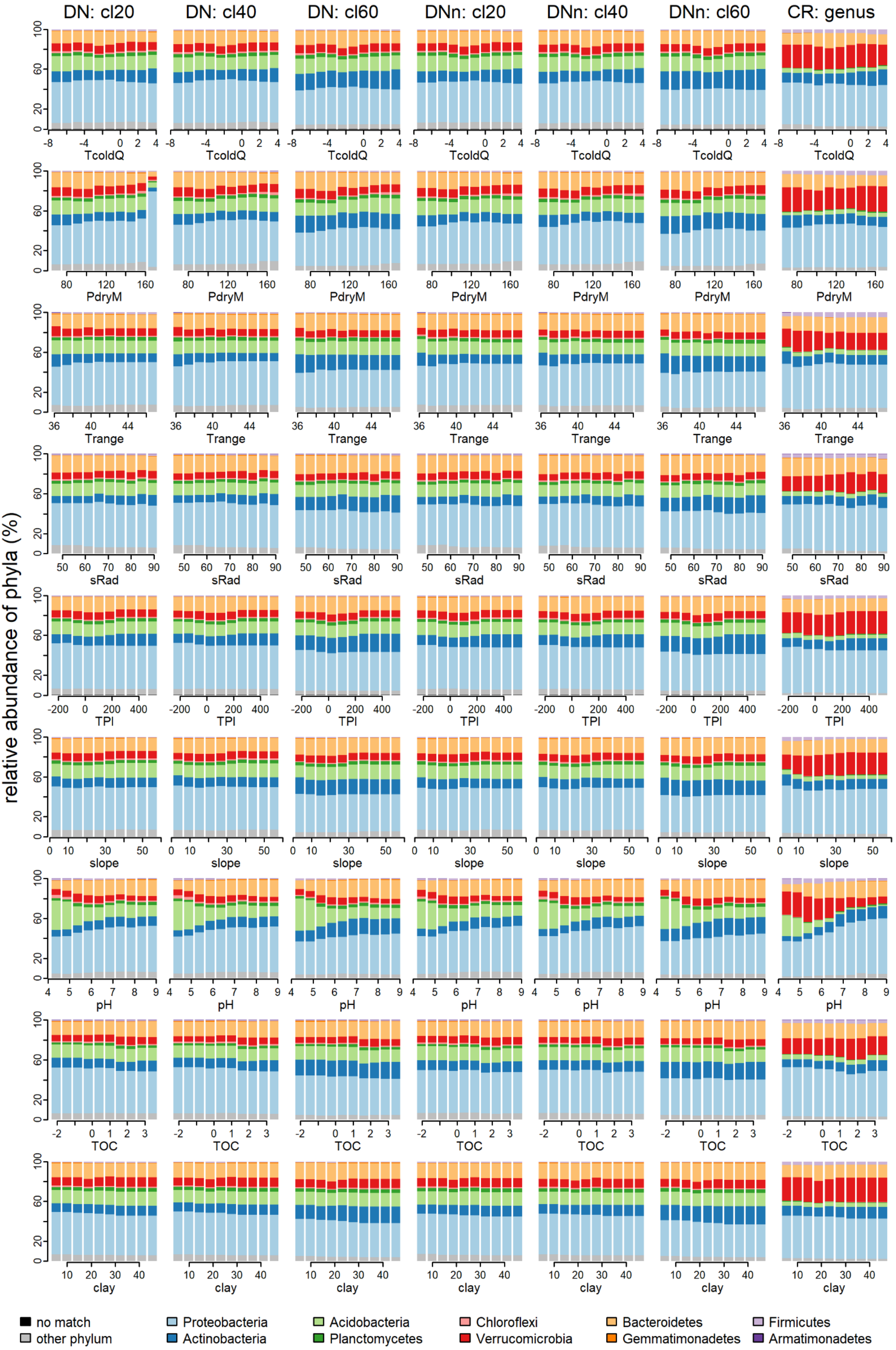

DN: cl20
DN: cl40
DN: cl60
DNn: cl20
DNn: cl40
DNn: cl60
CR: genus
relative abundance of phyla (%)
TcoldQ
PdryM
Trange
sRad
TPI
slope
pH
TOC
clay
no match
other phylum
Proteobacteria
Actinobacteria
Acidobacteria
Planctomycetes
Chloroflexi
Verrucomicrobia
Bacteroidetes
Gemmatimonadetes
Firmicutes
Armatimonadetes

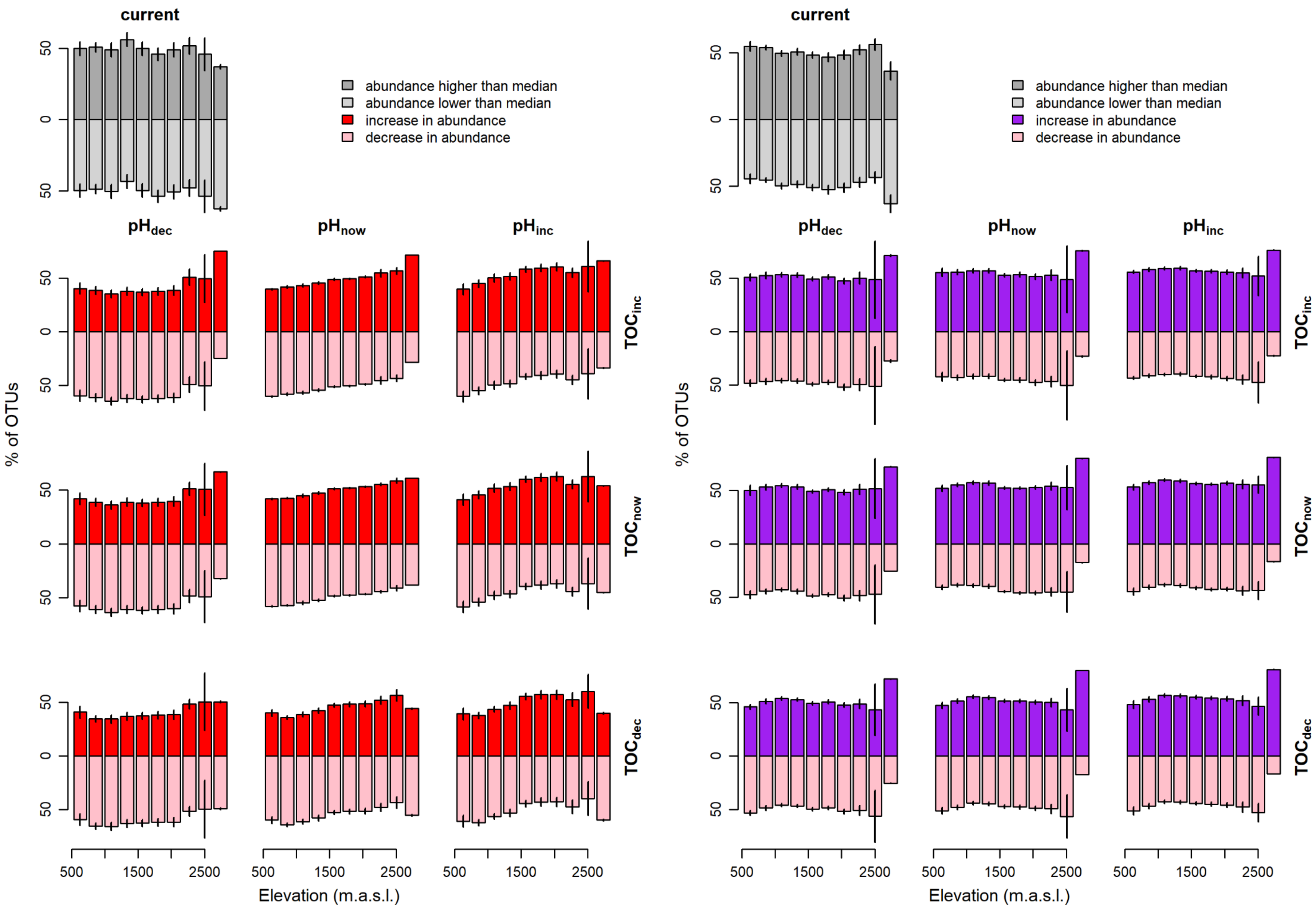


*Figure S16: Based on **DN: cl20**, GAMnb and GBM, and projections to 229 individual sites shown against elevation, proportion of OTUs having higher and lower than median abundance as predicted under current conditions (top-left corner), and proportion of OTUs predicted to have increase and decrease in abundance in the sites between current and future projections (3×3 panels).*

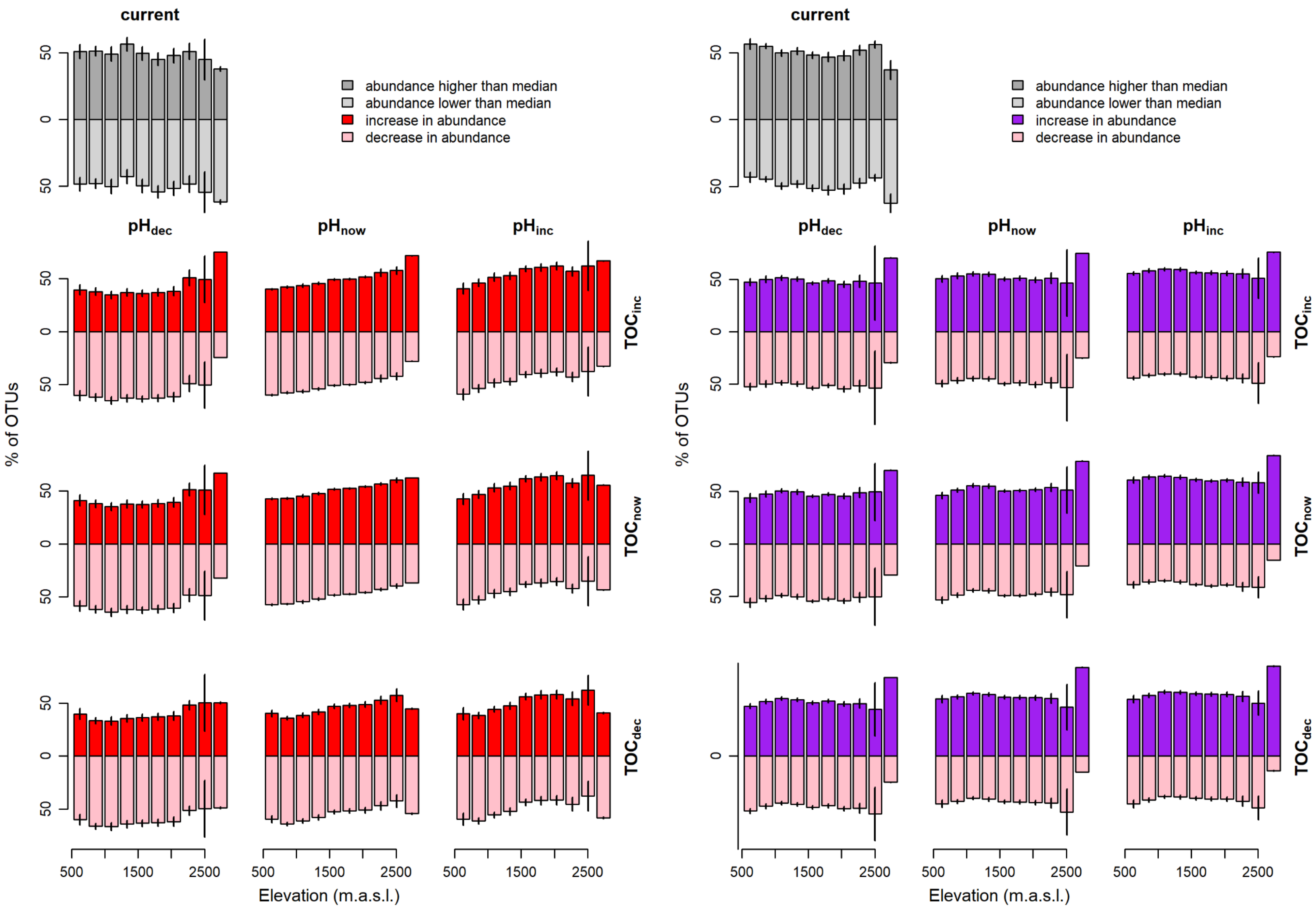


*Figure S17: Based on **DN: cl40**, GAMnb and GBM, and projections to 229 individual sites shown against elevation, proportion of OTUs having higher and lower than median abundance as predicted under current conditions (top-left corner), and proportion of OTUs predicted to have increase and decrease in abundance in the sites between current and future projections (3×3 panels).*

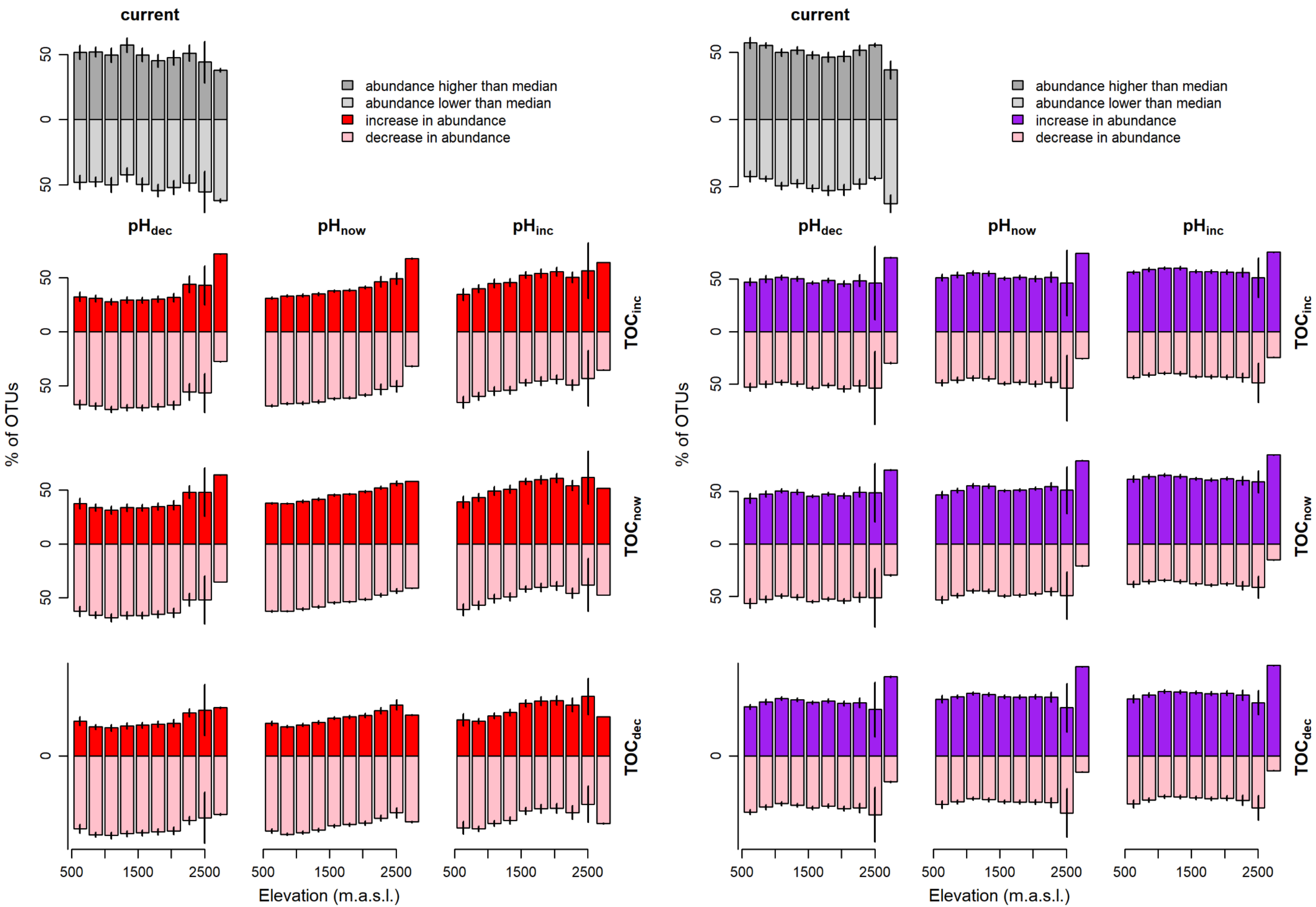


*Figure S18: Based on **DN: cl60**, GAMnb and GBM, and projections to 229 individual sites shown against elevation, proportion of OTUs having higher and lower than median abundance as predicted under current conditions (top-left corner), and proportion of OTUs predicted to have increase and decrease in abundance in the sites between current and future projections (3×3 panels).*

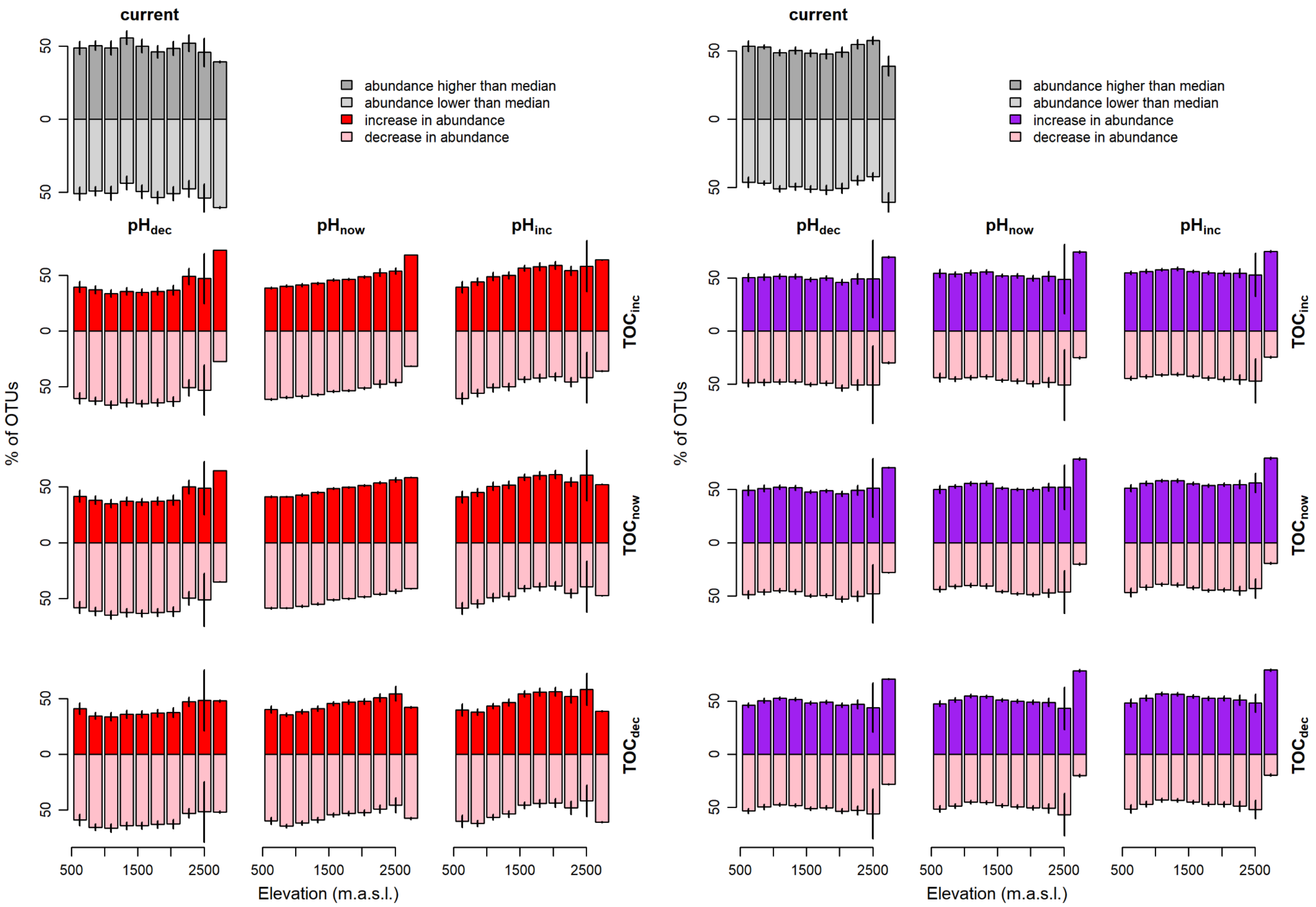


*Figure S19: Based on **DNn: cl20**, **GAMnb** and **GBM**, and projections to 229 individual sites shown against elevation, proportion of OTUs having higher and lower than median abundance as predicted under current conditions (top-left corner), and proportion of OTUs predicted to have increase and decrease in abundance in the sites between current and future projections (3×3 panels).*

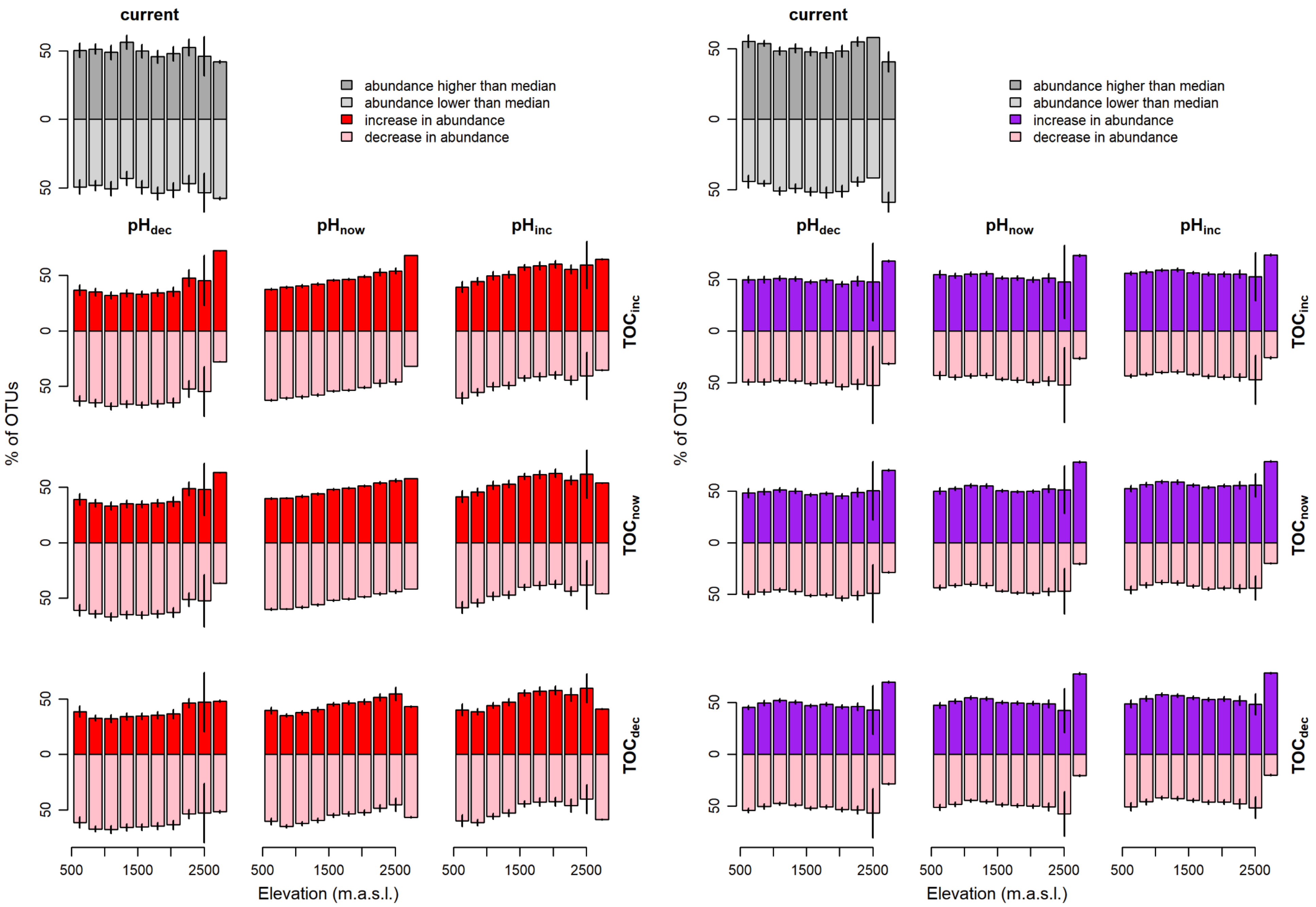


*Figure S20: Based on **DNn: cl40**, GAMnb and GBM, and projections to 229 individual sites shown against elevation, proportion of OTUs having higher and lower than median abundance as predicted under current conditions (top-left corner), and proportion of OTUs predicted to have increase and decrease in abundance in the sites between current and future projections (3×3 panels).*

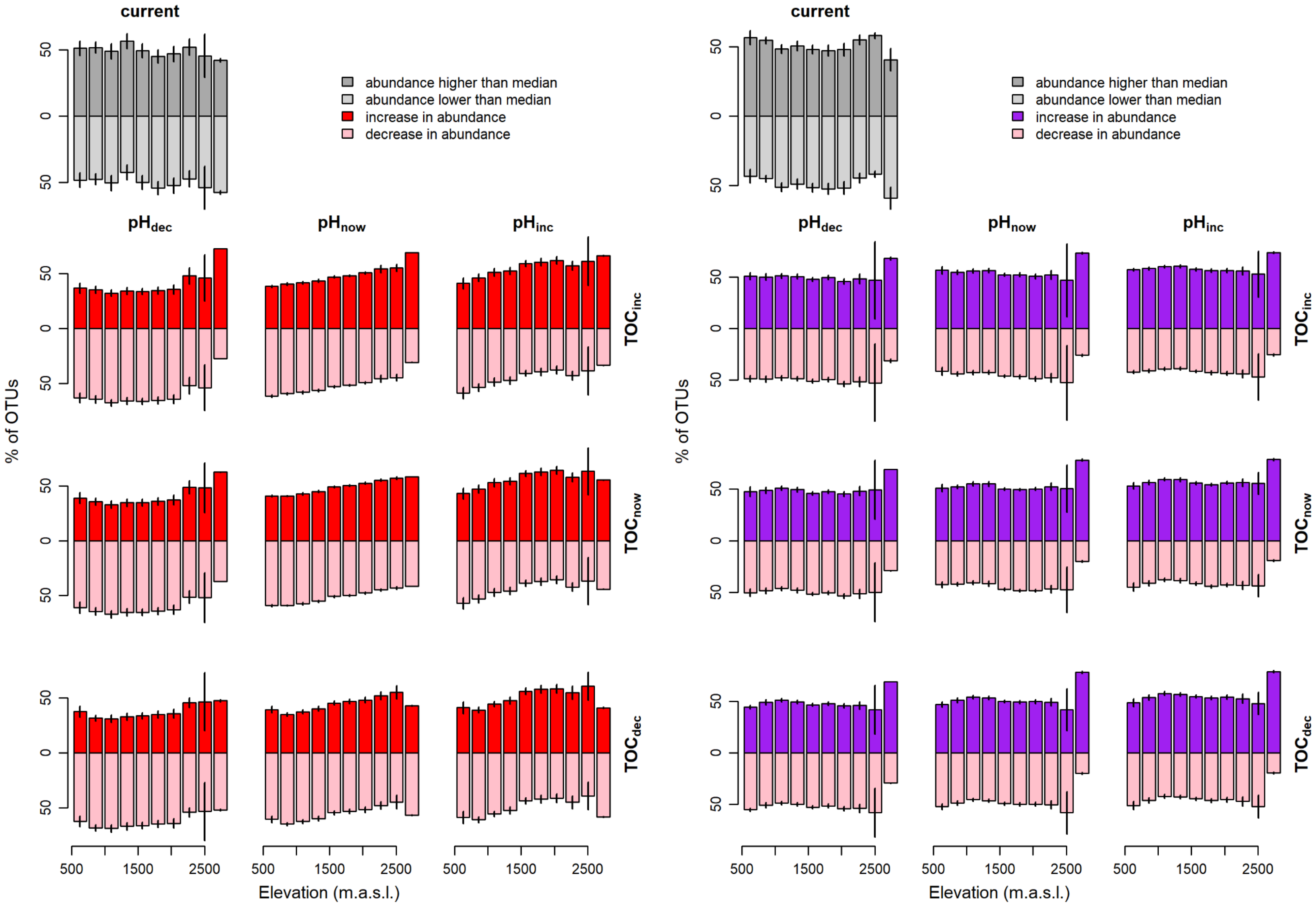


*Figure S21: Based on **DNn: cl40**, GAMnb and GBM, and projections to 229 individual sites shown against elevation, proportion of OTUs having higher and lower than median abundance as predicted under current conditions (top-left corner), and proportion of OTUs predicted to have increase and decrease in abundance in the sites between current and future projections (3×3 panels).*

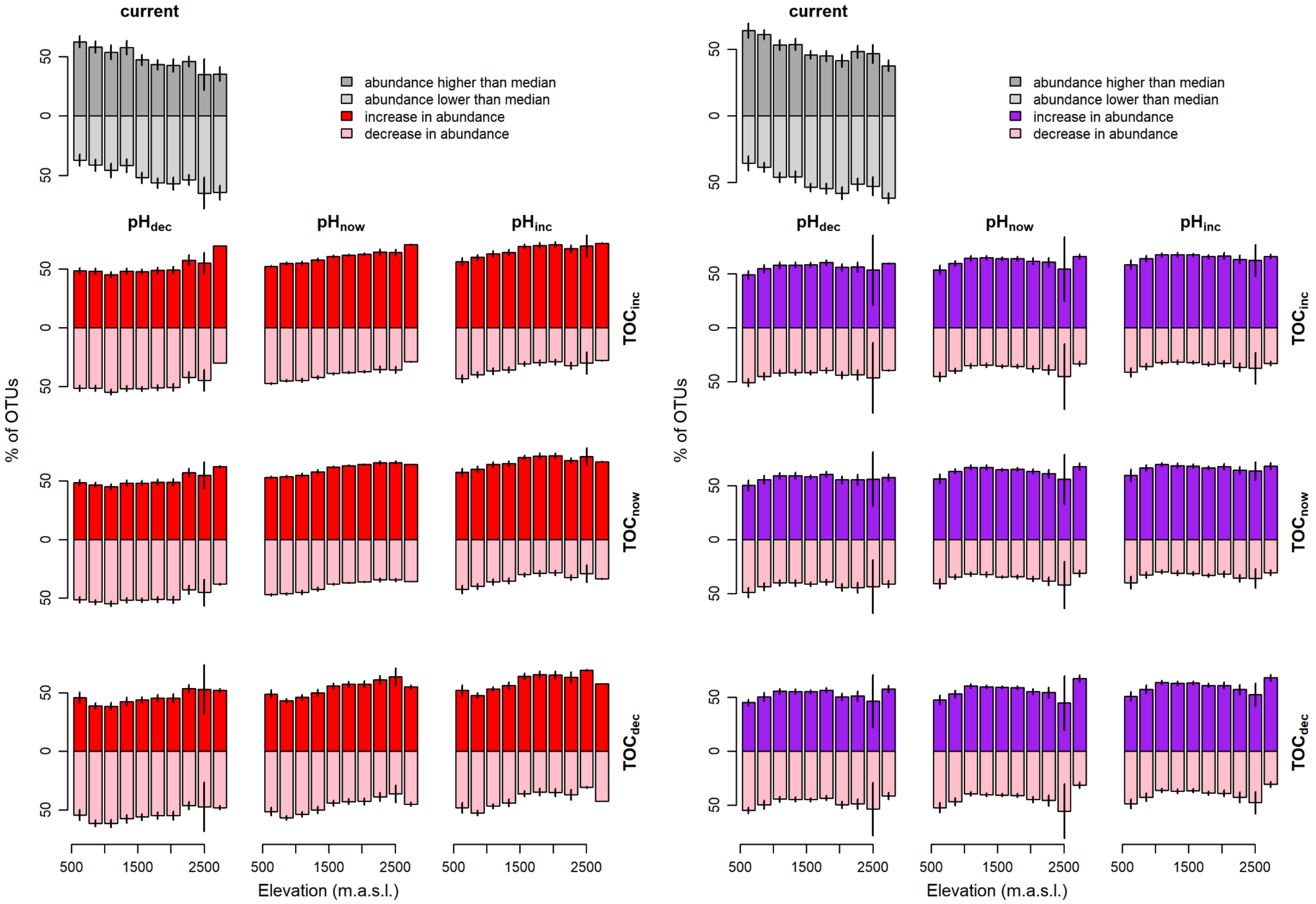


*Figure S22: Based on **CR: genus**, **GAMnb** and **GBM**, and projections to 229 individual sites shown against elevation, proportion of OTUs having higher and lower than median abundance as predicted under current conditions (top-left corner), and proportion of OTUs predicted to have increase and decrease in abundance in the sites between current and future projections (3×3 panels).*

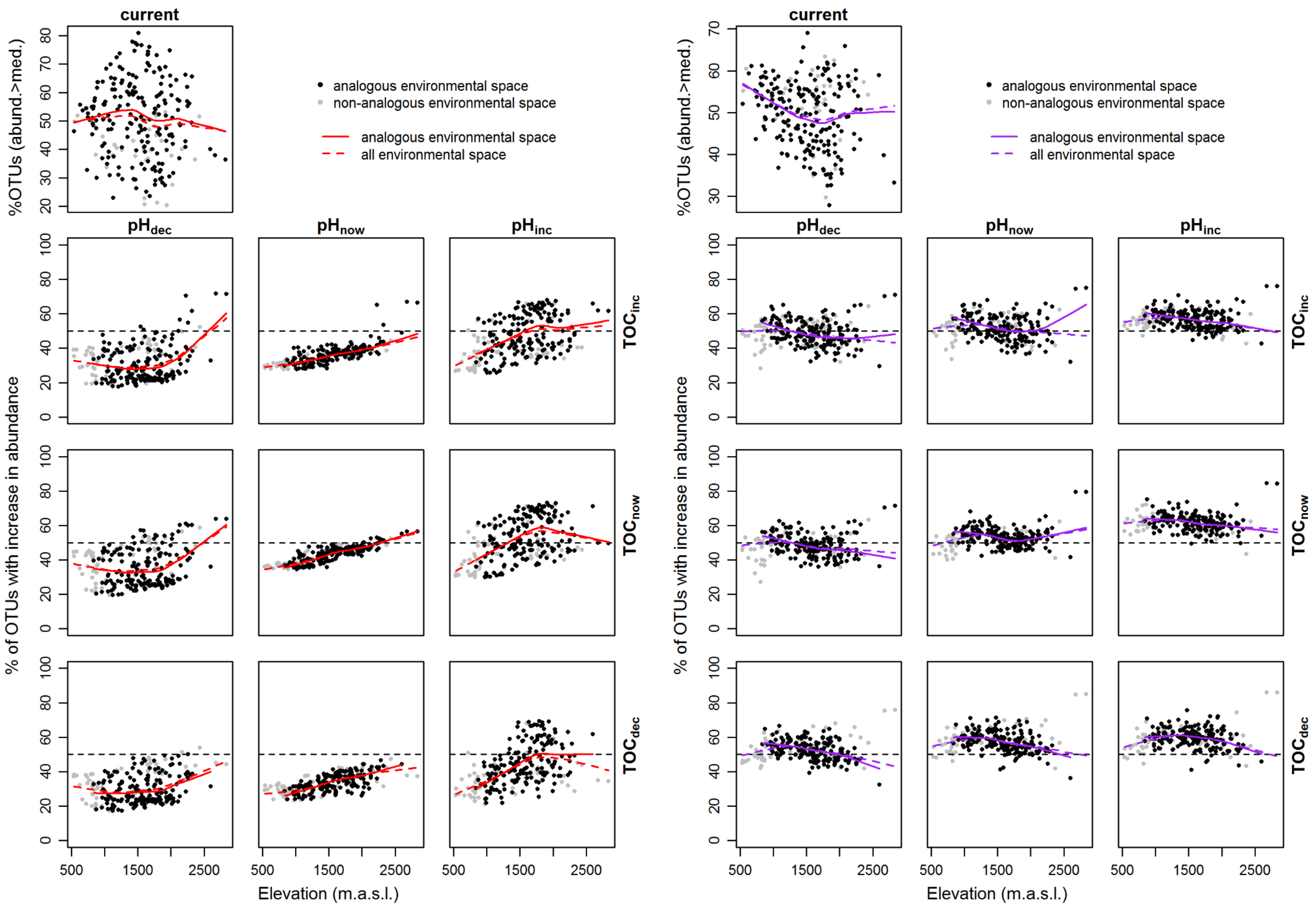


*Figure S23: Based on* ***DN: cl20****,* ***GAMnb*** *and* ***GBM****, and projections to 229 individual sites shown against elevation, proportion of OTUs having higher than median abundance as predicted under current conditions (top-left corner), and proportion of OTUs predicted to have increase in abundance in the sites between current and future projections (3×3 panels).*

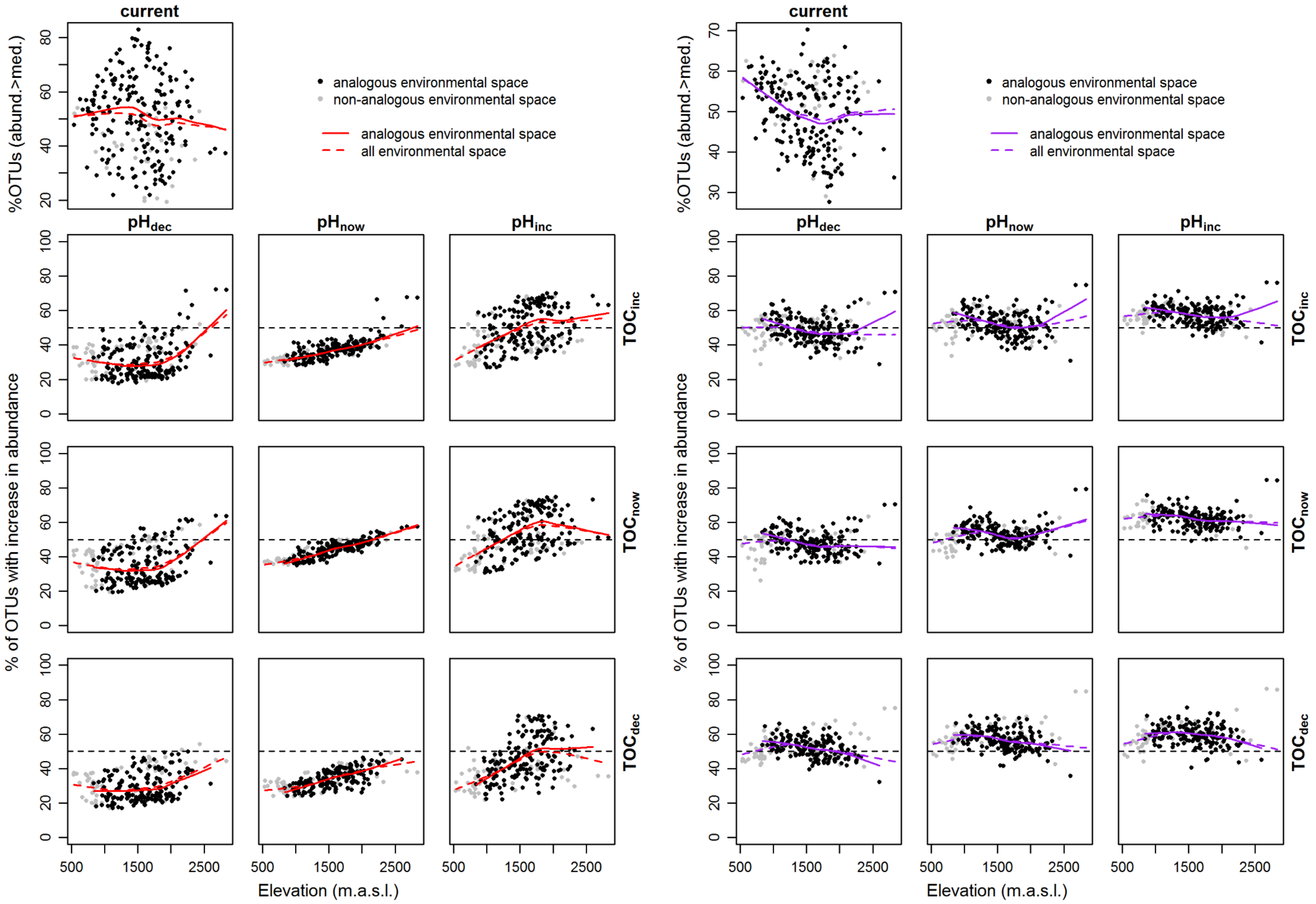


*Figure S24: Based on **DN: cl40**, **GAMnb** and **GBM**, and projections to 229 individual sites shown against elevation, proportion of OTUs having higher than median abundance as predicted under current conditions (top-left corner), and proportion of OTUs predicted to have increase in abundance in the sites between current and future projections (3×3 panels).*

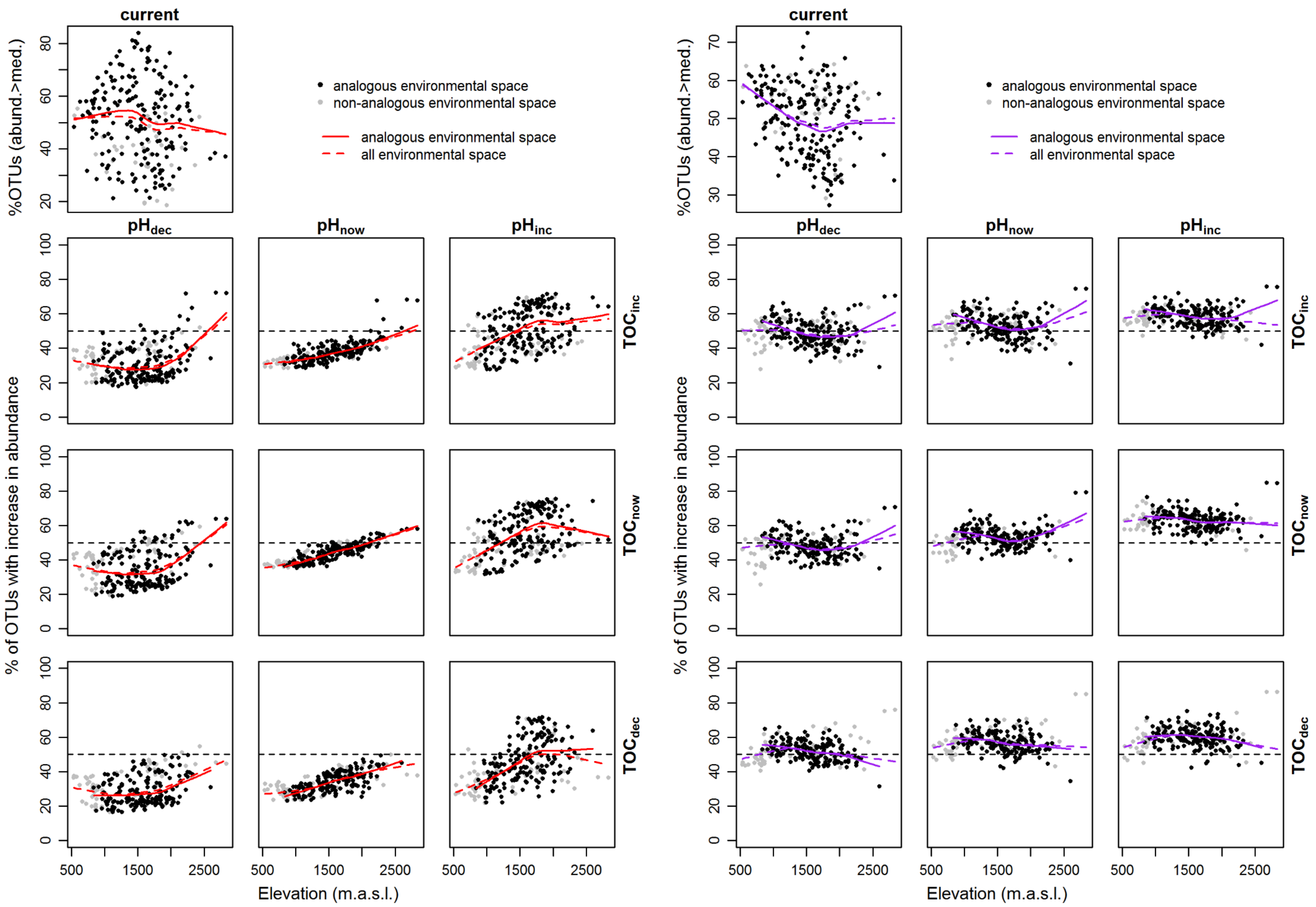


*Figure S25: Based on* ***DN: cl60***, ***GAMnb*** *and* ***GBM****, and projections to 229 individual sites shown against elevation, proportion of OTUs having higher than median abundance as predicted under current conditions (top-left corner), and proportion of OTUs predicted to have increase in abundance in the sites between current and future projections (3×3 panels).*

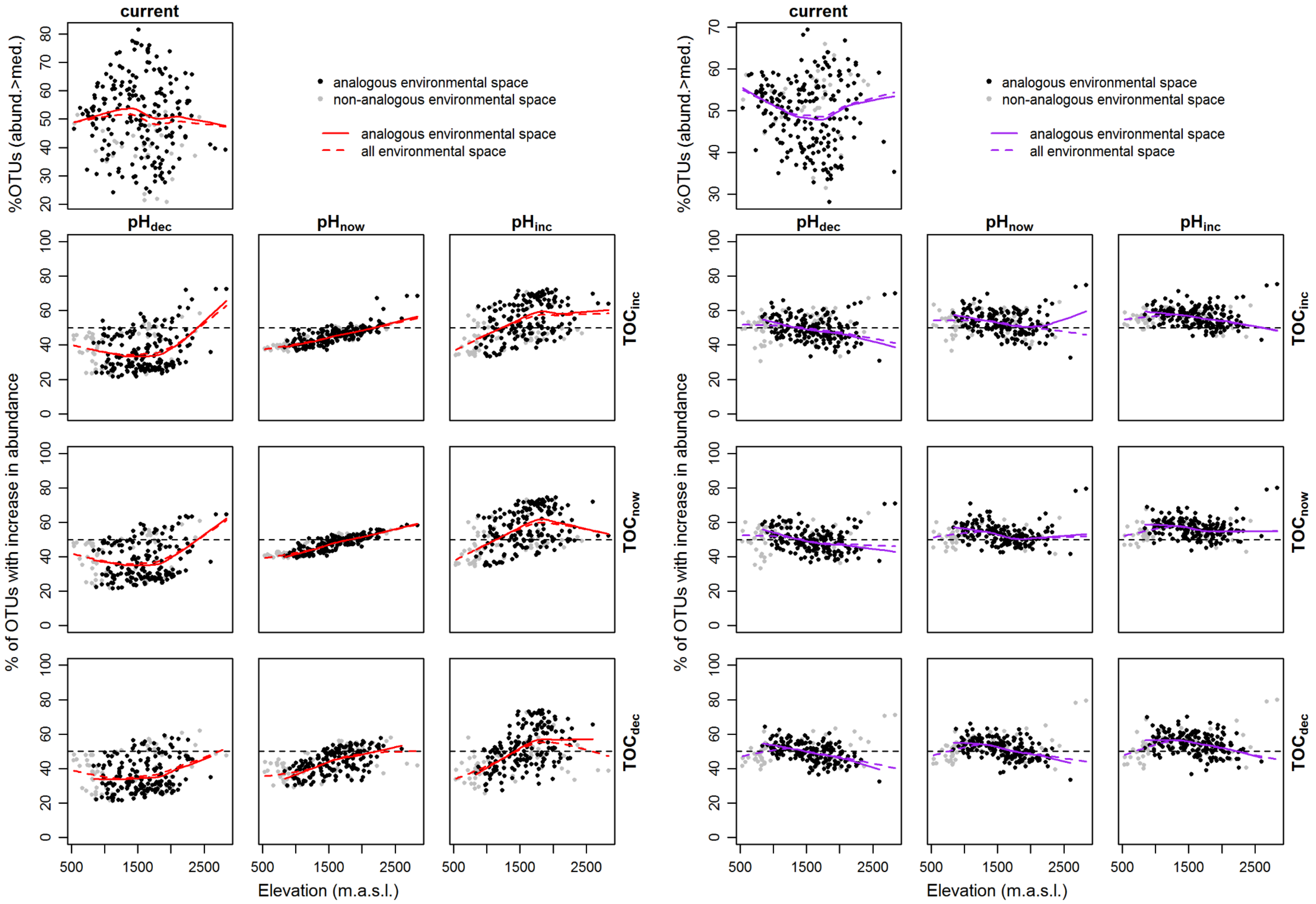


*Figure S26: Based on **DNn: cl20**, **GAMnb** and **GBM**, and projections to 229 individual sites shown against elevation, proportion of OTUs having higher than median abundance as predicted under current conditions (top-left corner), and proportion of OTUs predicted to have increase in abundance in the sites between current and future projections (3×3 panels).*

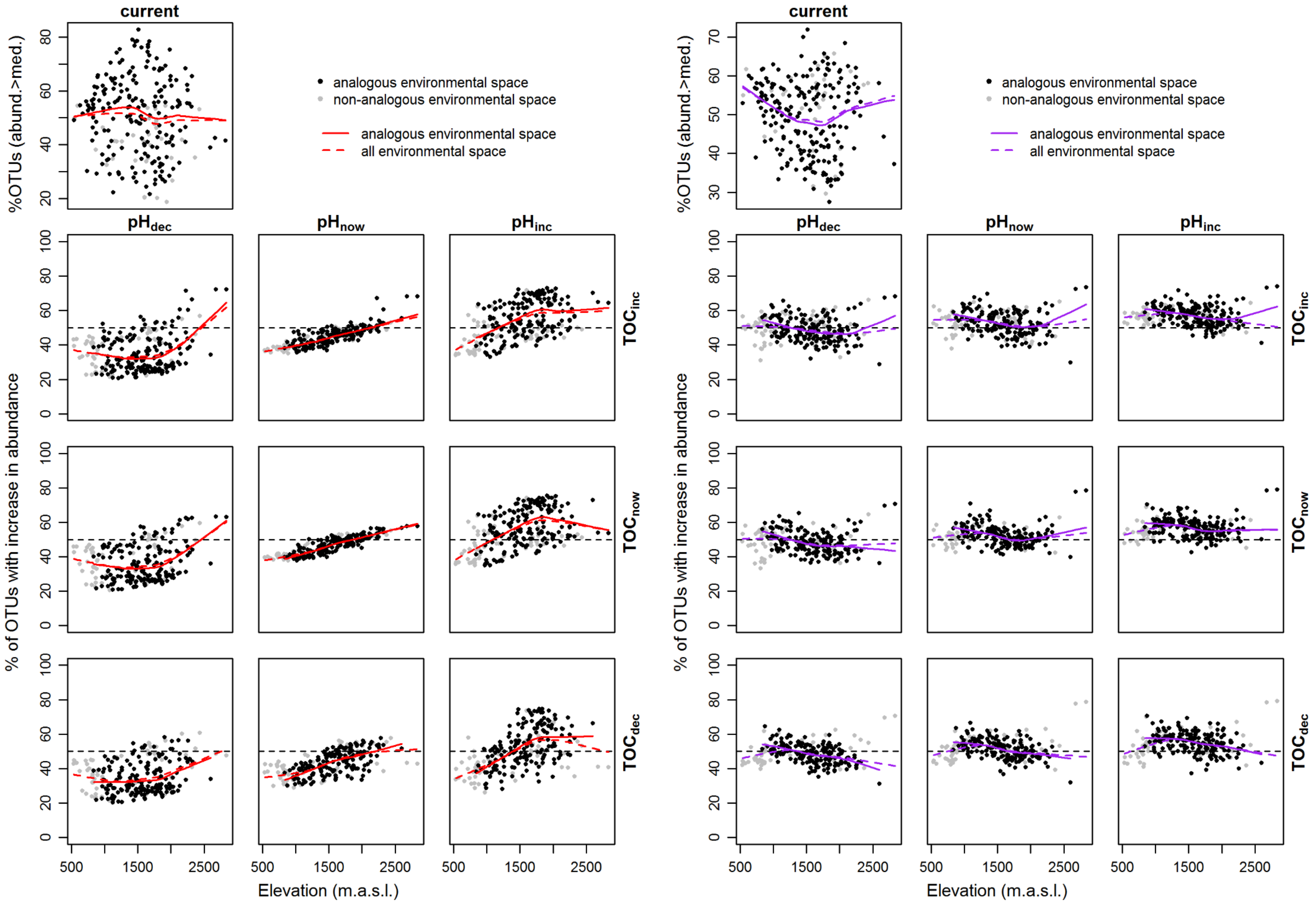


*Figure S27: Based on **DNn: cl40**, **GAMnb** and **GBM**, and projections to 229 individual sites shown against elevation, proportion of OTUs having higher than median abundance as predicted under current conditions (top-left corner), and proportion of OTUs predicted to have increase in abundance in the sites between current and future projections (3×3 panels).*

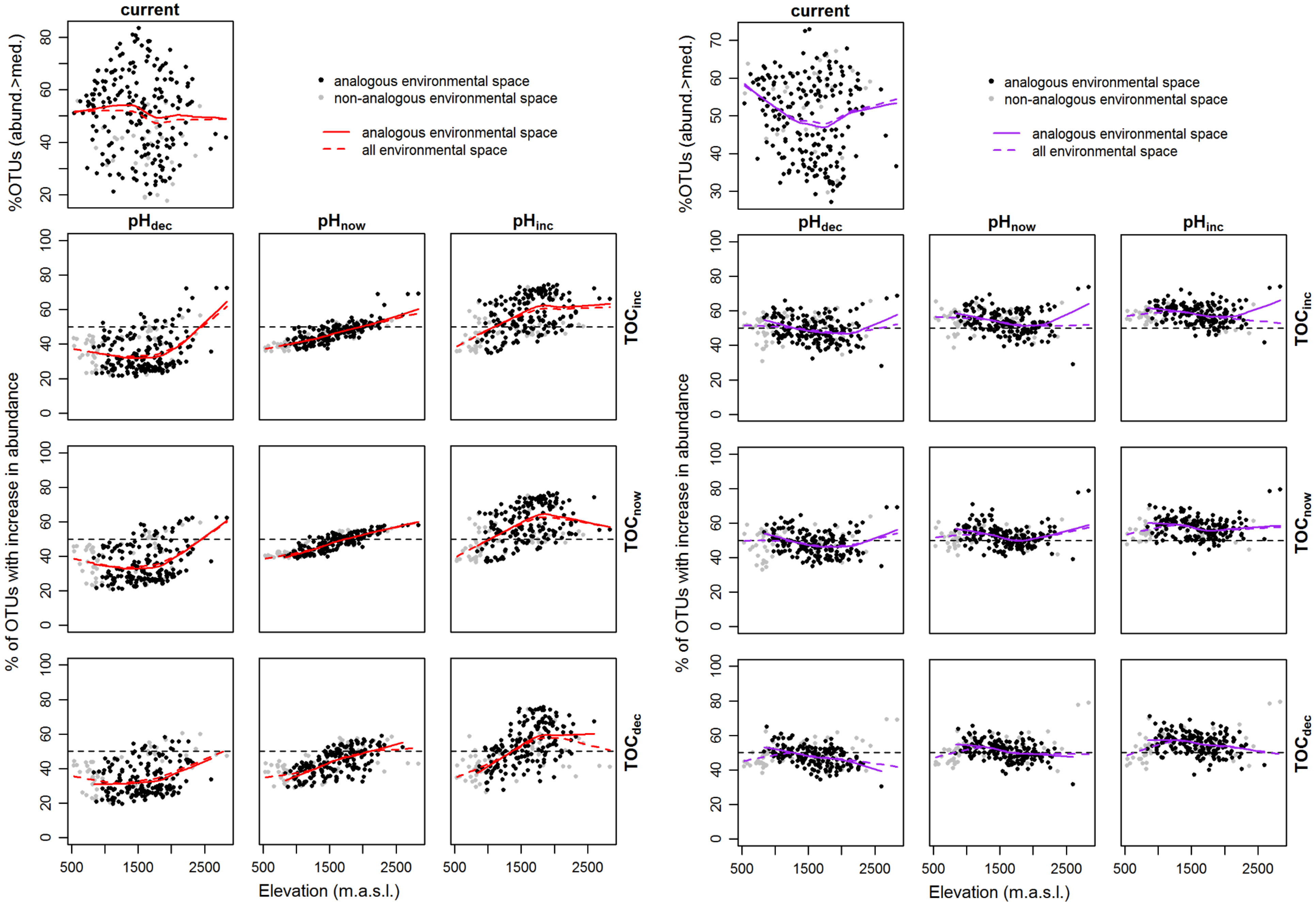


*Figure S28: Based on **DNn: cl60**, GAMnb and GBM, and projections to 229 individual sites shown against elevation, % of OTUs having higher than median abundance as predicted under current conditions (top-left corner), and % of OTUs predicted to have increase in abundance in the sites between current and future projections (3×3 panels).*

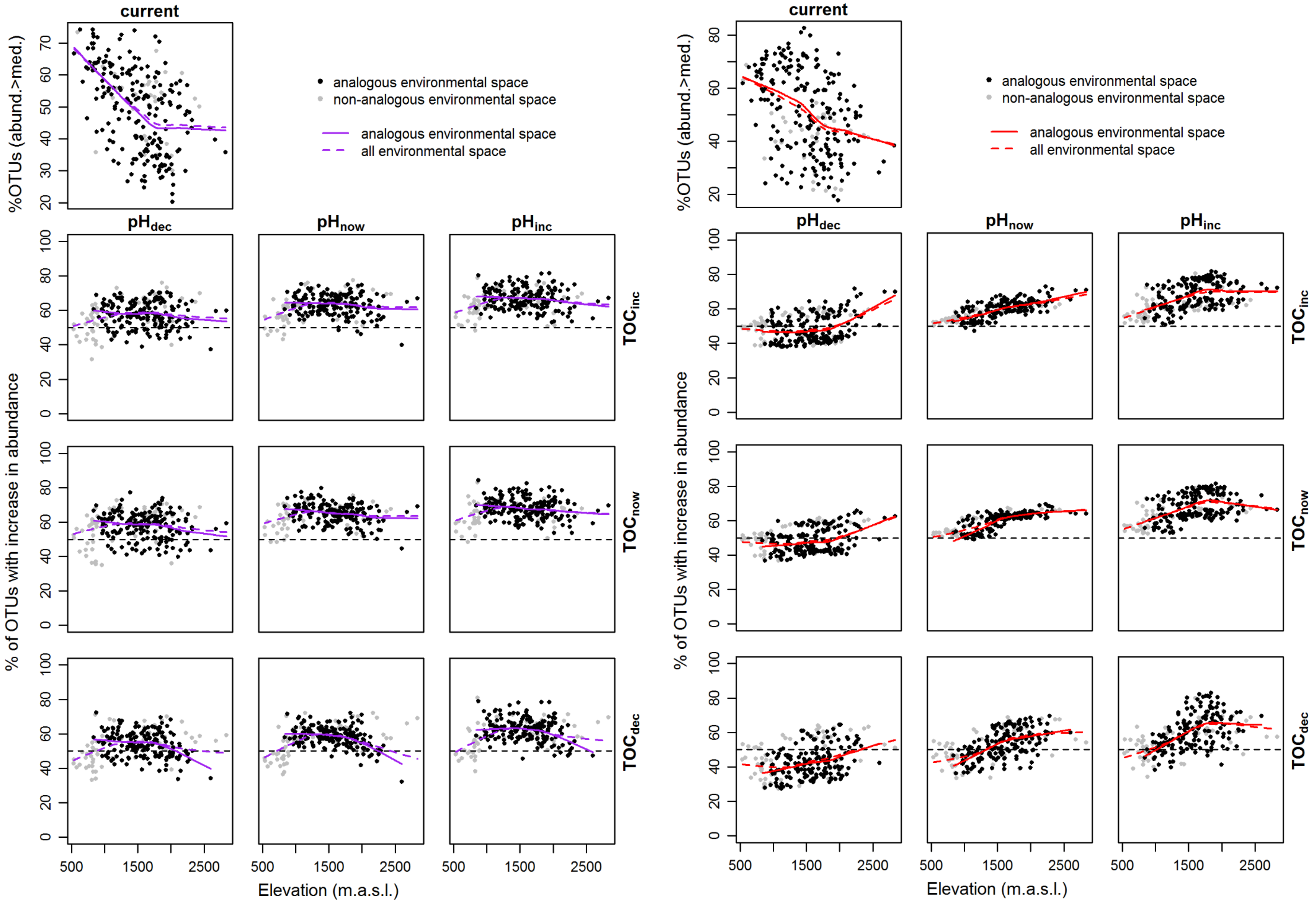


*Figure S29: Based on **CR: genus**, **GAMnb** and **GBM**, and projections to 229 individual sites shown against elevation, proportion of OTUs having higher than median abundance as predicted under current conditions (top-left corner), and proportion of OTUs predicted to have increase in abundance in the sites between current and future projections (3×3 panels).*

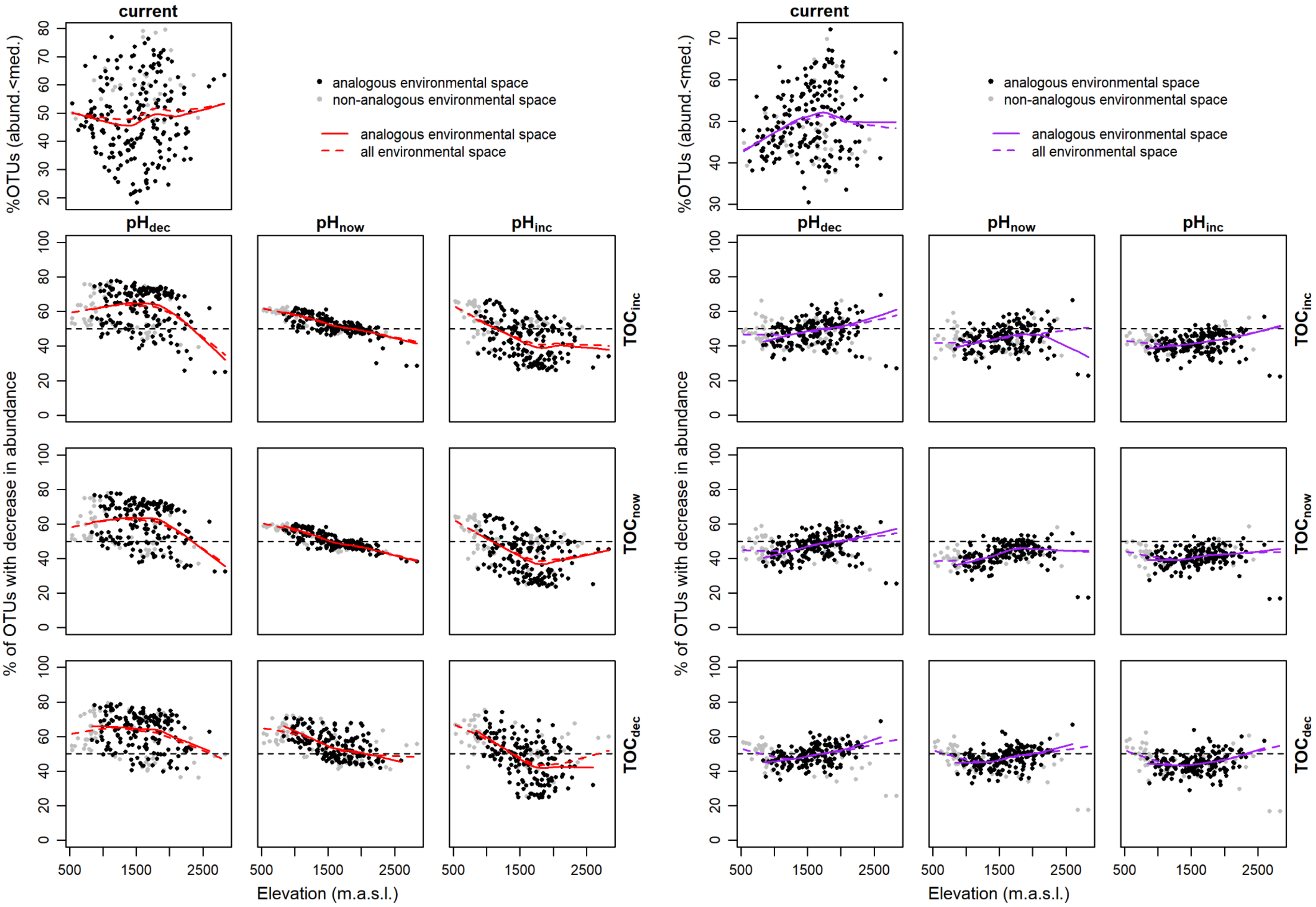


*Figure S30: Based on **DN: cl20**, **GAMnb** and **GBM**, and projections to 229 individual sites shown against elevation, proportion of OTUs having lower than median abundance as predicted under current conditions (top-left corner), and proportion of OTUs predicted to have decrease in abundance in the sites between current and future projections (3×3 panels).*

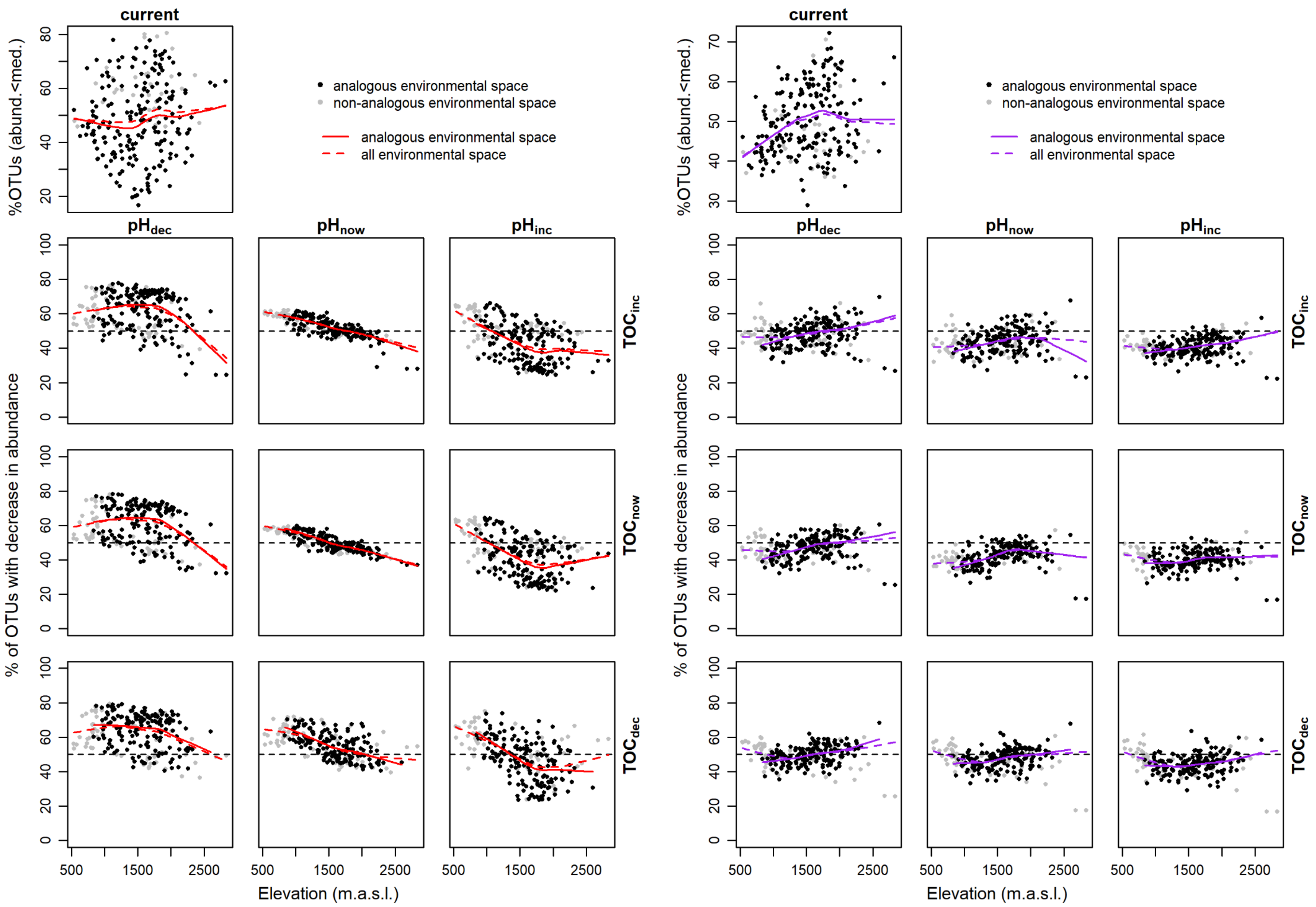


*Figure S31: Based on **DN: cl40**, **GAMnb** and **GBM**, and projections to 229 individual sites shown against elevation, proportion of OTUs having lower than median abundance as predicted under current conditions (top-left corner), and proportion of OTUs predicted to have decrease in abundance in the sites between current and future projections (3×3 panels).*

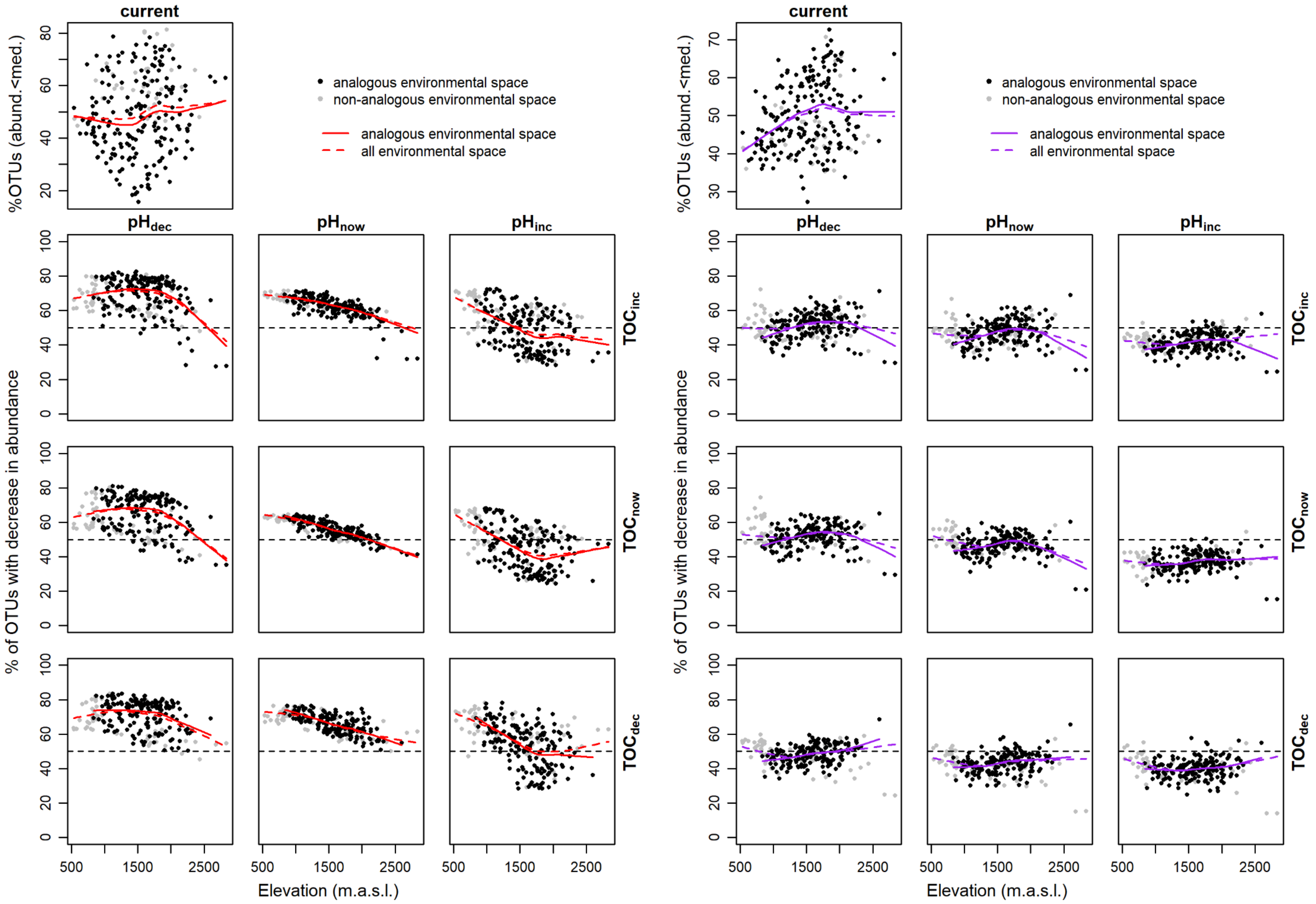


*Figure S32: Based on **DN: cl60**, **GAMnb** and **GBM**, and projections to 229 individual sites shown against elevation, proportion of OTUs having lower than median abundance as predicted under current conditions (top-left corner), and proportion of OTUs predicted to have decrease in abundance in the sites between current and future projections (3×3 panels).*

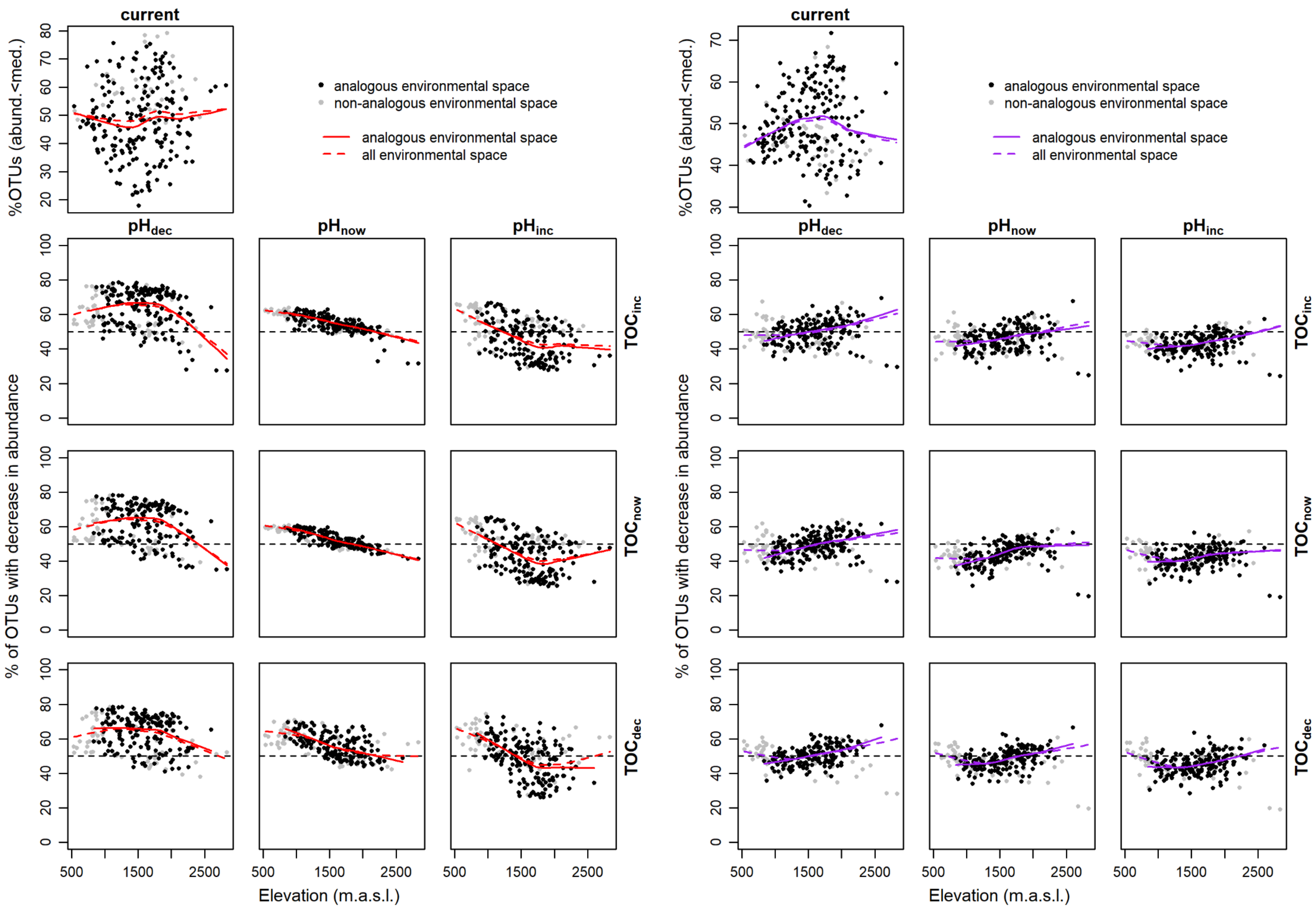


*Figure S33: Based on **DNn: cl20**, **GAMnb** and **GBM**, and projections to 229 individual sites shown against elevation, proportion of OTUs having lower than median abundance as predicted under current conditions (top-left corner), and proportion of OTUs predicted to have decrease in abundance in the sites between current and future projections (3×3 panels).*

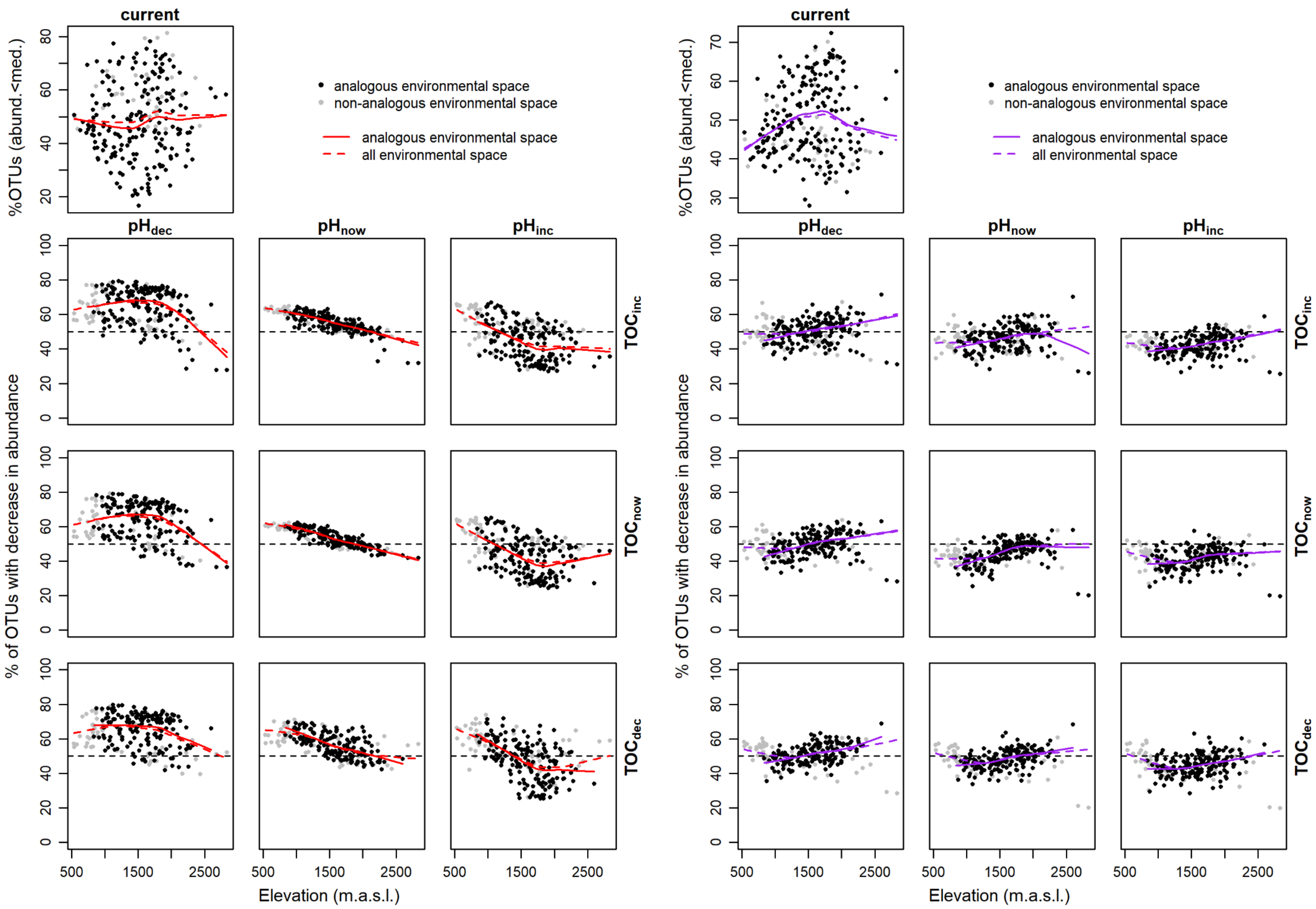


*Figure S34: Based on **DNn: cl40**, **GAMnb** and **GBM**, and projections to 229 individual sites shown against elevation, proportion of OTUs having lower than median abundance as predicted under current conditions (top-left corner), and proportion of OTUs predicted to have decrease in abundance in the sites between current and future projections (3×3 panels).*

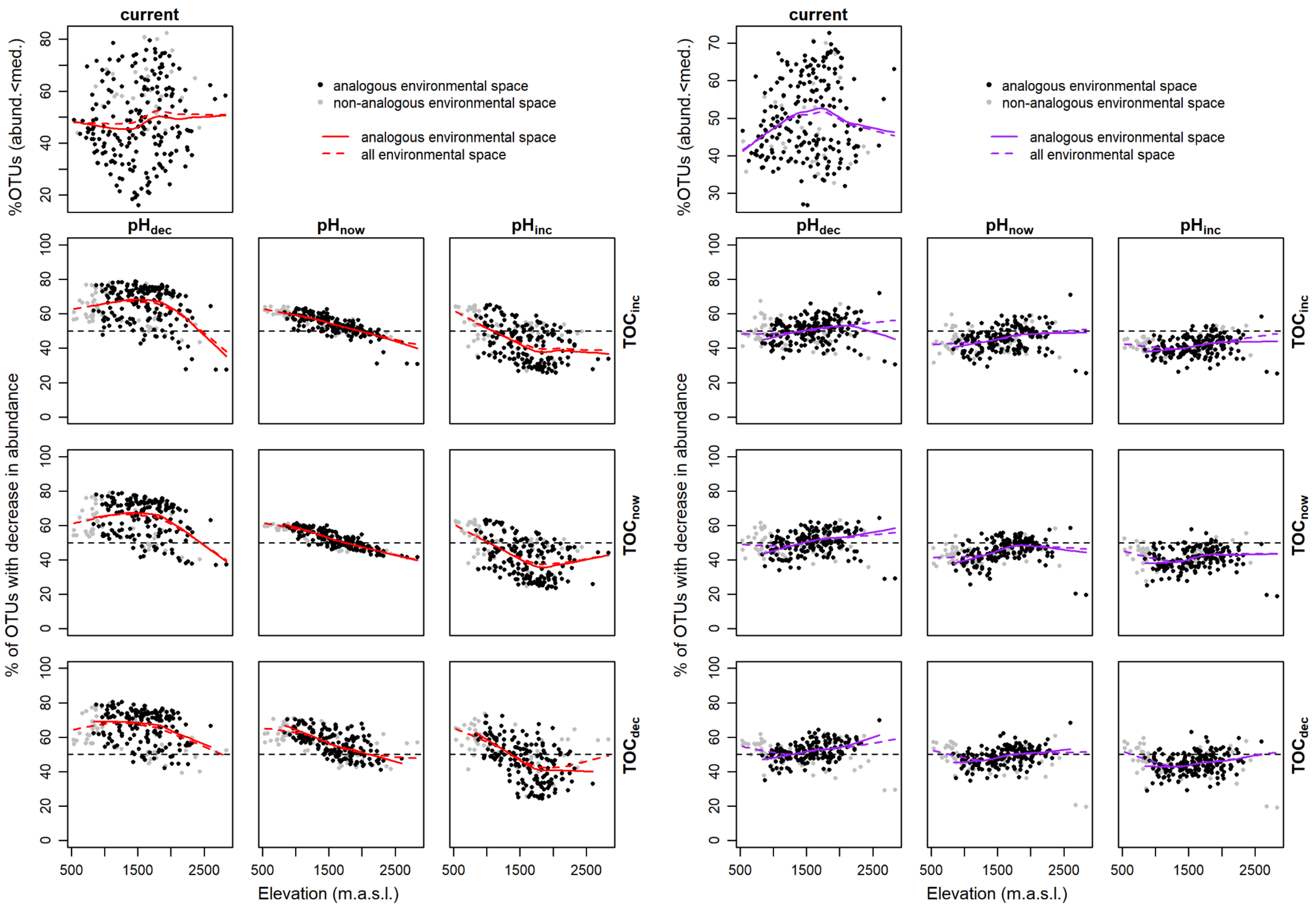


*Figure S35: Based on* ***DNn: cl60****,* ***GAMnb*** *and* ***GBM****, and projections to 229 individual sites shown against elevation, proportion of OTUs having lower than median abundance as predicted under current conditions (top-left corner), and proportion of OTUs predicted to have decrease in abundance in the sites between current and future projections (3×3 panels).*

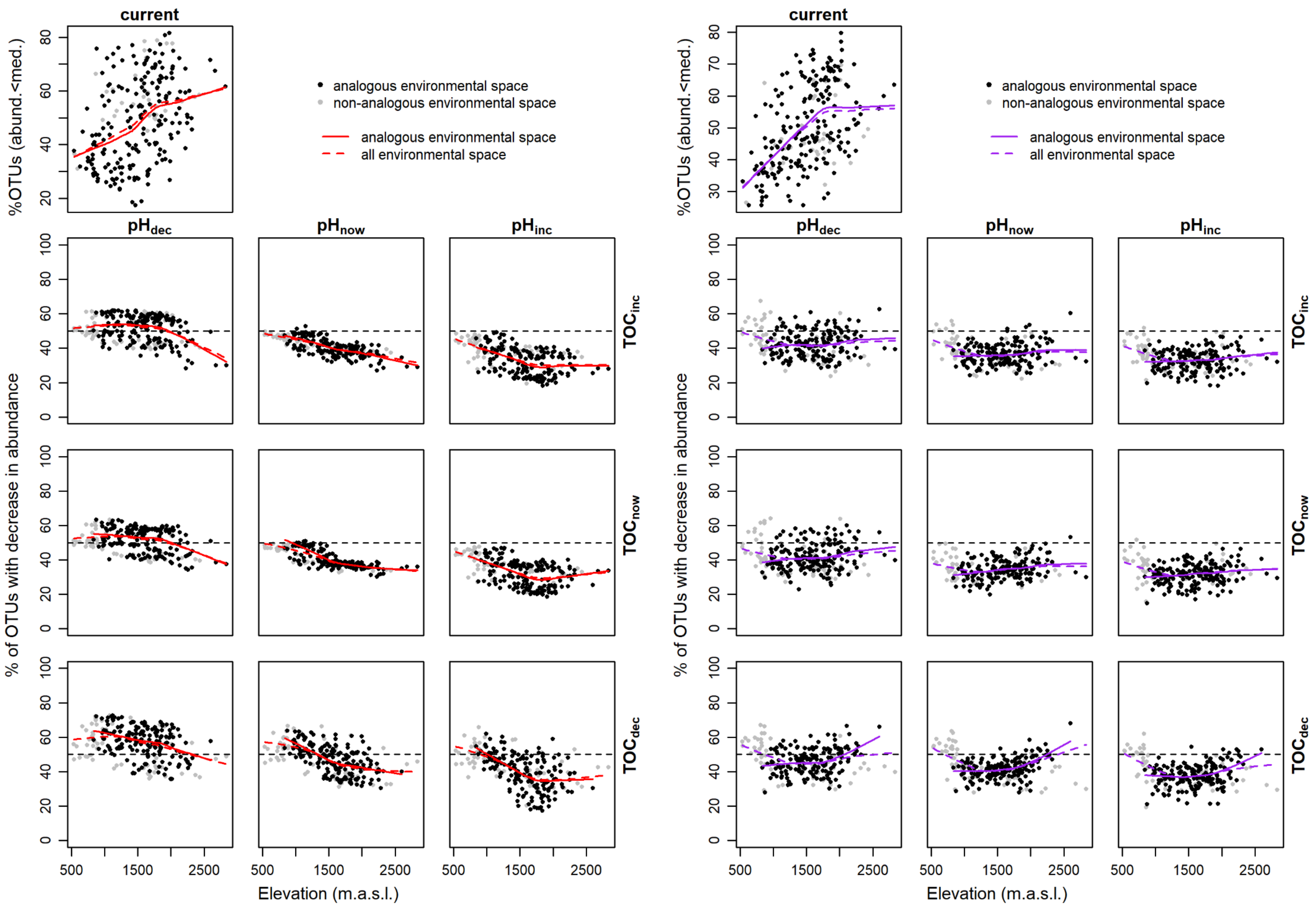


*Figure S36: Based on **CR: genus**, **GAMnb** and **GBM**, and projections to 229 individual sites shown against elevation, proportion of OTUs having lower than median abundance as predicted under current conditions (top-left corner), and proportion of OTUs predicted to have decrease in abundance in the sites between current and future projections (3×3 panels).*

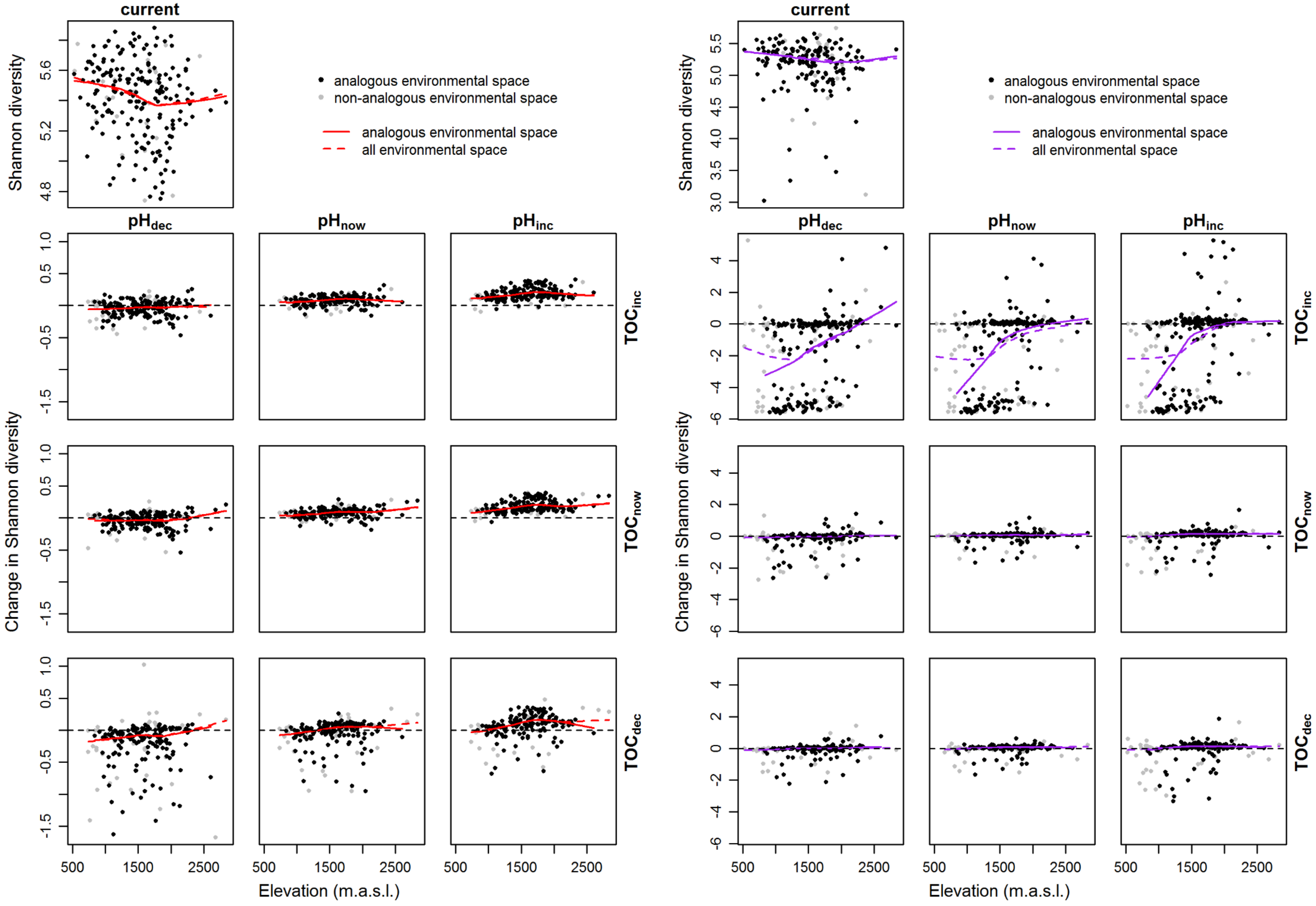


*Figure S37. Based on **DN: cl20, GAMnb** and **GBM**, and projections to 229 individual sites shown against elevation, predicted current Shannon diversity (top-left panel), and predicted changes in diversity under nine different future scenarios (3×3 panels). From the figures, sites with "outliers" (+/-1.58 × IQR/sqrt(229)) are removed.*

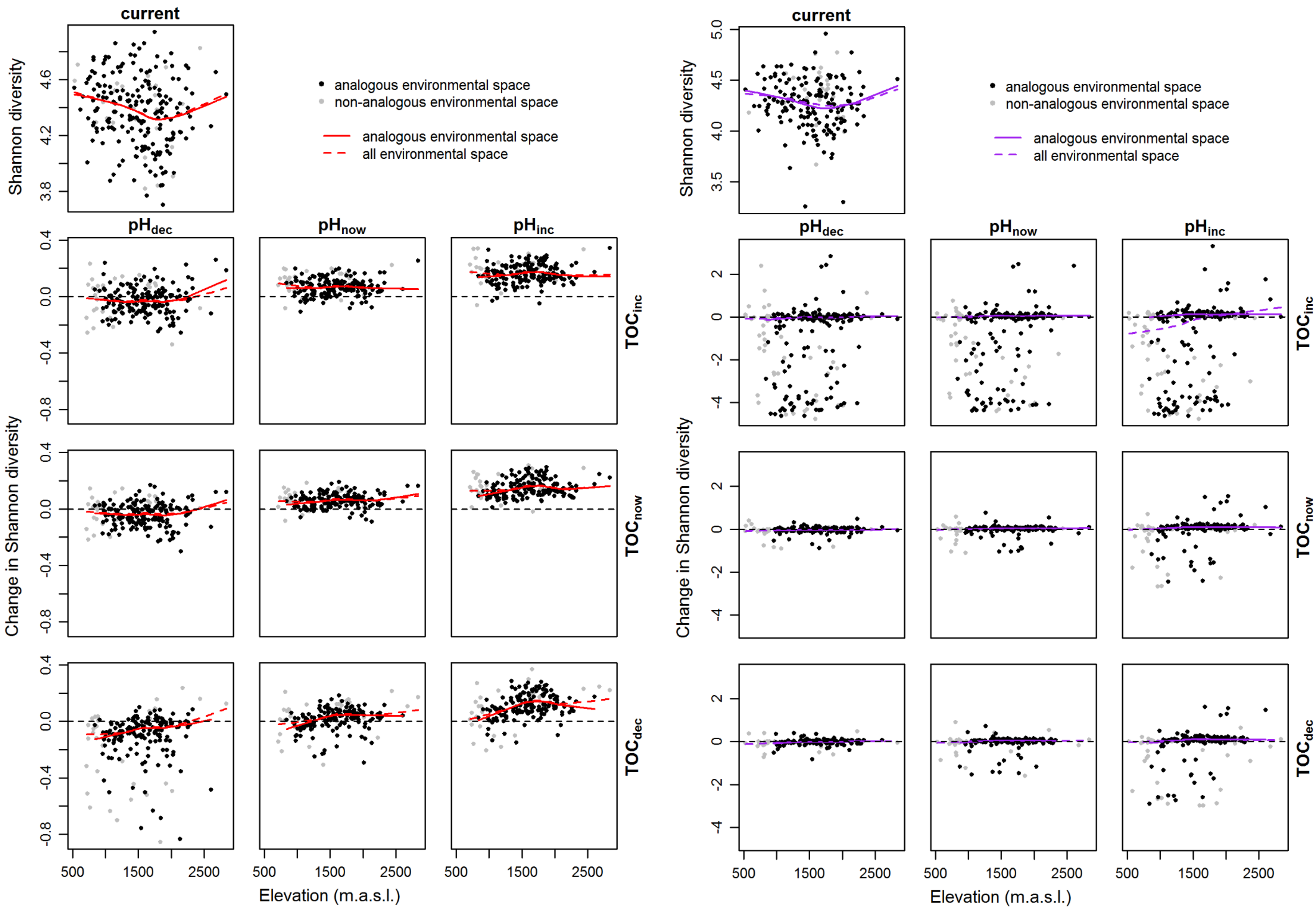


*Figure S38. Based on **DN: cl40**, **GAMnb** and **GBM**, and projections to 229 individual sites shown against elevation, predicted current Shannon diversity (top-left panel), and predicted changes in diversity under nine different future scenarios (3×3 panels). From the figures, sites with "outliers" (+/-1.58 × IQR/sqrt(229)) are removed.*

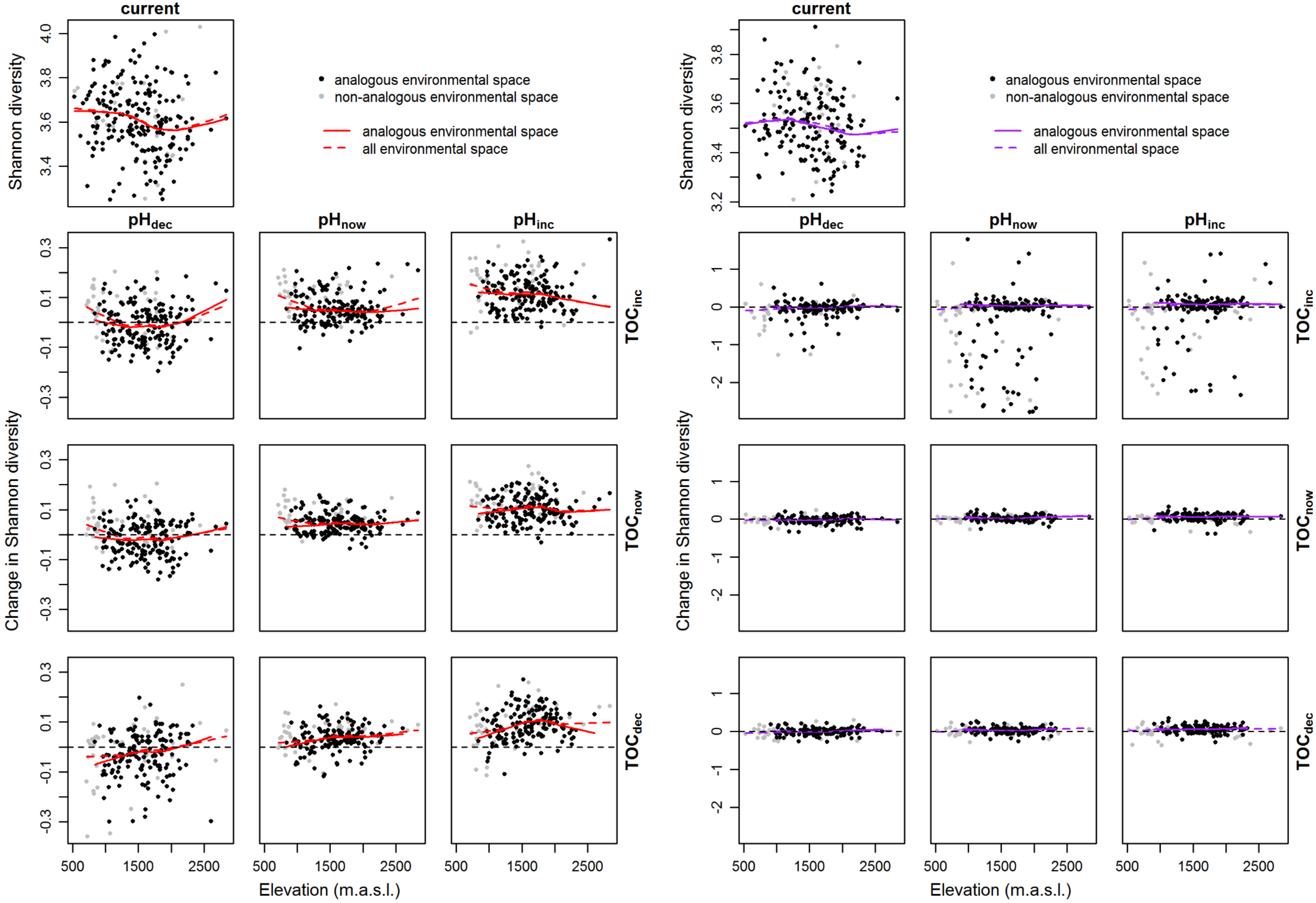


*Figure S39. Based on **DN: cl60**, **GAMnb** and **GBM**, and projections to 229 individual sites shown against elevation, predicted current Shannon diversity (top-left panel), and predicted changes in diversity under nine different future scenarios (3×3 panels). From the figures, sites with "outliers" (+/-1.58 × IQR/sqrt(229)) are removed.*

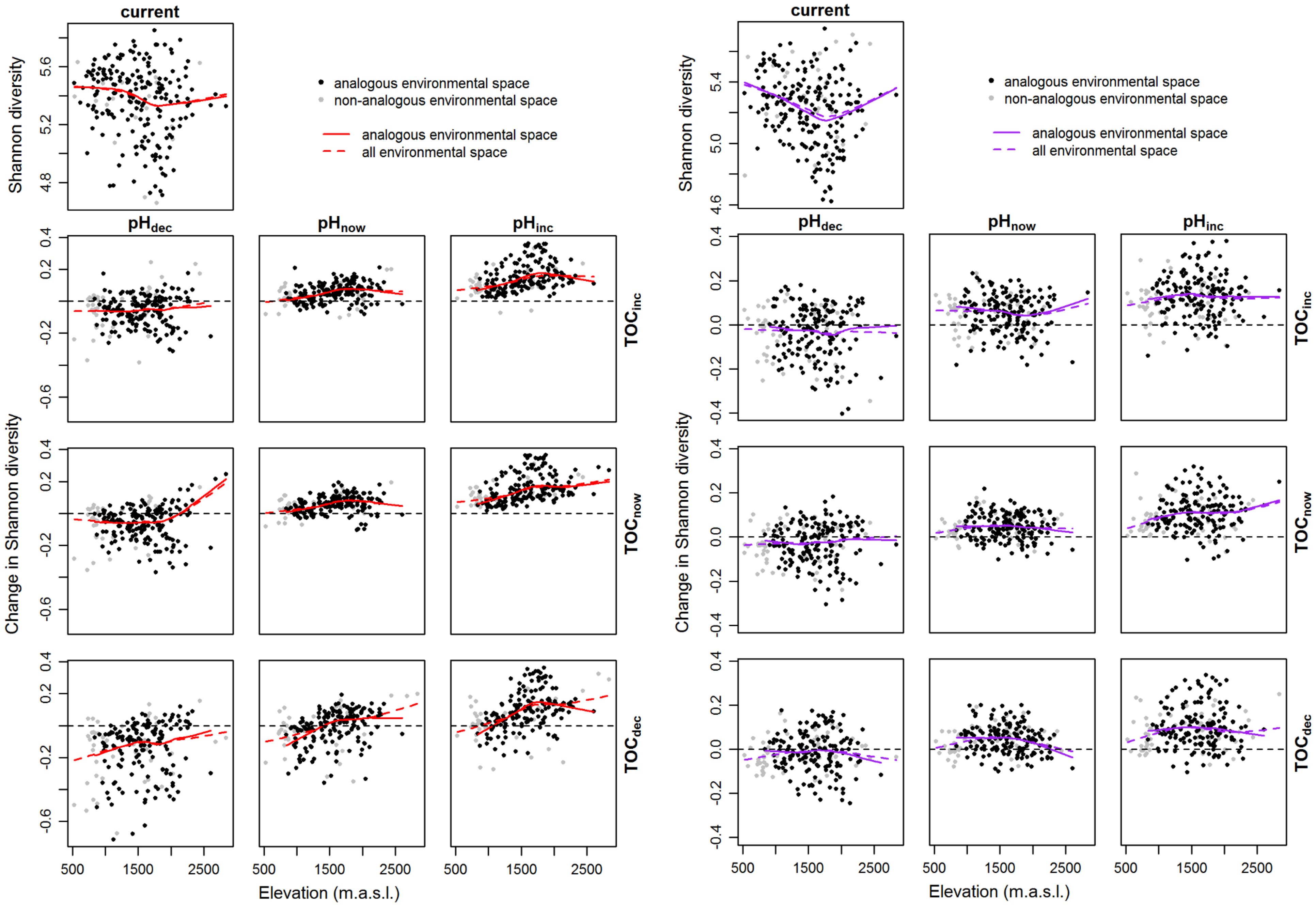


*Figure S40. Based on **DNn: cl20, GAMnb** and **GBM**, and projections to 229 individual sites shown against elevation, predicted current Shannon diversity (top-left panel), and predicted changes in diversity under nine different future scenarios (3×3 panels). From the figures, sites with "outliers" (+/-1.58 × IQR/sqrt(229)) are removed.*

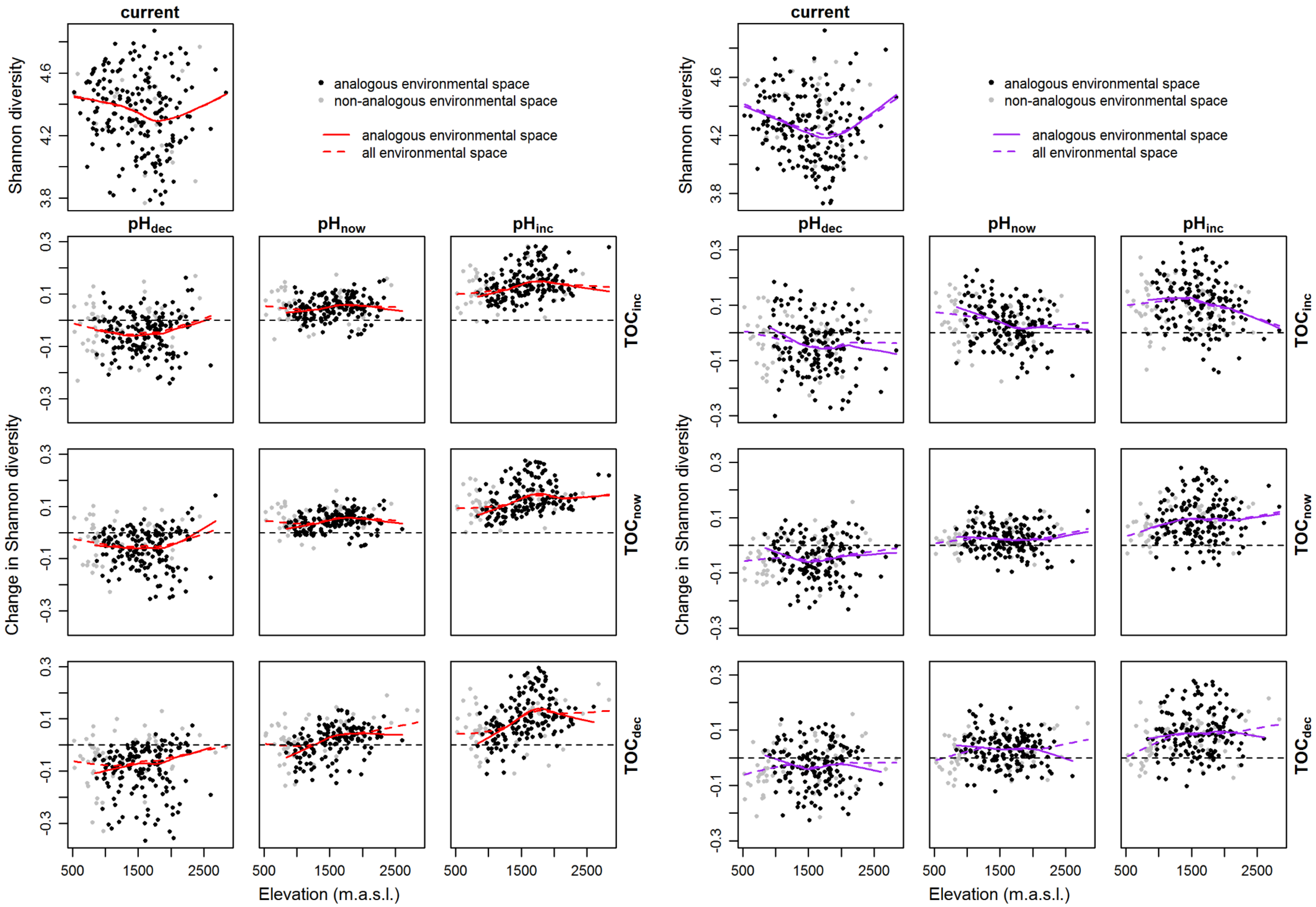


*Figure S41. Based on **DNn: cl40, GAMnb** and **GBM**, and projections to 229 individual sites shown against elevation, predicted current Shannon diversity (top-left panel), and predicted changes in diversity under nine different future scenarios (3×3 panels). From the figures, sites with "outliers" (+/-1.58 × IQR/sqrt(229)) are removed.*

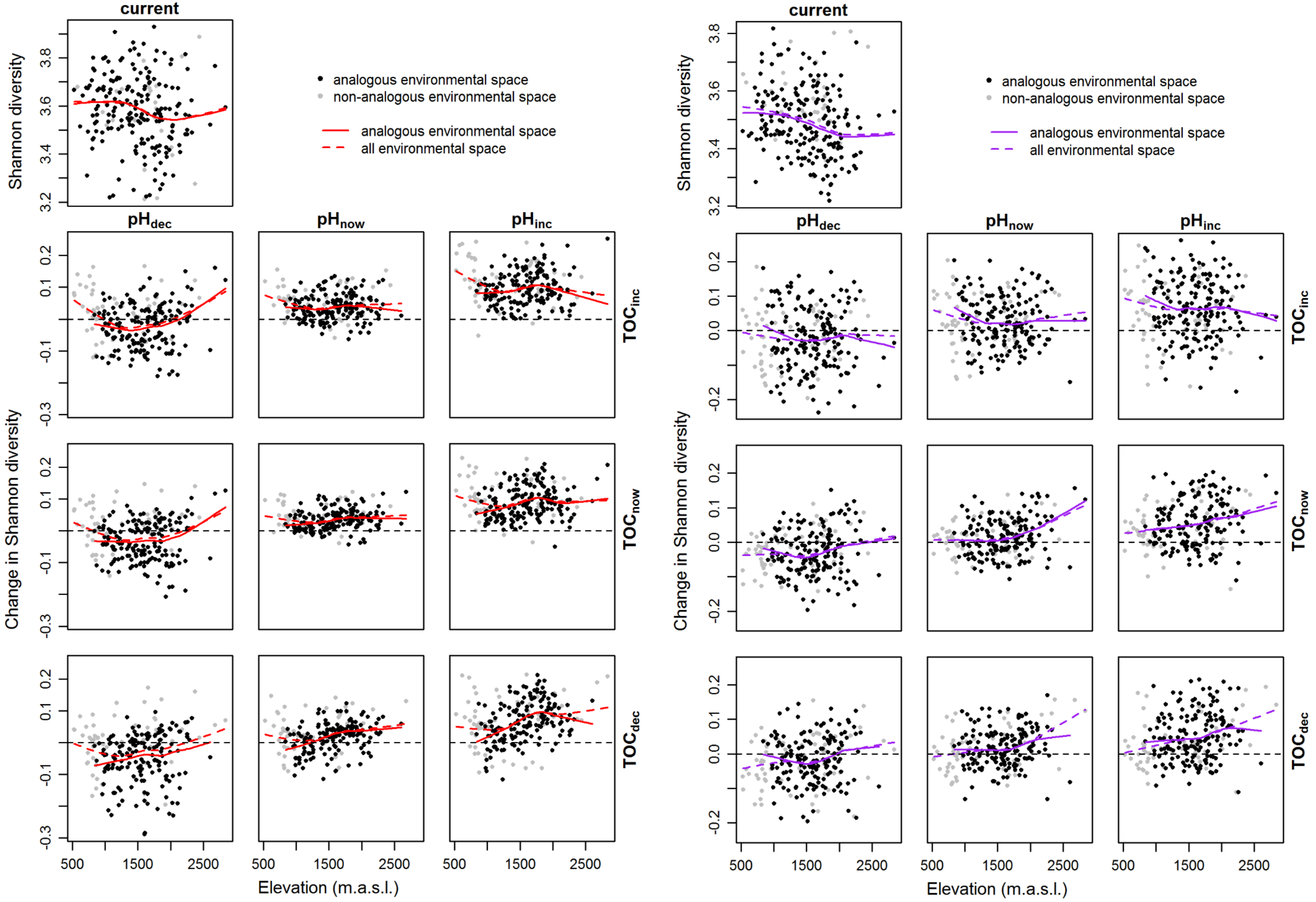


*Figure S42. Based on **DNn: cl60, GAMnb** and **GBM**, and projections to 229 individual sites shown against elevation, predicted current Shannon diversity (top-left panel), and predicted changes in diversity under nine different future scenarios (3×3 panels). From the figures, sites with "outliers" (+/-1.58 × IQR/sqrt(229)) are removed.*

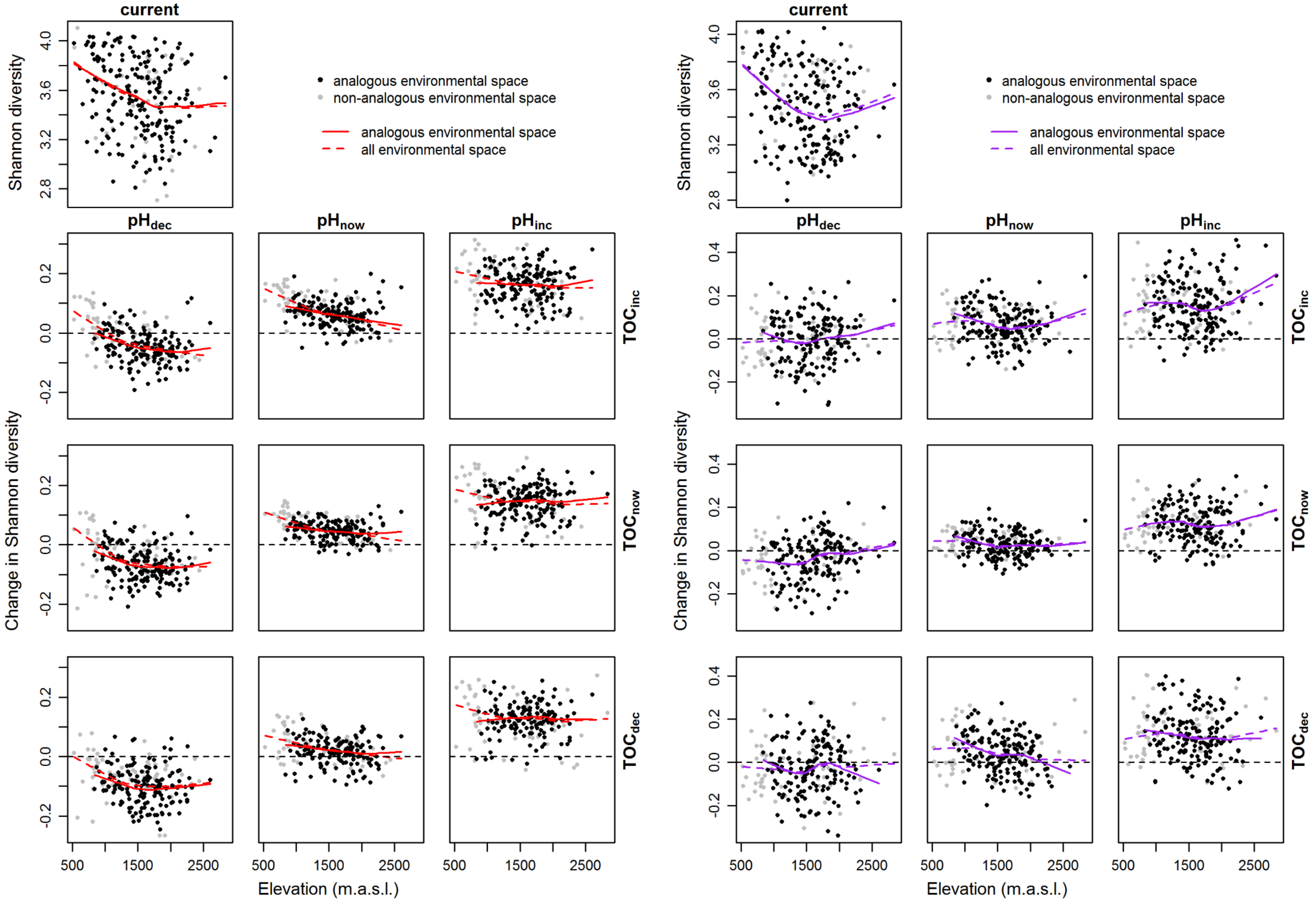


*Figure S43. Based on **CR: genus, GAMnb** and **GBM**, and projections to 229 individual sites shown against elevation, predicted current Shannon diversity (top-left panel), and predicted changes in diversity under nine different future scenarios (3×3 panels). From the figures, sites with "outliers" (+/-1.58 × IQR/sqrt(229)) are removed.*

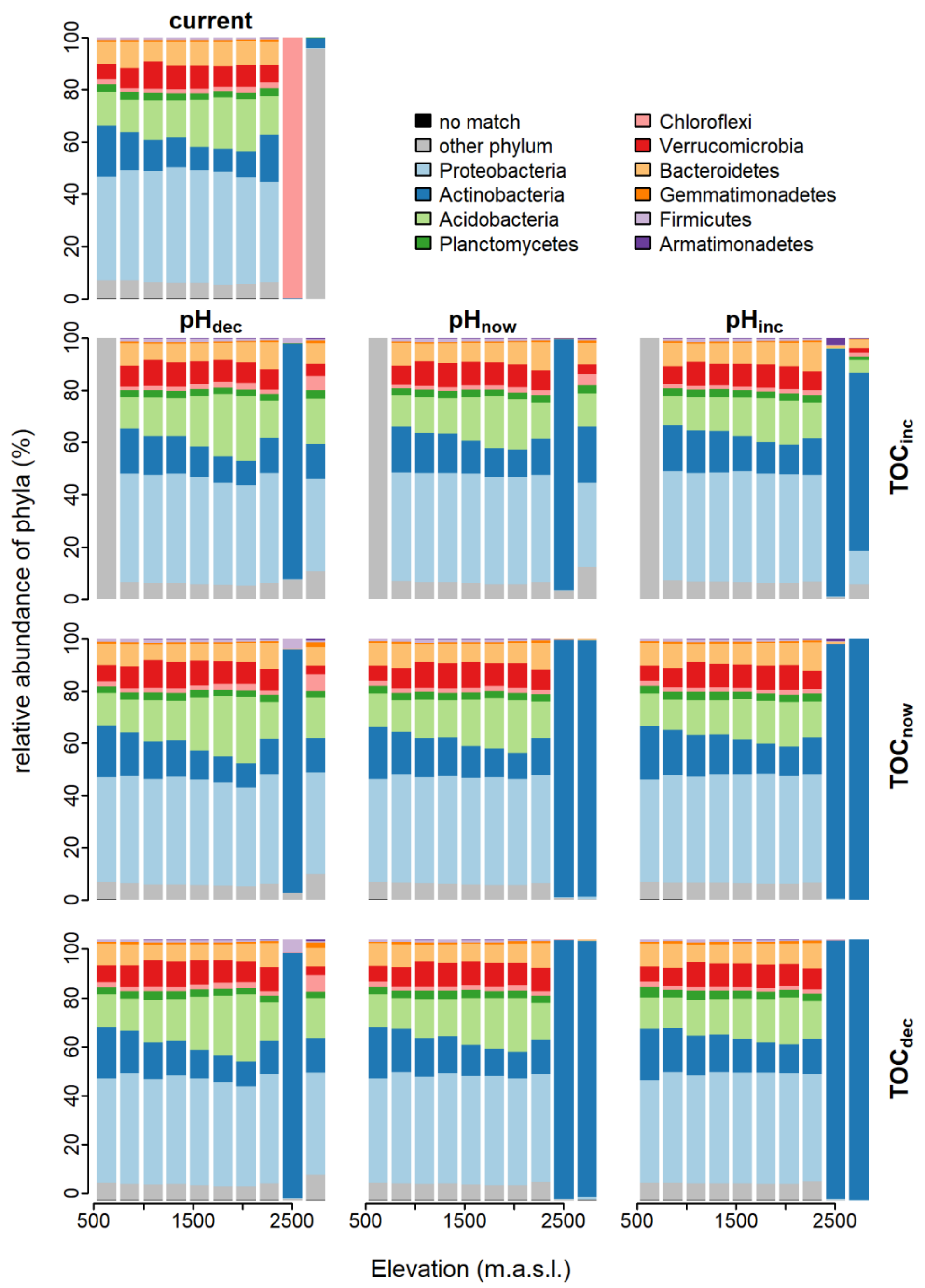


*Figure S44.*

*Based on* ***DN: cl20,*** ***GAMnb*** *(left) and* ***GBM*** *(right) and projections to 229 individual sites shown against elevation, predicted relative abundance of phyla under current conditions (top-left panels) and under nine different future scenarios (3×3 panels).*

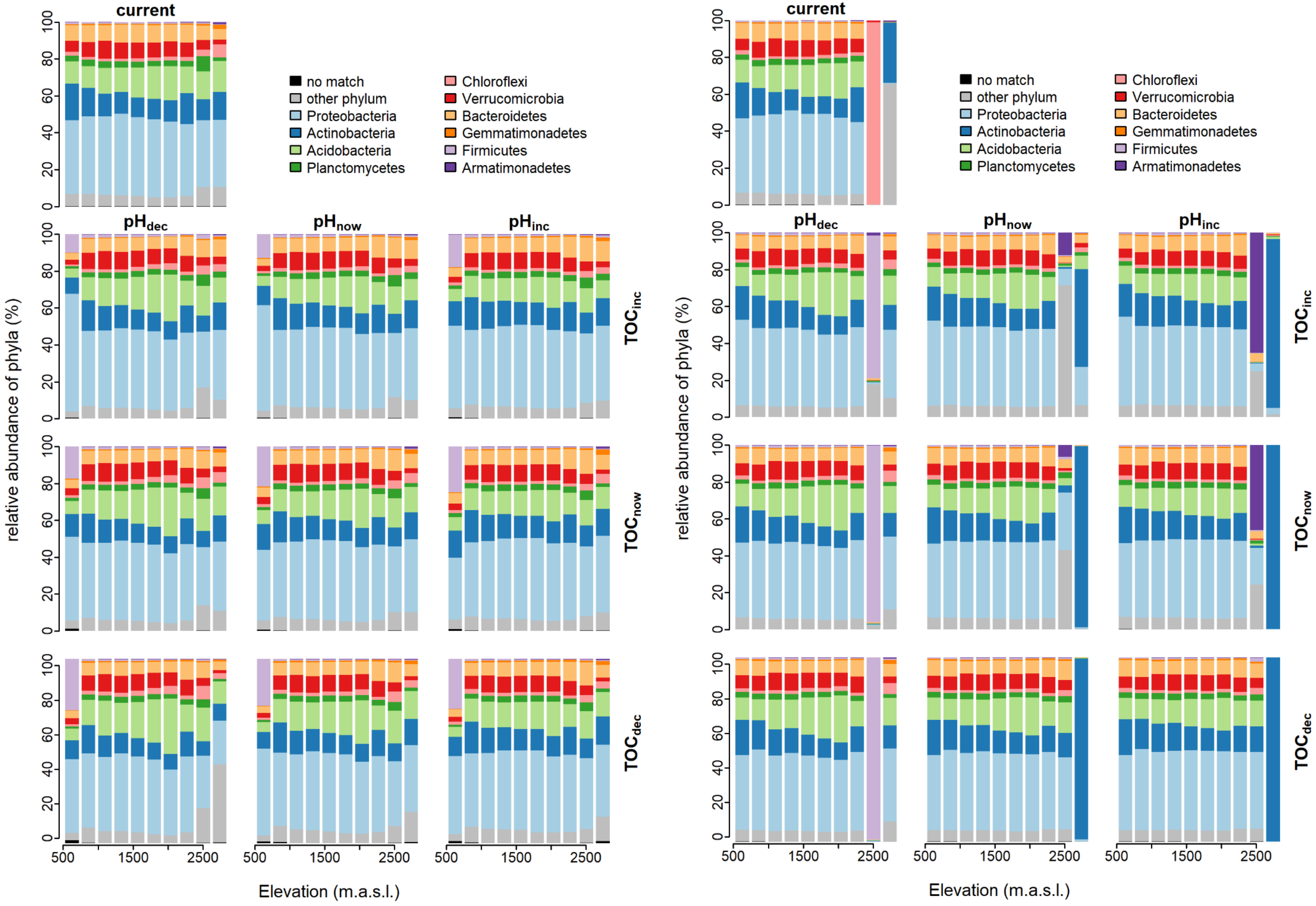


*Figure S45. Based on **DN: cl40, GAMnb** (left) and **GBM** (right) and projections to 229 individual sites shown against elevation, predicted relative abundance of phyla under current conditions (top-left panels) and under nine different future scenarios (3×3 panels).*

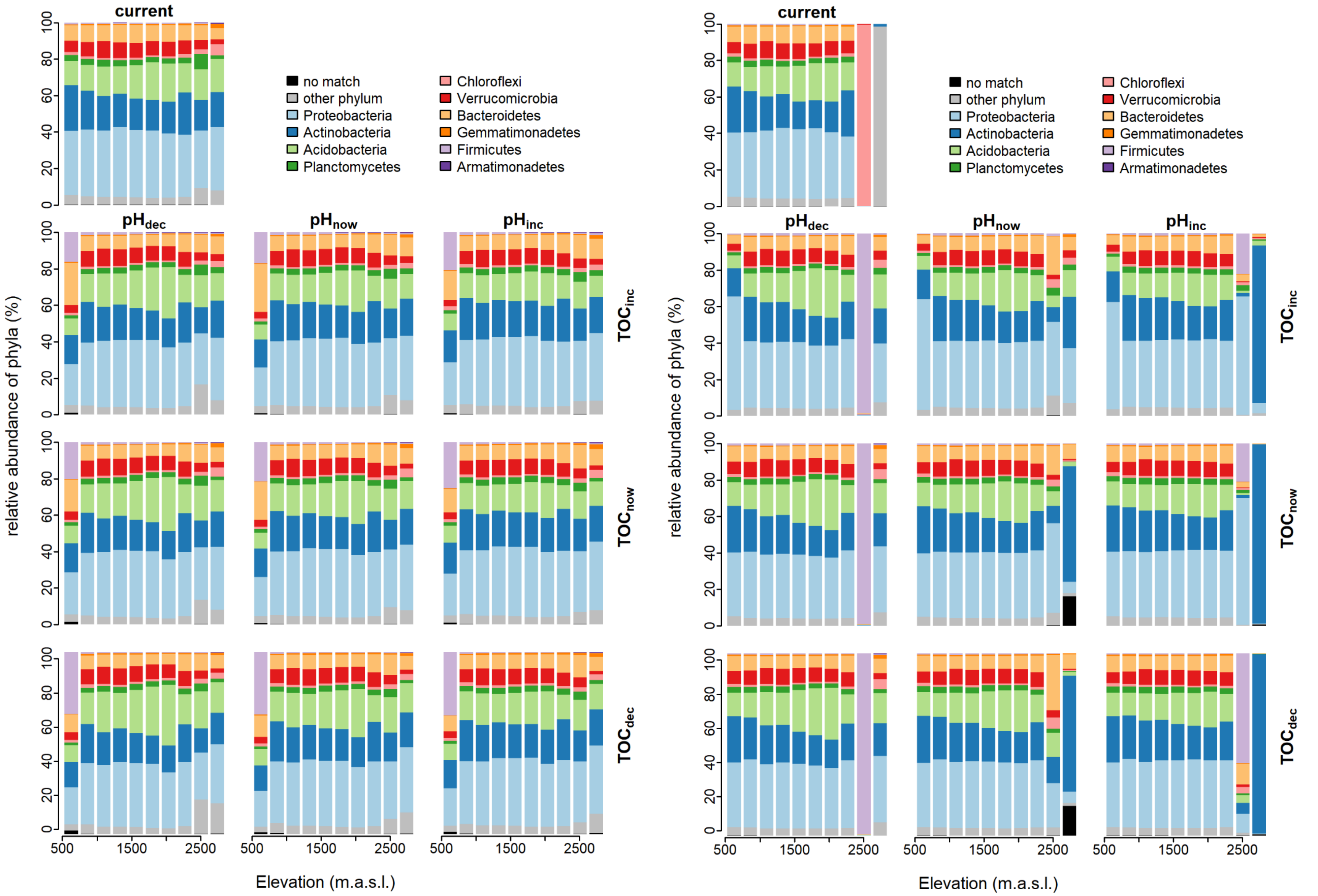


*Figure S46. Based on **DN: cl60, GAMnb** (left) and **GBM** (right) and projections to 229 individual sites shown against elevation, predicted relative abundance of phyla under current conditions (top-left panels) and under nine different future scenarios (3×3 panels).*

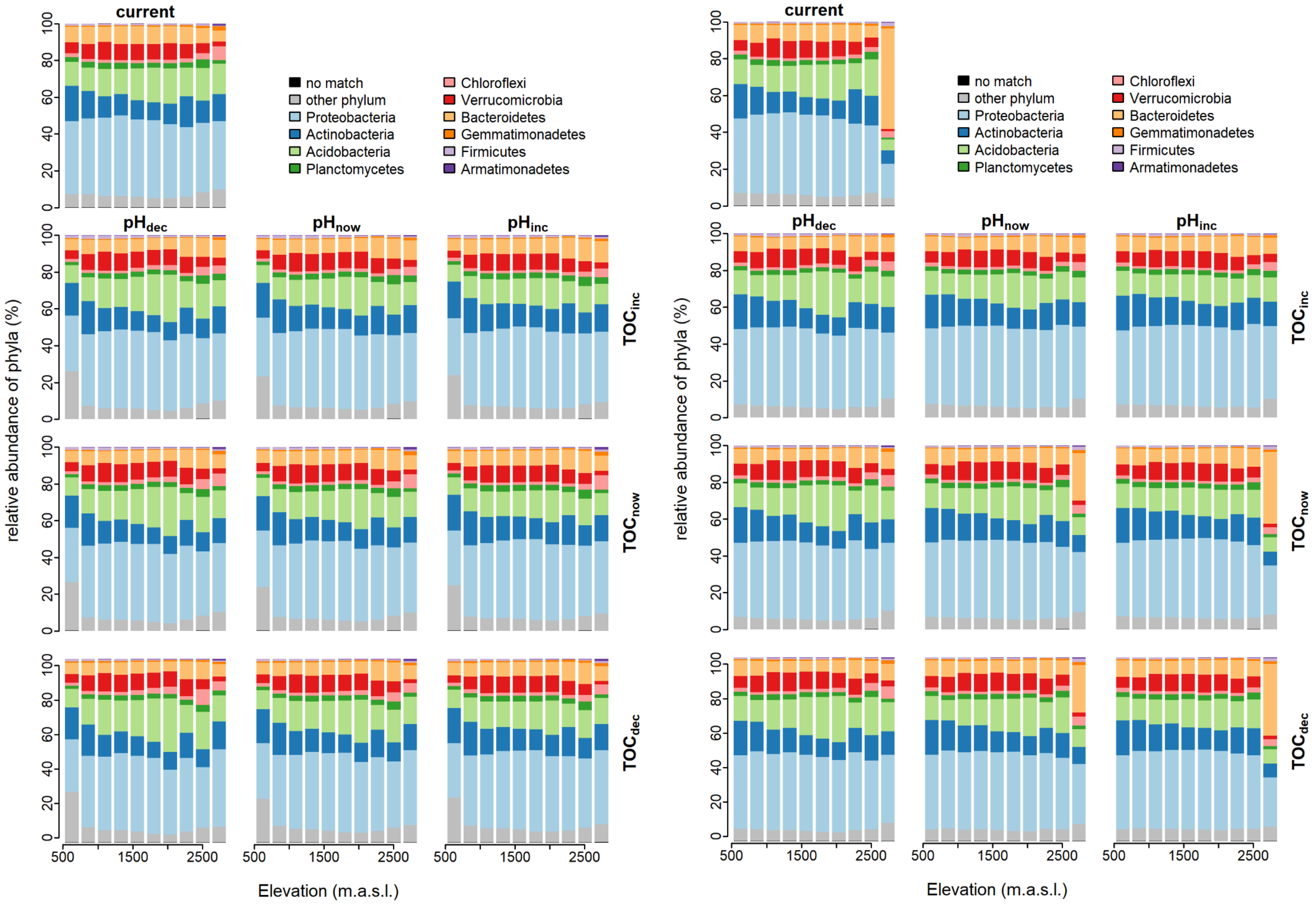


*Figure S47. Based on **DNn: cl20, GAMnb** (left) and **GBM** (right) and projections to 229 individual sites shown against elevation, predicted relative abundance of phyla under current conditions (top-left panels) and under nine different future scenarios (3×3 panels).*

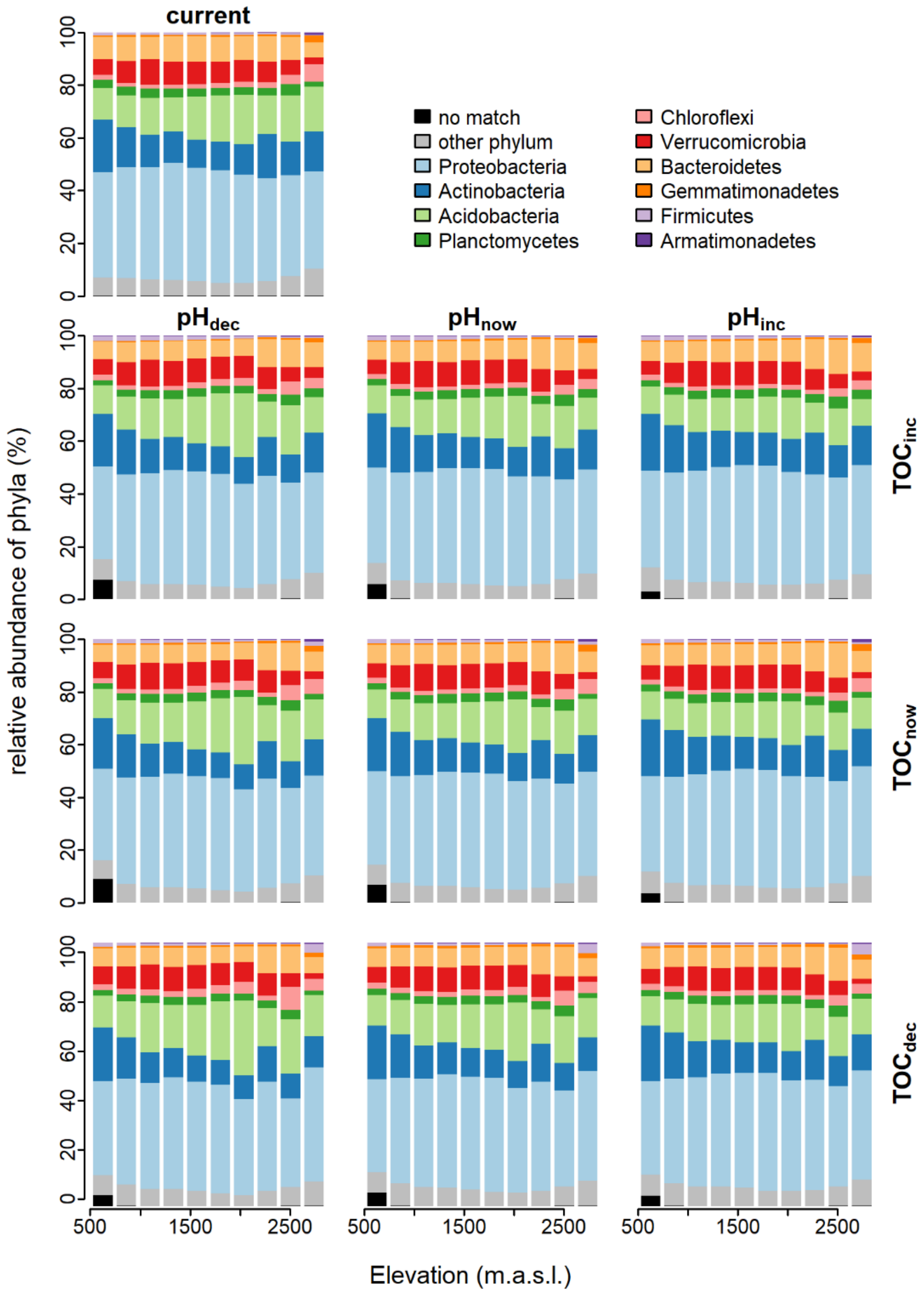


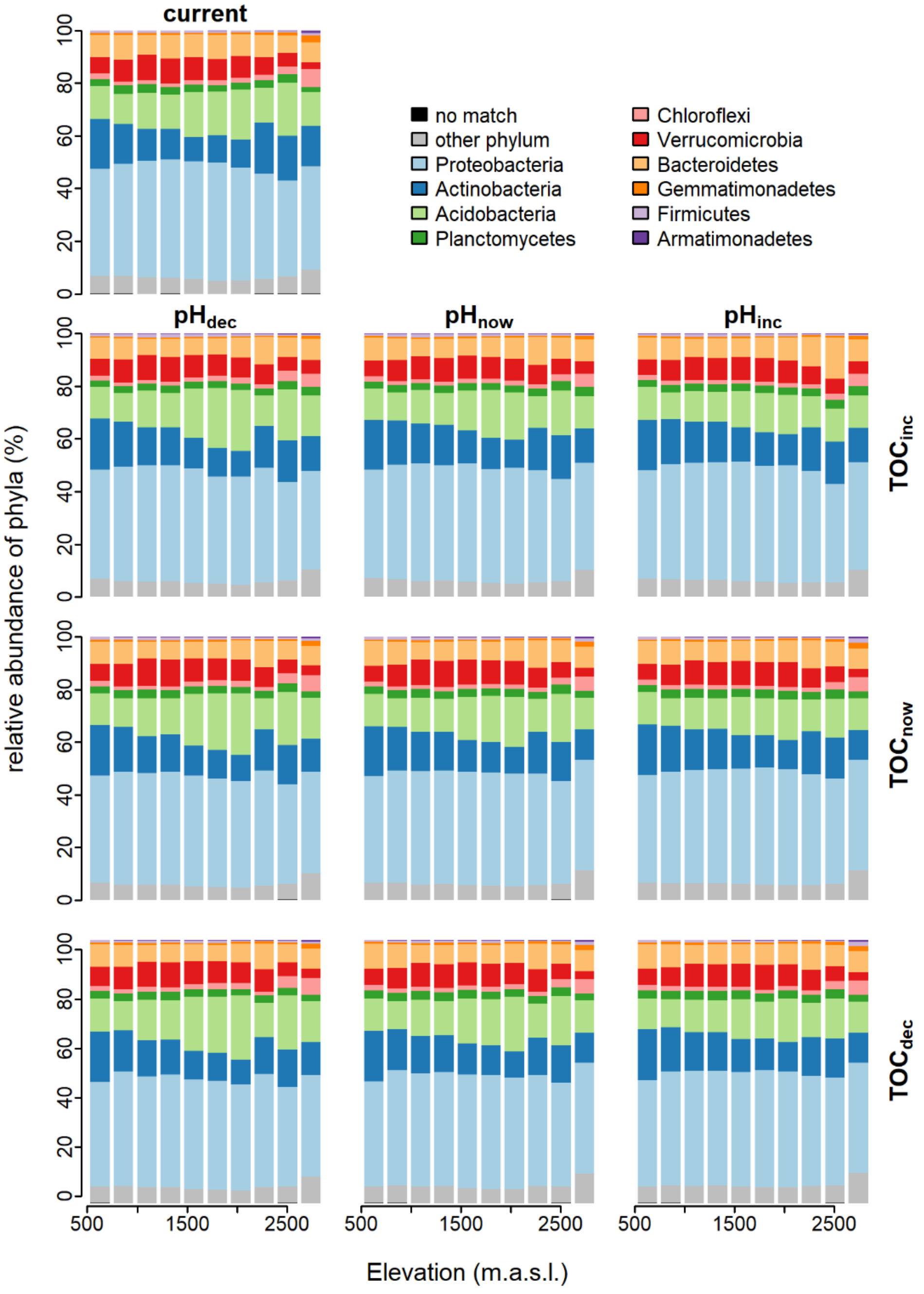


*Figure S48. Based on **DNn: cl40, GAMnb** (left) and **GBM** (right) and projections to 229 individual sites shown against elevation, predicted relative abundance of phyla under current conditions (top-left panels) and under nine different future scenarios (3×3 panels).*

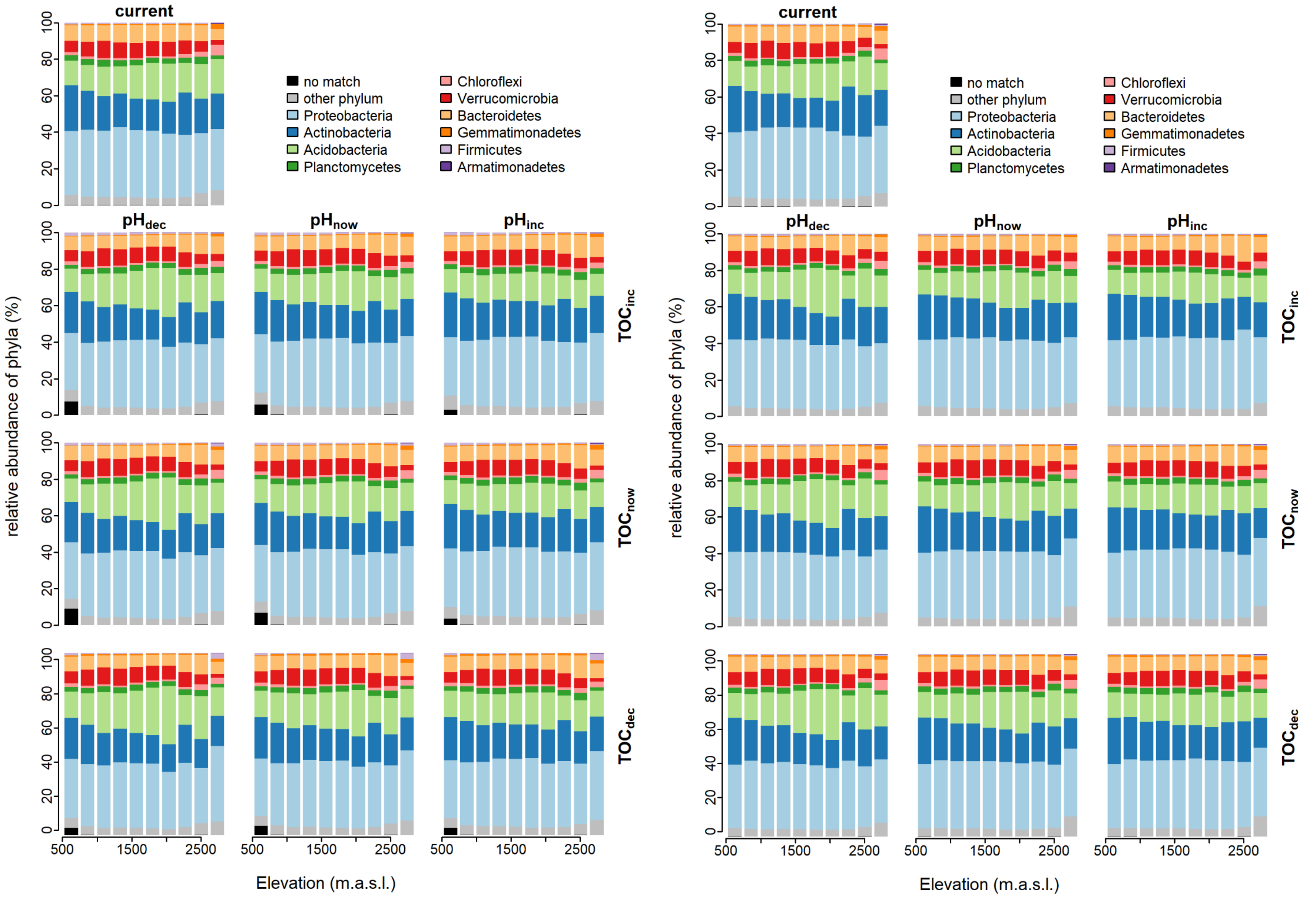


*Figure S49. Based on **DNn: cl60, GAMnb** (left) and **GBM** (right) and projections to 229 individual sites shown against elevation, predicted relative abundance of phyla under current conditions (top-left panels) and under nine different future scenarios (3×3 panels).*

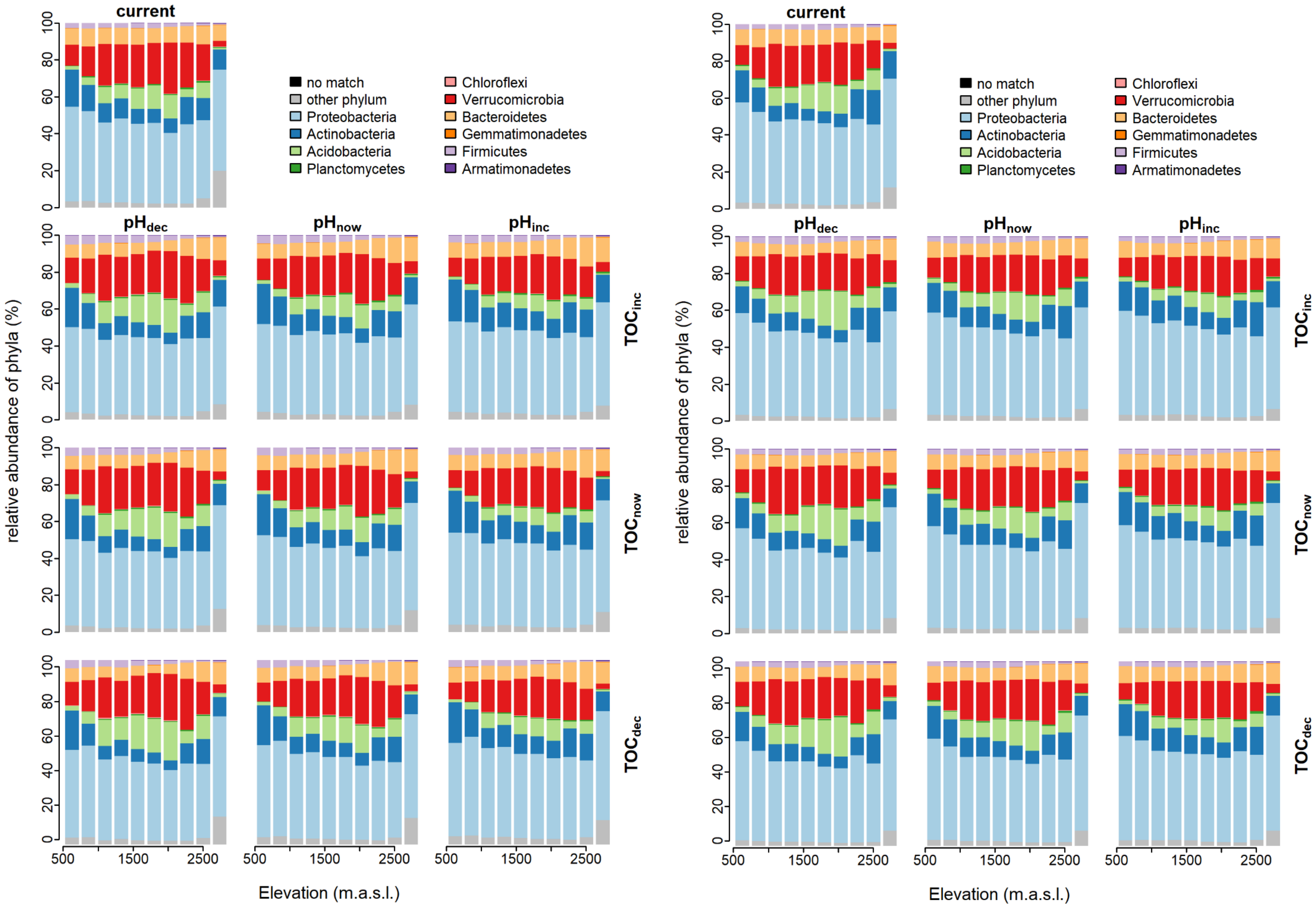


*Figure S50. Based on **CR: genus, GAMnb** (left) and **GBM** (right) and projections to 229 individual sites shown against elevation, predicted relative abundance of phyla under current conditions (top-left panels) and under nine different future scenarios (3×3 panels).*
